# Supplementary material for: Study protocol for a prospective cohort study identifying risk factors for sport injury in adolescent female football players: the Karolinska football Injury Cohort (KIC)
Source: BMJ Open. 2022 Jan 11;12(1):e055063. doi: 10.1136/bmjopen-2021-055063 (PMC8756291; doi:10.1136/bmjopen-2021-055063)
Supplement: Supplementary data [file bmjopen-2021-055063supp001.pdf]

This is a supplementary document describing the included tests in Karolinska football Injury Cohort, KIC. Table S1 shows the test stations, number of test leaders and randomization of the tests. The persons in the images have given their consent that the images will be used in publications related to this study.

Calf heel raises

Ankle plantarflexion (PF) muscle endurance is investigated using unilateral barefoot weight bearing calf heel raises[46]. Firstly, the player's maximal weight being PF range of motion (ROM) is obtained by painting a reference mark on the player's heel at floor level and registering the maximal height achieved during one calf heel raise with a metric ruler.

The player is thereafter instructed to perform repeated maximum unilateral heel raises until failure, guided by a metronome to standardize the pace (1 second concentric-, 1 second eccentric contraction). The player is allowed to have light contact with her fingers against a wall. A repetition is considered approved on the basis whether knee extension is maintained, and the reference mark on the player's heel levels with the registered maximal PF ROM height on the ruler. The test leader registers the total number of approved repetitions and discontinues the test when the player fails to reach the marked maximal height. The same procedure is then conducted on the opposite foot. The order of execution is randomized prior to the test.

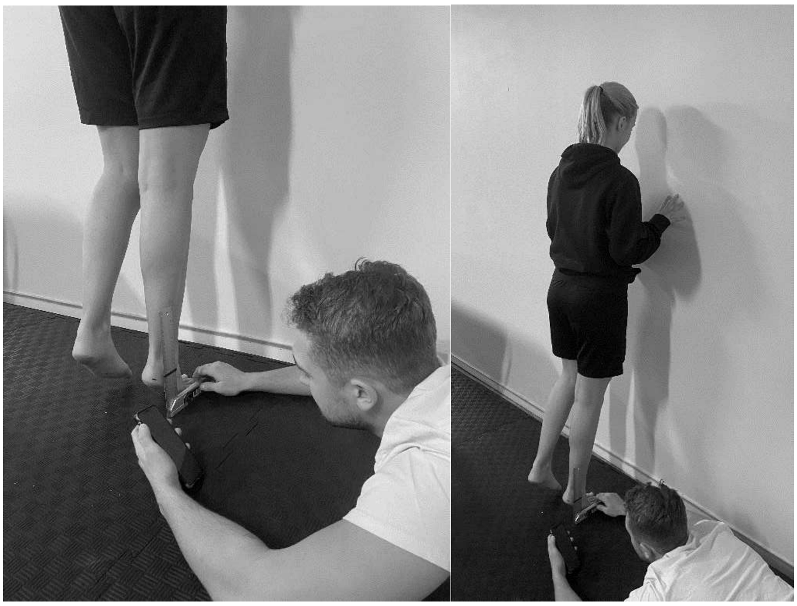

Figure S1. Calf heel raises.

Active plantarflexion mobility

Active PF ROM is measured with a clear plastic goniometer positioned at the lateral malleolus, utilizing fibula and fifth metatarsal as reference marks[47 48]. The player is positioned in supine on an treatment table, with feet off the edge of the table. The player is instructed to perform a sequence of six maximal active PF cycles starting from a neutral dorsiflexion (DF) position, whilst maintaining extended knees throughout the movement. The test leader measures and registers the maximal PF ROM in the final three cycles.

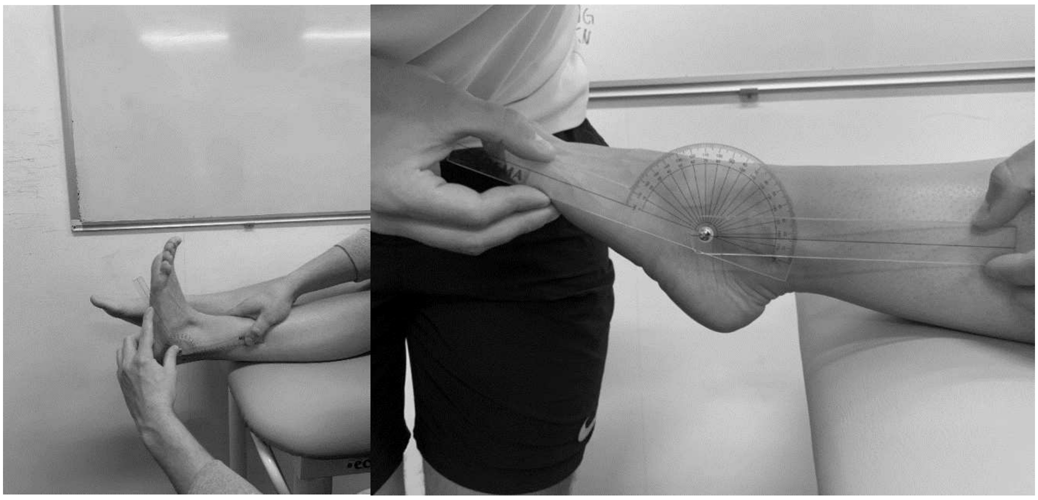

Figure S2. Active plantarflexion mobility execution. a) starting position in neutral dorsiflexion, b) end position in maximal active plantarflexion.

Weight bearing ankle dorsiflexion mobility

Weight bearing ankle DF ROM is measured in a standing lunge position with the player's foot placed upon a metric ruler 10 cm away from a wall to the player's greater toe[46 49]. The player is instructed to lunge forward, directing the knee in line with her second toe, until contact with the wall is achieved; without allowing the heel to lift off the ground, which is continuously monitored through the availability to maintain a piece of paper against the foundation. Throughout the test, the player is allowed to provide balance by light contact with her fingers against the wall.

Firstly, three consecutive warm-up trials are performed from the 10 cm mark to familiarize the player with the test. Thereafter, the test leader measures the following three trials. In each trial, the player begins from the reference mark (10 cm) and progresses 1 cm away from the wall at a time, until unable to perform a successful repetition. If the player is unable to perform an approved repetition at the 10 cm reference mark, she is asked to progress 1 cm forward until able to complete a successful repetition. Once the player achieves knee-wall contact, the DF ROM is measured with a digital inclinometer (Clinometer, Plaincode, Stephanskirchen, Germany) and the distance from the wall to the greater toe is measured in cm in the repetition furthest away from the wall in each trial.

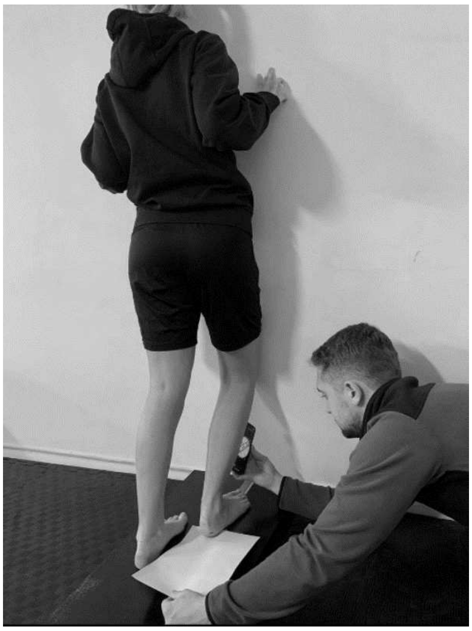

Figure S3. Weight bearing ankle dorsiflexion mobility.

Trunk mobility

Mobility in trunk rotation are measured in a cross-legged seated position, and in a lunge position with the player on a gym mat, graded with 5 degrees increments, from zero to one hundred and eighty degrees[50-52].

In the seated test (modified seated rotation test), the player is positioned at the center of the gym mat, in a cross-legged position with a wooden stick resting on the shoulders whilst keeping her arms crossed. If the player is unable to achieve the cross-legged sitting position, she is allowed to sit comfortable in an ordinary sitting position, which is noted by the test leader. Once in the starting position, the player is instructed to keep an upright posture and maximally rotate alternating between right and left for three times, whilst the test leader measures the rotational degrees in the end range.

The same procedure is thereafter repeated in a lunge position with the wooden stick resting on the player's shoulders. The player is positioned in a lunge position with her posterior knee at the center of the gym mat, and with her feet aligned on the zero-degree mark. Three consecutive maximal rotations are carried out alternating between right and left and is conducted in a lunge position for both the dominant, and non-dominant limb.

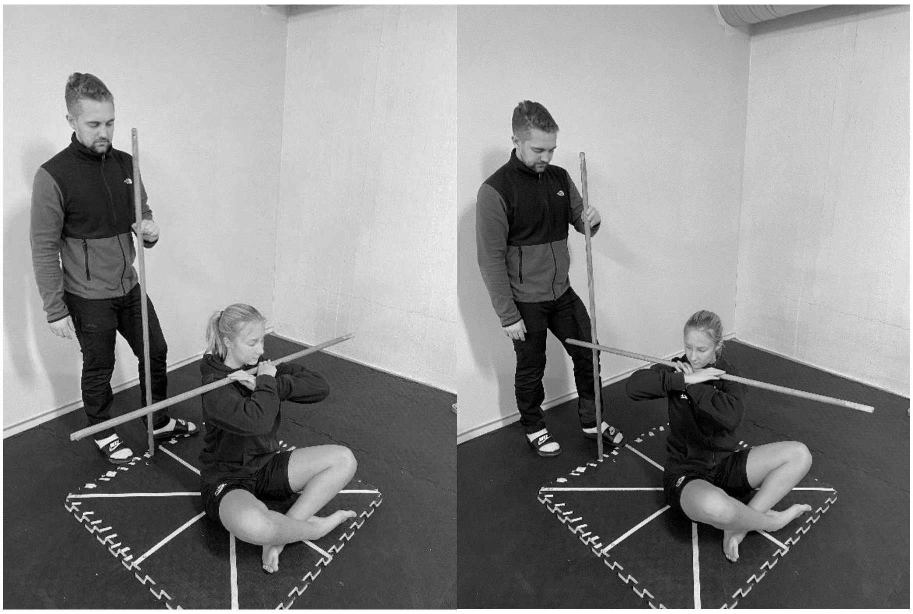

Figure S4. Modified seated rotation test. a) starting position, b) end position (right).

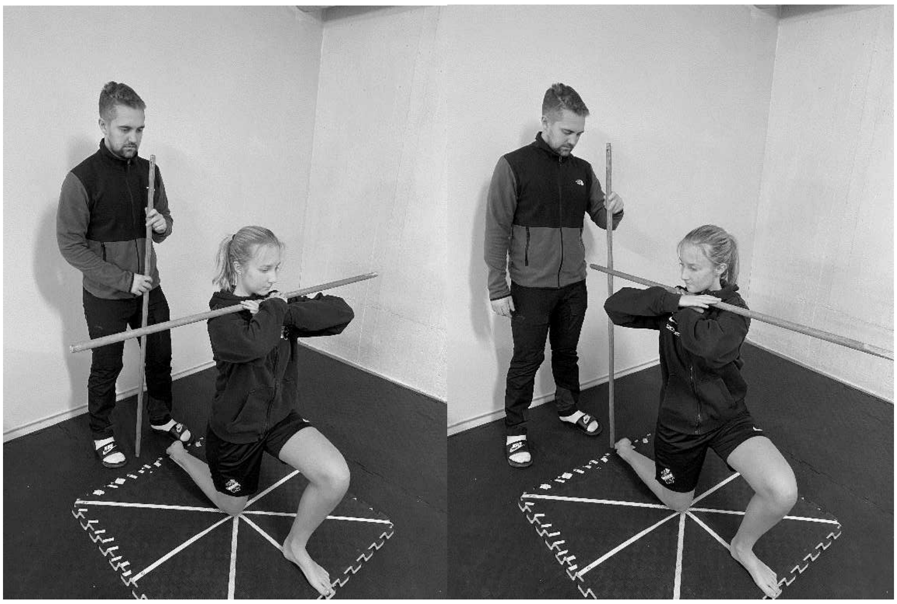

Figure S5. lunge rotation test. a) starting position left leg, b) end position (right).

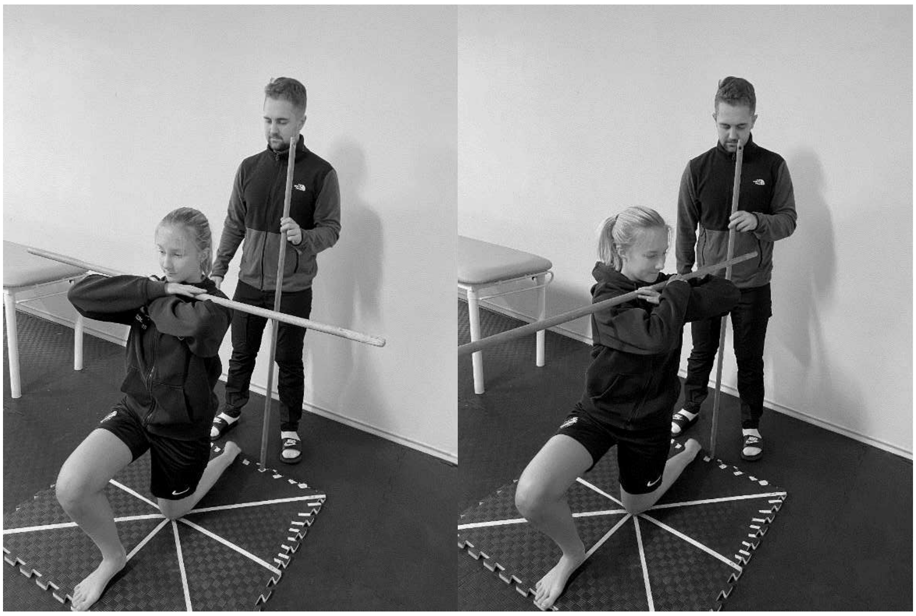

Figure S6. lunge rotation test. a) starting position right leg, b) end position (left).

Trunk strength

Isometric trunk rotational strength is measured in a modified standing wood chopper test utilizing a force gauge to evaluate force output (RS Pro Digital Force Gauge, RS Components Ltd., Corby, UK) [53-55].

In a standing position with extended arms, the player holds a handle in shoulder height, which is attached to the force gauge. The test leader positions the player in a 30-degree trunk rotation in the horizontal plane towards the anchor point (see figure S7).

The player is thereafter instructed to maximally generate force through her trunk and isometrically rotate in the opposite direction for five seconds whilst maintaining straight arms. Three consecutive repetitions are conducted for both right and left, and the maximal force output generated is used in the analyses. The order of execution is randomized prior to performing the test.

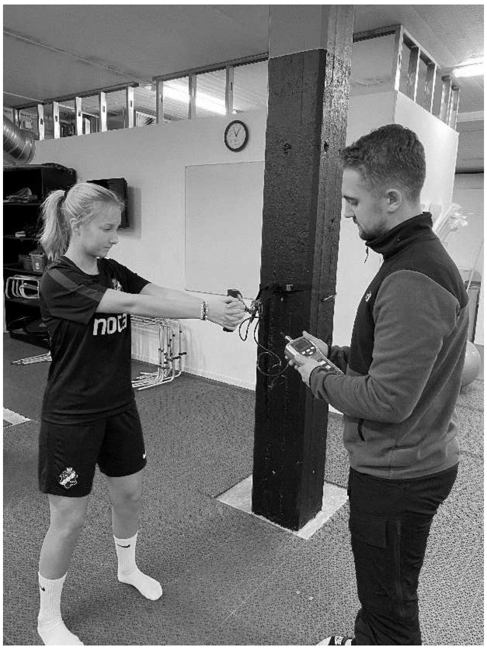

Figure S7. Modified standing wood chopper test (isometric rotation to the right).

Deep neck flexor endurance

Deep neck flexor muscle endurance is assessed through a modified version of the Cranio-cervical flexion test (CCFT) with a pressure sensor (Stabilizer Pressure Bio-Feedback, Chattanooga Group inc, Hixon, TN) [51 56 57].

Prior to executing the test, the player is instructed in how to perform a correct cranio-cervical flexion motion in standing and supine position through a gentle ‘head nodding’ cue. The player is positioned in supine position on a treatment table with her hands placed upon her abdomen or at the side of the body and with her feet on the table, with flexed hips and knees. With the player’s head and neck in a neutral position, the pressure stabilizer is positioned sub-occipitally, and inflated to a baseline pressure of 20 mmHg. Firstly, a pre-test is conducted and later an endurance test.

During the pre-test, the player is instructed to perform a gentle cranio-cervical flexion to increase the pressure starting from a baseline of 20 mmHg with 2 mmHg increments to a maximum of 30 mmHg. 3x3 second contractions are carried out at each target pressure (TP) with a three second rest in between each contraction whilst the test leader monitors for potential compensational strategies: excessive use of global neck musculature, chin jerking, cervical spine retraction, jaw clenching, breath holding and a pressure loss of ≥ 2 mmHg. A stopwatch time the contractions and visual feedback of pressure level is provided by the test leader who holds the manometer dial so that both the player and the test leader can read it throughout the procedure.

The endurance test is conducted if the player completes each of the five TP (22, 24, 26, 28 and 30 mmHg) without exhibiting any of the compensational strategies and/or experiencing pain during the pre-test. During the endurance test, the same setup and procedure as in the

pre-test is carried out. The player is now instructed to hold each contraction at the TP for 3x10 seconds with a ten second rest in between contractions. The highest completed TP with a full set of 3x10 seconds contractions is registered by the test leader and later used for analysis.

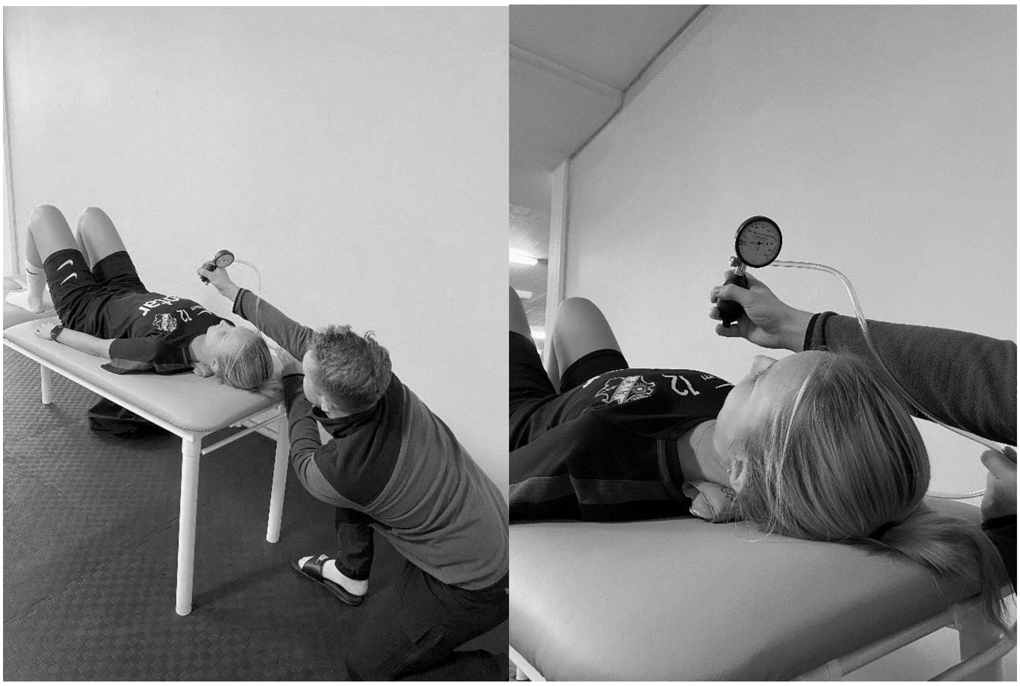

Figure S8. Modified cranio-cervical flexion test.

Hip- and knee strength

Isometric hip flexion, extension, adduction, and abduction strength as well as eccentric hip abduction and adduction and isometric knee extension strength are measured with a hand-held dynamometer (HHD) (MicroFet2, Hoggan Health Industries inc. West Jordan, UT, USA)[58 59].

Prior to executing the strength tests, two submaximal isometric contractions in each direction are performed to familiarize the player with the procedures. Three isometric contractions with gradually increasing force output for five seconds, and three maximal eccentric contractions for three seconds are performed in the isometric and eccentric tests, respectively, with a 10 second rest in between each contraction. The maximal force output for each position is registered by the test leader and later used for analysis. The order of execution and starting side is randomized prior to performing the tests at the particular test station (see table S1).

Isometric hip flexion strength

The player is positioned in a seated position at the edge of an treatment table, with 90-degrees of hip- and knee flexion. The HHD is positioned two centimeters proximal to the patella, and are externally fixated with a belt, which is secured under the leg of the treatment table, limiting hip flexion movement. The player is instructed to perform three isometric contractions on each leg.

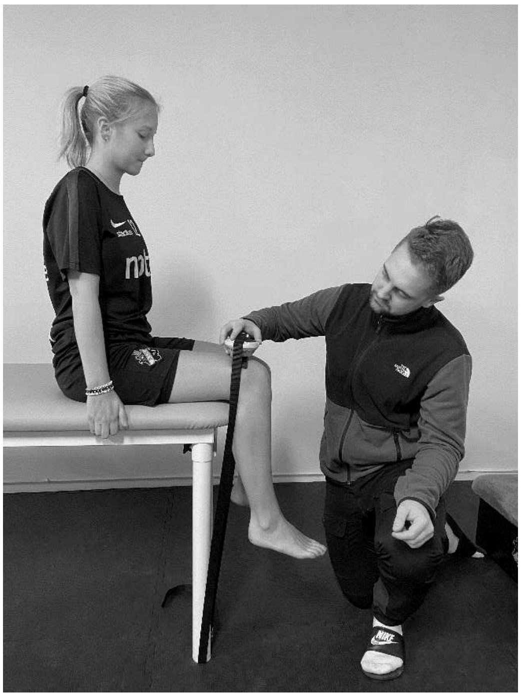

Figure S9. Isometric hip flexion strength (right hip).

Isometric knee extension strength

Seated in the same position as during the isometric hip flexion strength test, with a slightly extended knee joint, the HHD is positioned two centimeters proximal to the malleoli on the anterior aspects of the player's tibia and are externally fixated with a belt. The player is instructed to perform three isometric contractions on each leg respectively.

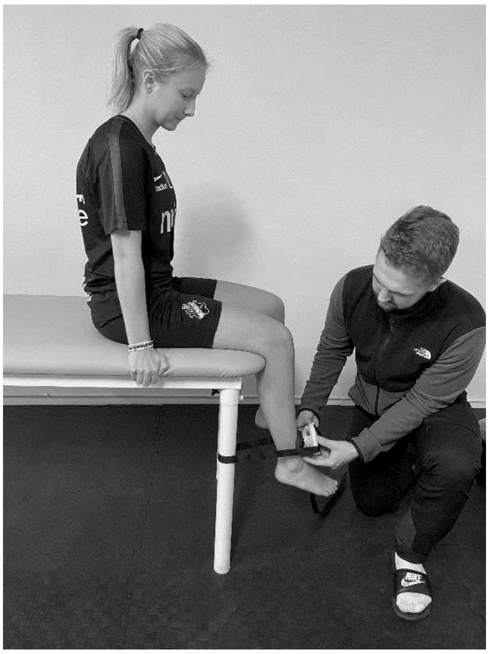

Figure S10. Isometric knee extension strength (right leg).

Isometric hip extension strength

With the player positioned in a prone position on a treatment table and with her feet off the edge of the table, the test leader externally fixates the HHD two centimeters proximal to the malleoli with a belt. Furthermore, the player is instructed to perform three maximal isometric contractions on each leg respectively

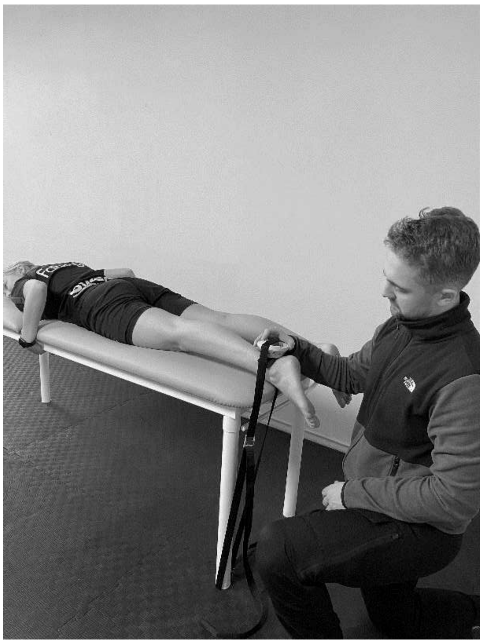

Figure S11. Isometric hip extension strength (left hip).

Isometric hip abduction strength

The player is positioned in a supine position on an treatment table, with the tested leg extended, and the non-tested leg flexed. The test leader positions and fixates the HHD two centimeters proximal to the lateral malleolus with a belt, which limits hip abduction movement. Thereafter, the player is instructed to perform three maximal isometric hip abductions, whilst the test leader measures the force output for both the left, and right side.

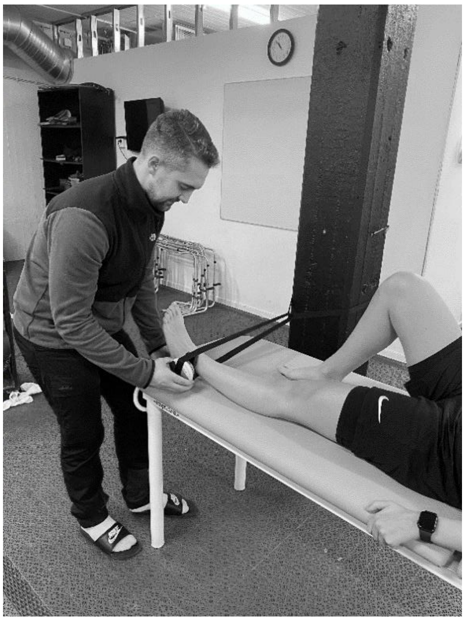

Figure S12. Isometric hip abduction strength (left hip).

Isometric hip adduction

Lying in the same position as during the isometric hip abduction the test leader places and fixates the HHD two centimeters proximal to the medial malleolus with a belt. Consequently, the player executes three maximal isometric hip adductions on the left and right side, whilst the test leader registers the force output.

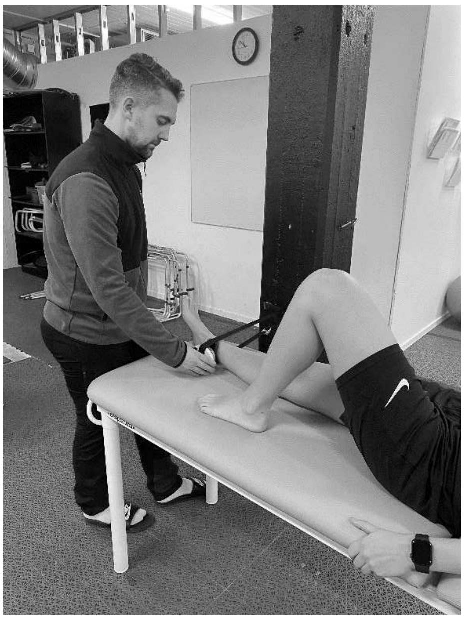

Figure S13. Isometric hip adduction strength (right hip).

Eccentric hip abduction strength

The player is in a side-lying position on an treatment table with the test leg extended, and the opposite leg flexed to 90 degrees in the knee- and hip joint, whilst a neutral hip position is maintained. The player is subsequently instructed to place the test leg in approximately 40 degrees of hip abduction, and the test leader places a HHD one centimeter proximally to the lateral malleolus. The test leader initiates the test by saying "push", and when the player has built up a maximal isometric contraction, the test leader begins to apply a downward directed force with the HHD whilst the player resists eccentrically for five seconds. Three repetitions are carried out on both the right and leg left, and the maximal force output is later used for analysis.

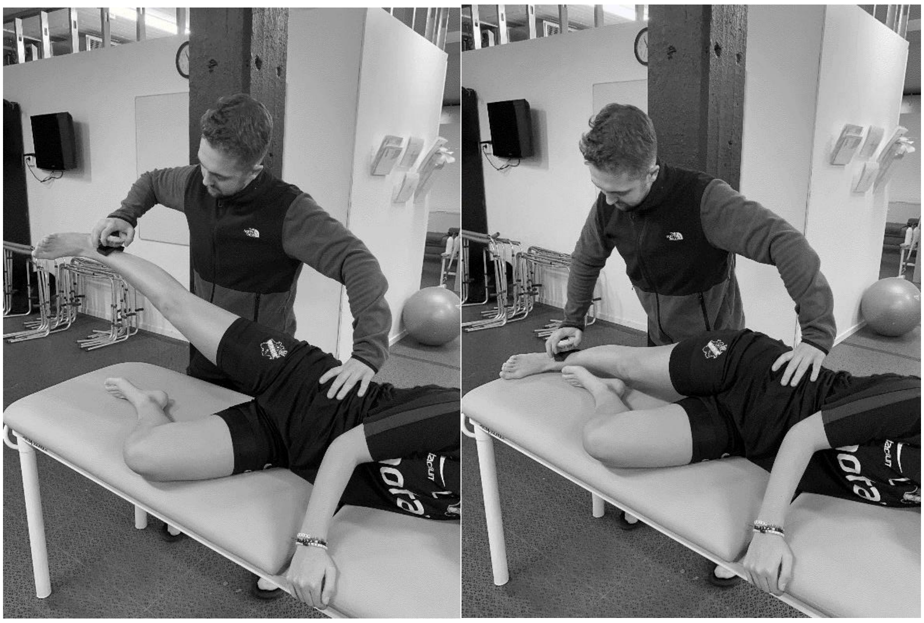

Figure S14. Eccentric hip abduction strength. a) starting position (right hip), b) end position (right hip).

Eccentric hip adduction strength

The player is positioned in the same manner as in the eccentric hip abduction strength test, with the tested leg extended, and the non-tested leg flexed in the hip- and knee joint. Thereafter, the player is instructed to place the test leg in a maximal adduction position, whereupon the test leader positions a HHD one centimeter proximally to the medial malleolus. The test is initiated when the test leaders says "push", whereupon a downward directed force is applied with the HHD whilst the player resists eccentrically for five seconds. Three consecutive trials are conducted on both sides, and the test leader registers the force output.

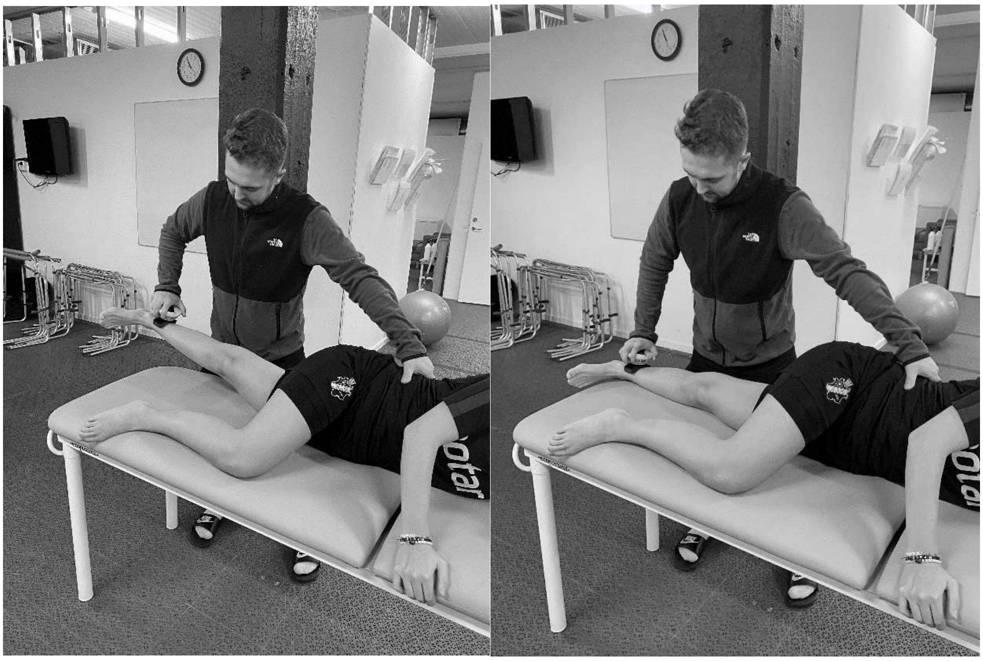

Figure S15. Eccentric hip abduction strength. a) starting position (left hip), b) end position (left hip).

Hip mobility

Measures of passive hip ROM in flexion, extension, abduction, internal- and external rotation are obtained using a universal clear plastic goniometer[60 61]. Three consecutive measurements for each position are performed for both the dominant and the non-dominant leg and the mean value for each position is later used for analysis. If the same value is obtained during the first and second measurement for a particular movement, a third one is not performed. The order of execution (side and movement) is randomized prior to performing the measures.

Passive hip flexion ROM

The player is positioned in supine position on a treatment table. With the player's leg held in a 90-degree knee flexion, test leader 1 moves the player's leg into a passive hip flexion until a firm end feel is achieved, and a posterior pelvic tilt occurs. Once the end feel is achieved, test leader 2 places the center of the goniometer at the greater trochanter and aligns one of the goniometer's arms with the player's femur, and the other one horizontally with the treatment table to read the goniometer. Three consecutive measures are conducted on each hip.

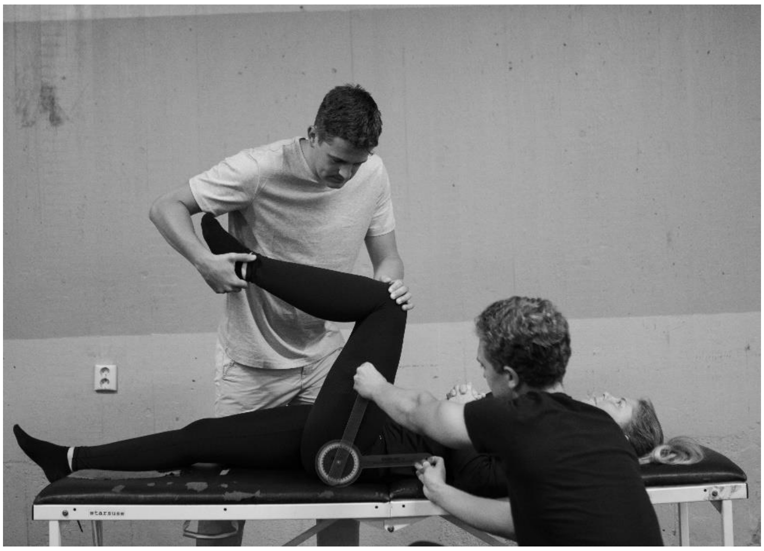

Figure S16. Passive hip flexion ROM (left hip).

Passive hip abduction ROM

The player is in a supine position on an treatment table with extended legs. While palpating the player's ipsilateral anterior superior iliac spine, test leader 1 holds the player's leg by the ankle and moves the leg into passive hip abduction until a firm end feel is achieved, and motion is felt at the pelvis. Thereafter, test leader 2 positions the goniometer at the player's hip, aligning the lever arms with the player's anterior superior iliac spine and femur, and reads the degrees of abduction. The test is repeated three times on each hip.

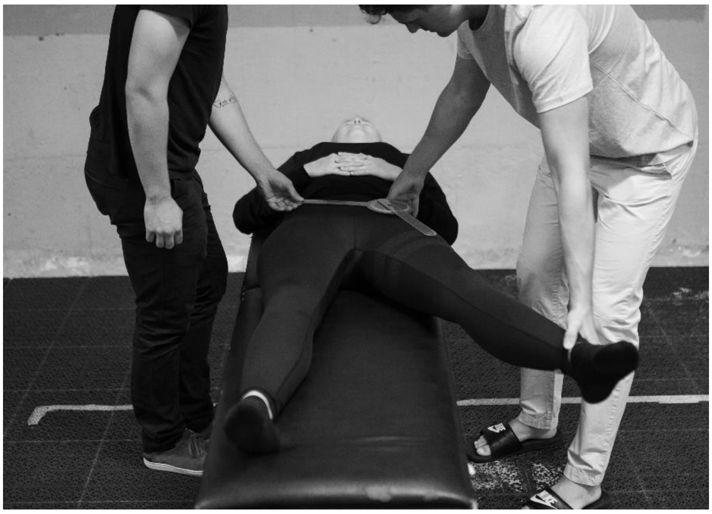

Figure S17. Passive hip abduction ROM (left hip).

Passive hip extension ROM

In prone position with extended legs, test leader 1 fixates the player's pelvis by placing a hand at the ipsilateral posterior superior iliac spine. Thereafter, while holding the player's leg at the knee, test leader 1 moves the player's leg into passive hip extension, until an end feel is achieved, indicated by an anterior tilt of the pelvis. Test leader 2 measures the degrees of passive hip flexion with the goniometer's center positioned at the greater trochanter, and the lever arms in line with the player's femur and the treatment table horizontally. Three measures are performed on each leg.

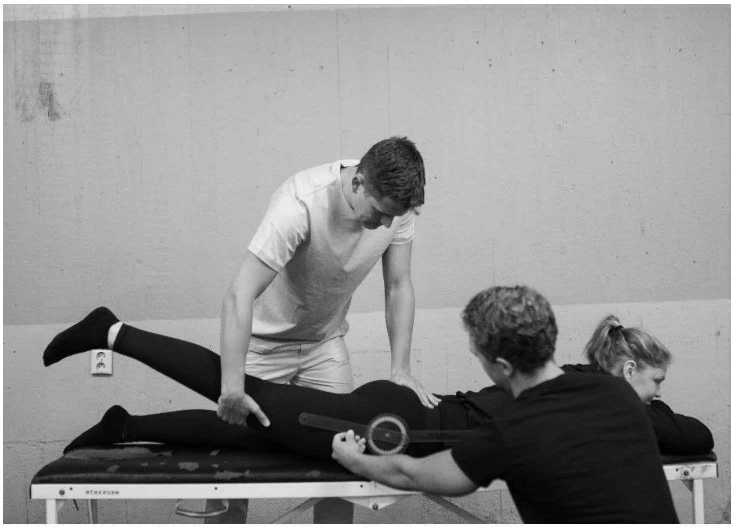

Figure S18. Passive hip extension ROM (right hip).

Passive hip internal- and external rotation ROM

In prone position, the player's leg is flexed to 90 degrees in the knee joint. Consequently, test leader 1 fixates the pelvis by placing his/her hand on the player's posterior superior iliac spine and performs a passive internal and external hip rotation, respectively, until an end feel is felt, indicated by an anterior pelvic tilt. Test leader 2 measures the degree of rotational mobility with a goniometer positioned at the knee, with the levers aligned with the player's tibia and with the treatment table horizontally. Three consecutive measures are conducted on each leg.

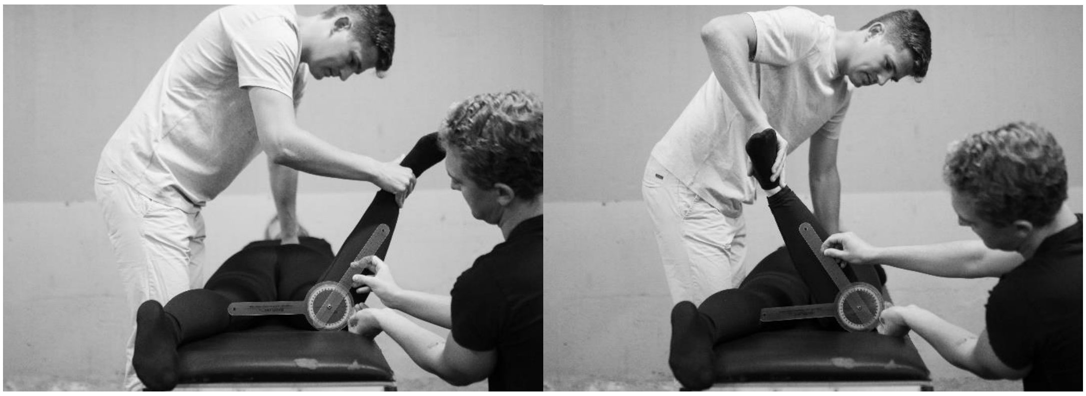

Figure S19. Passive hip rotational ROM (right hip). a) internal rotation, b) external rotation.

Jump performance tests

To assess the player's unilateral jump performance, the One-leg Long Box Jump Test (OLLBJ) and square hop test are performed[62 63]. A 40x40 cm square is marked on the foundation and later utilized as a reference mark in both tests. During the jump tests, players wear indoor sporting shoes.

One-leg long box jump test (OLLBJ)

Firstly, the starting position, i.e. the distance player's jump from to the 40x40 cm square is calculated by dividing the player's height in cm with 1.6 (height/1.6 = distance to the square). The player is instructed to stand on one leg at the starting position, and to perform a one-legged jump aiming inside the boundaries of the 40x40 cm square, and to maintain balance after landing. A trial is considered approved on the basis that the player land inside the 40x40 cm square, and adequately maintains balance after landing. The player performs three warm up trials on each leg, to familiarize with the procedure, and later five consecutive test trials. The test leader registers the total number of approved trials on each leg (0 to 5).

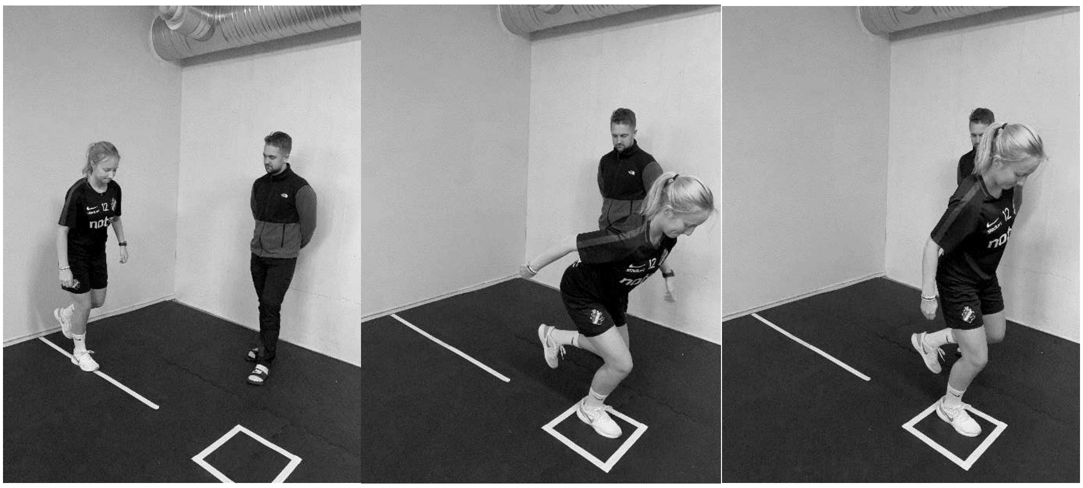

Figure S20. One-leg long box jump test (right leg). a) starting position, b) landing, c) balance maintained.

Square hop test

During the square hop test, the player is instructed to hop on one leg in and out of the 40x40 cm square as many times as possible for 15 seconds in a clockwise direction, timed with a stopwatch, whilst the test leader registers the number of approved hops. A hop is classified as approved on the basis whether the player begins a hop in the starting position (outside the square) and then executes the short hop task inside the square and then in the correct direction outside the square. Prior to the test, the player performs two warm up trials on each foot.

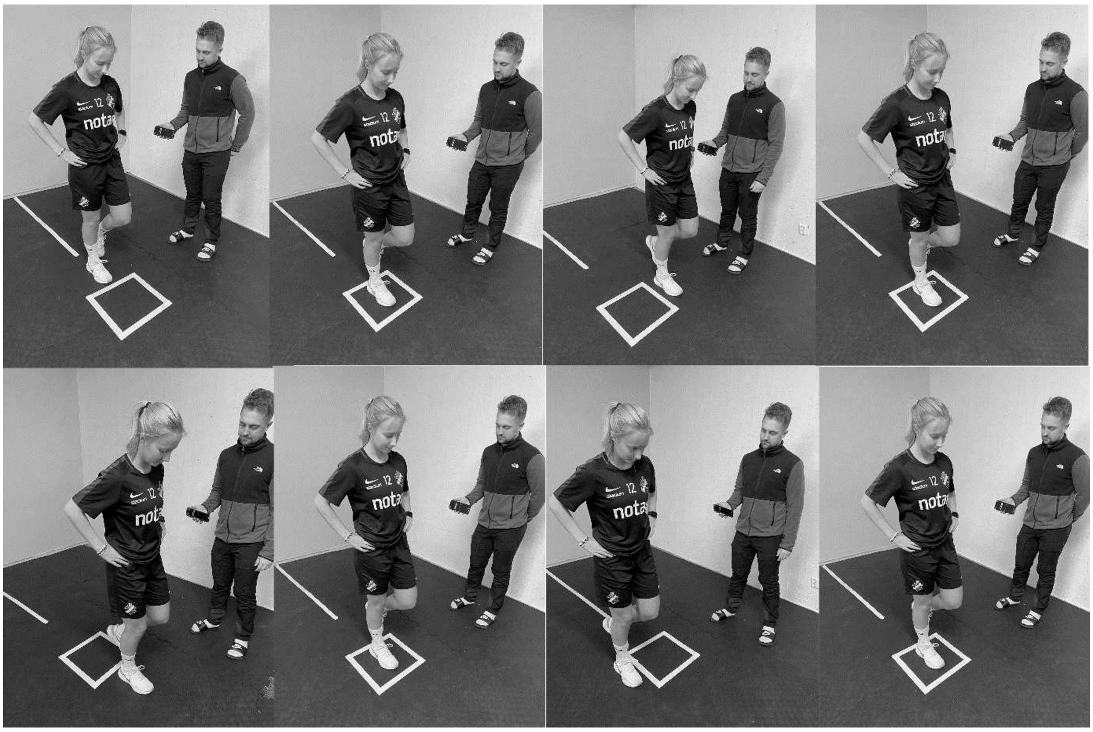

Figure S21. A series of square hop tests illustrated on the player's right leg.

Ankle- & knee stability

Modified anterior drawer test (ankle)

To assess talocrural stability or pain, a modified anterior drawer test is performed[64 65]. With the player in supine position with the test limb in knee flexion and the on the treatment table, the test leader applies an anteriorly directed force to the player's talus and a concurrent posteriorly directed force to the calcaneus. The test is conducted once on both the dominant and non-dominant foot and are considered positive if the player experiences any pain or discomfort during the procedure.

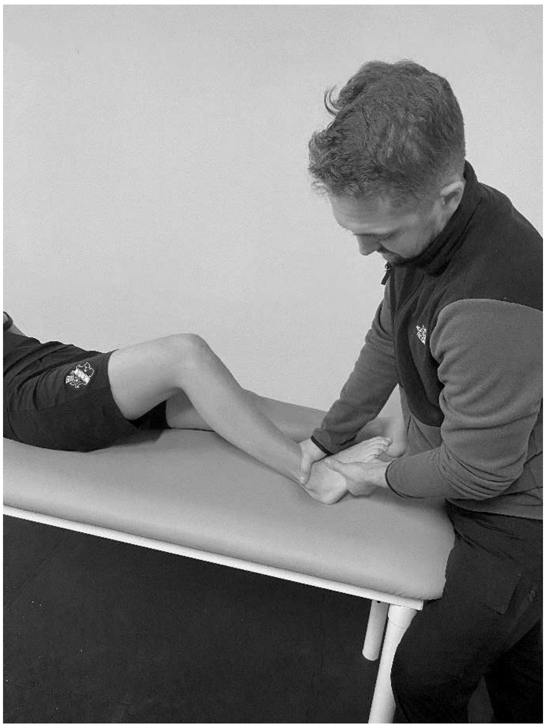

Figure S22. Modified anterior drawer test (right ankle).

Modified Fairbank's apprehension test (patellofemoral)

A modified version of Fairbank's apprehension test is conducted to evaluate stability or pain in the patellofemoral joint[66]. In supine position with extended legs, the test leader applies a laterally and subsequently medially directed force to the patella. The test is considered positive if the player experiences any pain or discomfort during the test, and/or an involuntary contraction of the quadriceps musculature. The test is carried out once on the player's dominant and non-dominant limb.

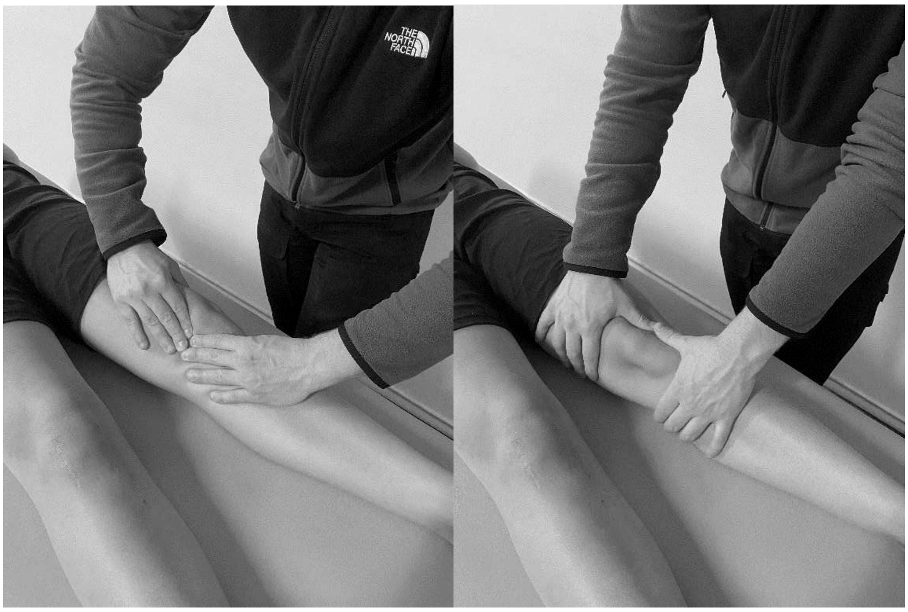

Figure S23. Modified Fairbank's apprehension test (left patellofemoral joint).
a) lateral translation, b) medial translation.

Isometric back extensor endurance

Isometric back extensor endurance is assessed by a modified Sorensen test[67-69]. In prone position, the player's anterior superior iliac spine is positioned at the edge of the treatment table. The player´s lower body is supported to the treatment table with three straps positioned over the player's ankles, knees, and pelvis. Whilst the test leader fastens the player's lower body to the treatment table with the three straps, the player uses a box/stool for support.

The player is thereafter instructed to keep her arms folded across the chest and isometrically maintain the upper body in a horizontal position until failure whilst the test leader register the time elapsed. A digital inclinometer (Clinometer, Plaincode, Stephanskirchen, Germany) is placed upon a metric ruler at the level of the 5th vertebra of the thoracical spine to monitor sagittal plane movement. If the player's upper body deviate greater than 10 degrees in the sagittal plane on more than two occasions and/or experience pain during the procedure, the test is stopped. Prior to the test, the player completes a shorter warmup trial of 5 seconds to orient the desired sagittal plane target angle.

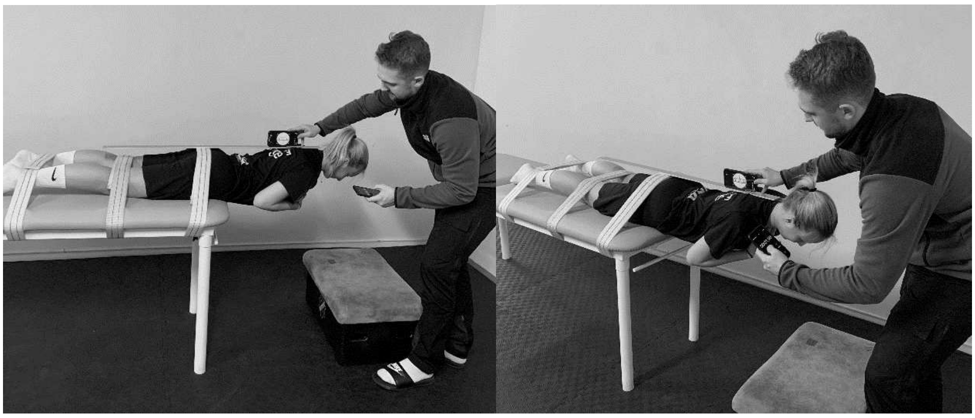

Figure S24. Modified Sorensen test.

Table S1. Test stations, number of test leaders and randomization of the physical test protocol.

Test	Test station	Number of test leaders	Randomized order of execution
Calf heel raises	1	1	Yes
Active plantarflexion mobility	2	1	No
Weight bearing ankle dorsiflexion mobility	2	1	No
Ankle- & Knee stability	2	1	No
Hip mobility	3	2	Yes
Isometric Knee extension, hip flexion & extension strength	4	2	Yes
Trunk mobility	5	1	No
Trunk strength	5	1	Yes
Isometric and eccentric hip abduction and adduction	6	2	Yes
Deep neck flexor endurance	7	1	n/a
Functional performance tests	8	1	Yes
Isometric back extensor endurance	9	1	n/a

n/a-not applicable
