# Supplementary material for: Study protocol for a prospective cohort study identifying risk factors for sport injury in adolescent female football players: the Karolinska football Injury Cohort (KIC)
Source: BMJ Open. 2022 Jan 11;12(1):e055063. doi: 10.1136/bmjopen-2021-055063 (PMC8756291; doi:10.1136/bmjopen-2021-055063)
Supplement: Author's manuscript [file bmjopen-2021-055063.draft_revisions.pdf]

BMJ Open is committed to open peer review. As part of this commitment we make the peer review history of every article we publish publicly available.

When an article is published we post the peer reviewers' comments and the authors' responses online. We also post the versions of the paper that were used during peer review. These are the versions that the peer review comments apply to.

The versions of the paper that follow are the versions that were submitted during the peer review process. They are not the versions of record or the final published versions. They should not be cited or distributed as the published version of this manuscript.

BMJ Open is an open access journal and the full, final, typeset and author-corrected version of record of the manuscript is available on our site with no access controls, subscription charges or pay-per-view fees (http://bmjopen.bmj.com).

If you have any questions on BMJ Open's open peer review process please email
info.bmjopen@bmj.com

BMJ Open

Study protocol for a prospective cohort study identifying risk factors for sport injury in adolescent female football players: the Karolinska football Injury Cohort (KIC)

Journal:	*BMJ Open*
Manuscript ID	bmjopen-2021-055063
Article Type:	Protocol
Date Submitted by the Author:	29-Jul-2021
Complete List of Authors:	Tranaeus, Ulrika; Karolinska Institutet; Swedish School of Sport and Health Sciences<br>Weiss, Nathan; Karolinska Institutet, IMM; Sophiahemmet University, Department of health promotion science<br>Lyberg, Victor; Sophiahemmet University, Department of health promotion science<br>Hagglund, Martin; Linköping University, Department of Health, Medicine and Caring Sciences; Linköping University, Sport Without Injury ProgrammE (SWIPE)<br>Waldén, Markus; Linköping University, Sport Without Injury ProgrammE (SWIPE); Hässleholm-Kristianstad Hospitals, Department of Orthopaedics<br>Johnson, Urban; Halmstad University<br>Asker, Martin; Karolinska Institutet; Sophiahemmet University, Department of health promotion science<br>Skillgate, Eva; Karolinska Institutet; Sophiahemmet University, Department of health promotion science
Keywords:	Orthopaedic sports trauma < ORTHOPAEDIC & TRAUMA SURGERY, SPORTS MEDICINE, EPIDEMIOLOGY

SCHOLARONE™
Manuscripts

Study protocol for a prospective cohort study identifying risk factors for sport injury in adolescent female football players: the Karolinska football Injury Cohort (KIC)

Ulrika Tranaeus,[1,2] (ORCID 0000-0002-2102-6352)

Nathan Weiss,[1,3] (ORCID 0000-0002-1814-020X)

Victor Lyberg,[3]

Martin Hägglund,[4,5,6] (ORCID 0000-0002-6883-1471)

Markus Walden,[5,6,7,8] (ORCID 0000-0002-6790-4042)

Urban Johnson,[9] (ORCID 0000-0003-0990-4842)

Martin Asker,[1,3,10] (ORCID 0000-0002-5387-3572)

Eva Skillgate[1,3] (ORCID 0000-0003-2096-1530)

[1] Institute of Environmental Medicine, Unit of Intervention and Implementation Research for Worker Health, Karolinska Institutet, Stockholm, Sweden

[2] The Swedish School of Sport and Health Sciences, Stockholm, Sweden

[3] Department of health promotion science, Musculoskeletal & Sports Injury Epidemiology Center, Sophiahemmet University, Stockholm, Sweden

[4] Unit of Physiotherapy, Department of Health, Medicine and Caring Sciences, Linköping University, Sweden

[5] Sport Without Injury ProgrammE (SWIPE), Department of Health, Medicine and Caring Sciences, Linköping University, Linköping, Sweden

[6] Football Research Group, Linköping, Sweden

[7] Unit of Community Medicine, Department of Health, Medicine and Caring Sciences, Linköping University, Sweden

[8] Department of Orthopaedics, Hässleholm-Kristianstad Hospitals, Hässleholm, Sweden

[9] Halmstad University, Halmstad Sweden

For peer review only - http://bmjopen.bmj.com/site/about/guidelines.xhtml

[10] Naprapathögskolan - Scandinavian College of Naprapathic Manual Medicine, Stockholm, Sweden

Corresponding author: Ulrika Tranaeus, Karolinska Institutet, IMM, SE 171 77, Stockholm, Sweden, +46 8 120 537 23, ulrika.tranaeus@gih.se ulrika.tranaeus@ki.se

Word count: 3484

ABSTRACT

Introduction Football is a popular sport among young females worldwide, but studies on injuries in female players are scarce compared with male players. The aim of this study is to identify risk factors for injury in adolescent female football players.

Methods and analysis The Karolinska football Injury Cohort (KIC) is an ongoing longitudinal study that will include approximately 400 female football academy players 13-19 years old in Sweden. A detailed questionnaire regarding demographics, health status, lifestyle, stress, socioeconomic and psychosocial factors, and various football-related factors are completed at baseline and after one year. Clinical tests measuring strength, mobility, neuromuscular control of the lower extremity, trunk, and neck are carried out at baseline. Players are followed prospectively with weekly e-mails regarding exposure to football and other physical activity, health issues (such as stress, recovery, etc.), pain, performance, and injuries via the Oslo Sports Trauma Research Center Overuse Injury Questionnaire (OSTRC-O). Players who report a substantial injury in the OSTRC-O, i.e., not being able to participate in football activities, or have reduced their training volume or performance to a moderate or major degree, are contacted for full injury documentation. In addition to player data, academy coaches also complete a baseline questionnaire regarding coach experience and education.

Ethics and dissemination The study was approved by the Regional Ethical Review Authority at Karolinska Institutet, Stockholm, Sweden (2016/1251-31/4). All participating players and

For peer review only

their legal guardians give their written informed consent. The study will be reported in accordance with the Strengthening the Reporting of Observational studies in Epidemiology (STROBE). The results will be published in peer-reviewed academic journals and disseminated to the Swedish football movement through stakeholders and media.

Keywords: Acute injuries, bio-psychosocial factors, girls, gradual onset injuries, soccer, youth.

For peer review only

ARTICLE SUMMERY

Strengths and limitations of this study

- The study will increase the knowledge about risk factors, injuries, and health issues from different bio-psychosocial domains in young female football players.
- These results may be different from results in studies regarding male players and players in other age groups and can be a ground for specific injury prevention strategies.
- We aim to have a large sample size and to collect robust data of exposures, potential confounding factors and effect measure modifiers and the study design is previously used in a similar study KHAST, from our research group.
- Weekly self-reported data collection in adolescents might lead to misclassification of exposure and outcome.
- Using e-mails and SMS for weekly reports might decrease the response rates and thereby increase the risk of selection bias in the results.

INTRODUCTION

Four million females worldwide are registered football players, whereof 2.5 million are under 17 years old according to Fédération Internationale de Football Association (FIFA)[1]. Studies regarding injuries in female football are scarce compared to the number of studies in male football players[2-4]. In brief, these studies show that common injuries in female football players are joint and ligament injuries to the knee and ankle joints and muscle and tendon injuries of the thigh. In addition, there is a particular concern for concussions and anterior cruciate ligament (ACL) injuries in female players[3 5-8].

Female football players have more absence days from football due to injuries compared to male players[8], and long-term consequences of injuries might be considerable for young

For peer review only - http://bmjopen.bmj.com/site/about/guidelines.xhtml

football players[9]. For players with a history of injury, the risk of osteoarthritis in lower extremity joints are high and greater than in the general population[10 11]. Injuries may also lead to premature career ending[12], and mental health problems[13]. Identifying risk factors for injury is, therefore, an important step towards reduction of injury risk[14]. To identify possible risk factors well-designed prospective cohort studies are needed[15 16], and the suggested risk factors in this setting can be classified as bio-psychosocial factors (see Wiese-Bjornstal for bio-psychosocial view on a sport injury risk profile)[17]. Biological risk factors for injury in female players are previous injury[7 18-20], a hamstring/quadriceps ratio of less than 55 %, increased body mass index (BMI), as well as results of plyometric tests e.g., poor performance in drop jump landing test is associated with increased risk of ankle injury[21]. Other biological risk factors are young age[6 18], physical complaints at the beginning of the season, familiar disposition, i.e., a parent, sibling[18], or a twin[22] with knee injury also lower level of preseason aerobic fitness is associated with an increased risk of injuries during the season[23 24]. Results regarding joint hypermobility in female players as a risk factor are inconclusive in older studies[23 25], although in more resent published studies no relation was shown[26 27]. Risk factors for back pain in adolescents include rapid growth rate, and tight muscle imbalance[28], but risk factors for football related back/neck injuries in young females are not known. Psychological risk factors reported includes somatic trait anxiety, mistrust, and ineffective coping[29], life event stress[30], and perceived mastery climate[20]. Social factors that influenced the risk for injuries in female athletes are coaches' and player's education regarding injury prevention strategies[31], stress from teammates and coaches[20 29 32], and for back pain in adolescents; smoking[28]. In football, an identified situational specific risk factor is the playing positions defender and strikers[19].

In summary, there are inconsistent knowledge about risk factors for injuries in adolescent female football players, and lack of using a bio-psychosocial perspective in research. Hence,

For peer review only - http://bmjopen.bmj.com/site/about/guidelines.xhtml

the overall aim of the Karolinska football Injury Cohort study (KIC) is to identify risk factors for injuries in adolescent female football players from a bio-psychosocial perspective. Specific aims are to determine the incidence of injuries in young female football players and to identify modifiable risk factors for such injuries. Secondary aims include to describe changes in muscle strength and range of motion over a year, trajectories of pain, and to identify important factors for not being injured over a year.

METHODS AND ANALYSIS

This is a prospective observational cohort study designed in agreement with Strengthening the Reporting of Observational studies in Epidemiology (STROBE) guidelines[33].

Study setting and participants

Football clubs with adolescent female academy players aged 12 to 19 years, participating in Swedish divisions 1-2 for girls in the largest regions, are eligible to participate in the study. Clubs which meet the inclusion criteria are contacted and invited to the study and given oral and written information. Clubs which choose to take part in the study are provided with a more detailed oral and written information in the presence of players, legal guardians, and coaches.

A cohort of approximately 400 adolescent academy players will be recruited. An internal pilot study of 63 football players has been conducted to test the infra-structure and the implementation of the study, with satisfactory results (unpublished data).

Baseline measurements

Questionnaires

The baseline questionnaire covers potential risk factors for the aetiology of sport injuries as well as information about players' general health status. Players are surveyed in various areas

For peer review only - http://bmjopen.bmj.com/site/about/guidelines.xhtml

including a) *health*: health problems (e.g. illness), medication, age at menarche, amenorrhea, b) *lifestyle*: sleep patterns, eating habits, food supplements, tobacco as smoking or Swedish snus (snuff) and alcohol, c) *socioeconomic* factors: guardians' education, d) *football-related factors*: training and match play exposure, playing position, dominant limb, years of experience, other sports participation, injury preventive strategies (e.g. the Swedish injury prevention warm-up programme Knee Control)[34] (33), type of turf at the home facilities (artificial or natural grass) according to guidelines for football studies[35-37], e) *psychosocial factors*: modified General Health Questionnaire-12 (GHQ-12) consisting of 12 items regarding self-reported general psychological health using a four-point Likert scale[38], coping assessed by a 28 item self-report questionnaire that measure effective and ineffective strategies to cope with stressful events using a four-point Likert scale (Brief COPE)[39], player's passion to sport measured in harmonious and obsessive passions using a 14 item questionnaire with a seven-point Likert scale (Passion scale)[40], education in sport psychology, regularly seeing a sport psychologist/mental coach and perceived stress (single item question)[41], f) *previous injury history*: injuries occurring two- and three to six months prior to inclusion are captured using a modified Swedish version of the validated psychometric instrument Oslo Sports Trauma Research Center Overuse Injury Questionnaire (OSTRC-O)[42 43], and g) *back and neck pain:* low back pain (LBP) and upper back pain/neck pain (UBNP) frequency, intensity, disability and corresponding longitudinal trajectories the preceding 6-months using modified versions of The Chronic Pain Questionnaire (CPQ)[44] and Visual Trajectories Questionnaire- Pain (VTQ-P)[45], respectively.

In addition, coaches in the included teams are surveyed regarding their education, years of experience, the use of warm-up and stretching regime and implementation of injury prevention programmes.

Physical test protocol

For peer review only - http://bmjopen.bmj.com/site/about/guidelines.xhtml

The physical test protocol includes several tests that are considered valid, reliable, and field friendly; performed in approximately 60 minutes/player. The protocol comprises measurements of strength, mobility and control of lower extremity, trunk and neck and also include anthropometric measurements (height, weight and leg length) and are described briefly below and in more detail with visual presentations in the electronic supplementary file (Supplement 1).

All test procedures are conducted in indoors facilities during weekends. The physical tests are divided into nine test stations with 1-2 test leaders each (Supplement 1). Throughout the study, the total number of test leaders/pairs of test leaders who has performed each station ranges from 3-9. Hitherto, 52 clinically experienced test leaders have been involved in data collection. They were trained by MA, VL, NW and the previous test leader in charge of the station to ensure consistent execution and reliability. Information and instructions given to the players regarding the tests are standardized and test leaders refrain from coaching or encouraging the players in any way during the procedures.

A maximum of nine players are tested per session (i.e. one at each station) and are informed to train and compete as usual prior to testing. Players are informed to refrain from certain tests that evoke pain, provoke ongoing injuries or other health-related issues. Prior to performing the physical tests, players complete a standardized seven-minute warm-up programme comprising four minutes of jogging, 10 x 1 body weight squats, 10 x 1 body weight squat jumps, and 10 x 1 unilateral body weight lunges. Following the warm-up session, players are randomly assigned to a starting test station and subsequently follow a predefined order.

Calf heel raises

Ankle plantarflexion (PF) muscle endurance is investigated using unilateral weight bearing calf heel raises[46]. The player is instructed to perform maximum unilateral barefoot heel raises

For peer review only - http://bmjopen.bmj.com/site/about/guidelines.xhtml

continuously to failure, guided by a metronome to standardize the pace (1 second concentric-, 1 second eccentric contraction). The test leader registers the number of accomplished repetitions and discontinues when the player fails to reach the marked target height. The same procedure is then conducted on the opposite foot.

Active plantarflexion mobility

Active PF range of motion (ROM) is measured with a universal goniometer in supine position utilizing fibula and fifth metatarsal as reference marks[47 48]. The player is instructed to maintain extended knees throughout the movement, and to perform a sequence of six maximal active PF cycles from a neutral dorsiflexion (DF) position, whereof the finishing three trials are registered.

Weight bearing ankle dorsiflexion mobility

Weight bearing ankle DF ROM is measured in a lunge position with the player's foot placed upon a metric ruler 10 cm away from a wall[46 49]. The player is instructed to lunge forward, until contact with the wall is achieved without allowing the heel to lift off the ground. Three warm-up trials are performed from the 10 cm mark to familiarize the player with the test. Thereafter, the test leader measures the following three trials. From the 10 cm reference mark, the player progresses 1 cm away at a time from the wall until unable to perform a successful repetition. If unable to perform a successful repetition at the 10 cm reference mark, she is asked to progress 1 cm forward until able to complete a successful repetition. The maximal DF ROM is measured with a digital inclinometer (Clinometer, Plaincode, Stephanskirchen, Germany) and distance from the wall to the greater toe is measured in cm.

Trunk mobility

Trunk rotation mobility is measured in a modified seated rotation test, and a in a lunge position on a gym mat graded with 5 degrees increments[50-52]. The player is instructed to

For peer review only - http://bmjopen.bmj.com/site/about/guidelines.xhtml

maximally rotate alternating between right and left, in a cross-legged position and subsequently in a lunge position on the dominant, and non-dominant limb whilst the test leader measures the rotational degrees in the end range. Three repetitions are performed in each direction during the three separate positions, and the mean value for each position is later used for analysis.

Trunk strength

Isometric trunk rotational strength is measured in a modified standing wood chopper test utilizing a force gauge to evaluate force output (RS Pro Digital Force Gauge, RS Components Ltd., Corby, UK)[53-55]. In this modified test, the player holds a handle attached to the force gauge in shoulder height in a standing position. The player is instructed to generate force through her trunk and rotate for five seconds whilst maintaining straight arms. Three consecutive repetitions are conducted in each direction and the maximal force output is later used for analysis.

Deep neck flexor endurance

Deep neck flexor muscle endurance is assessed through a modified version of the Cranio-cervical flexion test (CCFT) with a pressure sensor (Stabilizer Pressure Bio-Feedback, Chattanooga Group Inc, Hixon, TN)[51 56 57]. The test consists of a pre-test and an endurance test. In the pre-test the player is positioned in a supine position on an examination table and are instructed to slightly push the neck against the pressure sensor to increase the pressure and then maintain the pressure for 3 × 3 seconds, with a 3 second rest in between each contraction, at a specific target pressure (TP), starting at 20 mmHg. If the player can perform this task, she is instructed to increase the pressure to 24 mmHg and keep the pressure for another 3 × 3 seconds. This is repeated with a 2-mmHg increase until the player reaches 30 mmHg. If the player can perform the pre-test the endurance test is subsequently performed. During the endurance test, the same setup and procedure as in the pre-test is carried out. However, the

For peer review only - http://bmjopen.bmj.com/site/about/guidelines.xhtml

player is instructed to hold each contraction at the TP for 3 x 10 with a 10 second rest in between contractions. The highest completed TP with a full set of 3 x 10 seconds contractions is later used for analysis.

Hip and knee strength

Isometric hip flexion, extension, adduction and abduction strength as well as eccentric hip abduction and adduction strength are measured with a hand-held dynamometer (HHD) (MicroFet2, Hoggan Health Industries inc. West Jordan, UT, USA)[58 59]. Furthermore, isometric knee extension strength is measured with a HHD and the player in a seated position with the knee joint in 90-degrees of flexion. Prior to executing the strength tests, two submaximal isometric contractions in each direction are performed to familiarize the player with the procedures. Three isometric contractions with gradually increasing power output for five seconds, and three maximally eccentric contractions for three seconds are performed in the isometric and eccentric tests, respectively, with a 10 second rest in between contractions. The maximal power output for each position is later used for analysis.

Hip mobility

Measures of passive hip ROM in flexion and abduction in prone position and extension, internal- and external rotation in supine position is obtained using a universal goniometer[60 61]. Three consecutive measurements for each position are performed for both the dominant and the non-dominant leg, and the mean value for each position is later used for analysis.

Functional performance tests

To assess the player's unilateral jump performance, the One-leg Long Box Jump Test (OLLBJ) and square hop test are performed[62 63]. A 40 x 40 cm square is marked on the foundation and later utilized as a reference mark in both tests.

In the OLLBJ, the starting position are calculated by dividing the player's height (cm) with

For peer review only - http://bmjopen.bmj.com/site/about/guidelines.xhtml

1.6 (height / 1.6). Thereafter, the player is instructed to stand on one leg on the starting position and then jump on one leg directed inside the boundaries of the square and maintain balance after landing. Three warm up trials and five consecutive test trials are performed on each leg. The total number of approved trials are registered by the test leader.

During the square hop test, previously described in detail[62 63], the player is instructed to jump on one leg in and out of the square as many times as possible for 15 seconds in a clockwise direction, timed with a stopwatch whilst the test leader registers the number of approved jumps. The player performs two warm up trials on each foot prior to executing the test.

Ankle and knee stability

To assess stability of player's talocrural joints, a modified anterior drawer test is employed[64 65]. Furthermore, a modified version of Fairbank's apprehension test is utilized to evaluate the player's stability in the patellofemoral joint[66]. The tests are conducted on both the dominant and non-dominant foot and knee and are considered positive if the player experience any pain or discomfort during the examination, and/or an involuntary contraction of the quadriceps musculature occur during the Fairbank's apprehension test.

Isometric back extensor endurance

Isometric back extensor endurance is assessed by the modified Sorensen test[67-69]. In this previously described modified test[67 68], the player's lower body are supported to an examination table in prone position with three straps and the anterior-superior iliac spine is aligned with the edge of the table. The player is instructed to keep her arms folded across the chest throughout the procedure and isometric maintaining the upper body in a horizontal position until failure whilst the test leader register the time elapsed. A digital inclinometer (Clinometer, Plaincode, Stephanskirchen, Germany) is placed upon a metric ruler at the level of th5 in the thoracic spine to monitor sagittal plane movement. Prior to the assessment, the

For peer review only - http://bmjopen.bmj.com/site/about/guidelines.xhtml

player completes a shorter warmup trial to orient the desired sagittal plane target angle.

Follow-up measurement and outcome

Follow-up measurements are collected prospectively during one year from the baseline. In the weekly online questionnaire, the players are asked to answer questions regarding new and ongoing injuries, LBP and UBNP intensity, social support, perceived stress, recovery, and to be able to consider workload, number of training and match play hours/week[70]. To assess whether players sustain football related injuries throughout the follow-up period, the Swedish version of OSTRC-O is employed and included in the weekly online questionnaire[42 43 71]. The OSTRC-O was modified by adding a question regarding absence/reduced participation in training/match due to reasons not related to injuries were added, as well as the option to specify injuries in different anatomical localizations in the lower- and upper extremity, back, neck, head and abdomen.

Football related injuries reported with the OSTRC-O in the weekly online questionnaire leading to moderate or severe reductions in participation/and or sports performance or complete inability to participate in sport are classified as a substantial injury in this study[42]. Players reporting new substantial injuries are contacted on telephone by a clinically experienced research assistant to answer a standardized interview with questions concerning the injury such as: injury mechanism, localisation, type, time-loss, re-injury, diagnosis, and medical care. Injuries are divided into acute and gradual onset. An acute injury is defined as a result from a specific, identifiable event, whereas injuries with gradual onset are defined as an injury without a single, identifiable event responsible for the injury[35]. Players receive an automated link to the online questionnaire sent by email each Sunday, with a reminder email the next day to players not answering. Furthermore, if no response is received, a text message reminder with the link is sent on Tuesdays. Finally, every other week representatives of the

For peer review only - http://bmjopen.bmj.com/site/about/guidelines.xhtml

study visits football clubs with players active in the study to collect unanswered surveys for the previous two-week period.

After 52 weeks of participation, a questionnaire with equivalent content as the baseline questionnaire (excluding OSTRC-O with 2- and 3-6-month recall) are distributed to the players to evaluate possible changes from the baseline characteristics. The first 106 included players also underwent a secondary physical test protocol after 52 weeks of follow-up. In the one-year follow-up questionnaire, different aspects of UBP and LBP, respectively, in the preceding six months are measured. "Have you had UBP/Have you had LBP" (Yes/No)? If yes, has the pain hindered your daily activities (No, Yes to some extent or Yes to a high degree)? If Yes, the "Visual Trajectories Questionnaire – Pain" is used to capture the longitudinal state of a player's pain experience of UBP and LBP and are retrospectively reported for the preceding six-month period[45]. See Table 1 for an overview of the measurements during the different phases of the study.

Table 1. Summary of the included measurements during the different phases of the study.

Phase	Measurements	Tests/tools
Baseline: players (consecutive during inclusion; 2016-ongoing)	Demographic information, general health status (history of pain, illness, medication, plagues, menstrual cycle, back and neck pain), lifestyle (sleep patterns, resilience, food supplements, use of tobacco or alcohol), stress, socioeconomic factors (guardians' education), football related factors (position, years of experience, injury preventive strategies.	KIC Baseline players, The Chronic Pain Questionnaire (CPQ)[44], Visual Trajectories Questionnaire-Pain (VTQ-P)[45],
	Anthropometric measurements (height, weight,	KIC test protocol

For peer review only - http://bmjopen.bmj.com/site/about/guidelines.xhtml

	leg length), and measurement of strength, mobility and control of lower extremity, trunk, and neck.	
	History of injury and complaints	Modified OSTRC-O[42 43]
	Passion	Passion scale[40]
	General Health	GHQ-12[38]
	Coping strategies	Brief COPE[39]
Baseline: coaches (consecutive during inclusion; 2016-ongoing)	Education, years of experience, the use of warm-up and stretching regime and implementation of injury prevention programs.	KIC Baseline coaches
Weekly follow-up: players (September 2016-ongoing)	Exposure to football training and match play	KIC weekly report
	Exposure to other physical activity.<br>Health (e.g. stress, recovery) and social support.	
	Report on pain, injury performance complaints.	Modified OSTRC-O[42 43]
In case of a substantial injury event	Report on injury/complaint (type of injury, localisation, inciting event)	KIC medical report
One-year follow-up: players (consecutive after 52 weeks participation: 2017-ongoing)	Football related factors (position, injury preventive strategies).<br>Health status (pain in back or neck) lifestyle (sleep patterns, resilience, food supplements, use of tobacco or alcohol, physical activity), stress, coping and passion for sport.	KIC One-year questionnaire

For peer review only

One-year follow-up (consecutive after 52 weeks participation in the first 106 included players)	Anthropometric measurements (height, weight, leg length), and measurement of strength, mobility and control of lower extremity, trunk, and neck.	KIC test protocol

Sample size

The statistical power for the analyses will depend on the exact research question, the number of exposed players and on if the exposure is continuous or categorized. The sample size in the KIC-project is based on the definition "a substantial injury" as proposed by Clarsen et al.,[42], and back injuries in adolescent female players in a previously published study[7]. Based on a relative risk of 1.9 for a substantial injury in the back/neck, when 88 of the players are exposed, and with a power of 0.80, a significance level 5 % and with potential 10% drop out and a follow-up time of one year to identify risk factors, 420 players will be included.

Statistical methods

The data in the KIC study will be used to answer several different research questions and accordingly different analyses methods and statistics will be used. Primary, Kaplan-Meier estimates will be used to describe incidence, and Cox regression analyses or discrete time survival analyses to measure the associations between exposure and outcome, and to adjust for confounding . Only players without substantial injuries the two preceding months (reported in the baseline questionnaire) will be considered in the risk analyses, and stratified analyses to examine effect measure modification will be performed when relevant. The development of injuries is likely complex and that is why we measure an extensive number of factors so that we can consider confounders, intermediators, and effect measure modifier in these analyses. When identifying trajectories of time varying factors Generalized Estimating

For peer review only - http://bmjopen.bmj.com/site/about/guidelines.xhtml

Equations will be used for these analyses to consider the covariance between repeated measurements.

Time plan

Approximate 400 players will be recruited from 2017 and followed weakly for one year from inclusion regarding injuries/complaints. Players will consecutively be invited and included from the year as the turn 13 years old and play in a participating club.

Data statement

The dataset and statistical codes will be available when the data collection is completed.

Patient and public involvement

No patient involved

ETHICS AND DISSEMINATION

The study was approved by the Regional Ethical Review Authority at Karolinska Institutet, Stockholm, Sweden (2016/1251-31/4). All participating players and their legal guardians receive written and oral information regarding the study and give their written informed consent when entering the study. Players under the age of 15 are required to have written informed consent from their legal guardians. The study will be performed in accordance with the recommendations guiding research involving human subjects adopted by the 18th World Medical Association General Assembly, Helsinki, Finland, June 1964, amended at the 64th World Medical Association General Assembly, Fortaleza, Brazil, October 2013. The study will be reported in accordance with the Strengthening the Reporting of Observational studies in Epidemiology (STROBE)[33]. The results will be presented in scientific conferences and

For peer review only - http://bmjopen.bmj.com/site/about/guidelines.xhtml

published in peer-reviewed academic journals as well as being disseminated to the Swedish football movement through stakeholders and media.

For peer review only

For peer review only - http://bmjopen.bmj.com/site/about/guidelines.xhtml

FOOTNOTES

Acknowledgements Martin Samuelsson, Lena Holm, and Henrik Källberg have been involved in planning of study design, Joakim Bogren, Victor Ramirez Kristiansen, contributed to the first version of the draft. Students from the classes DN 47, 48, 49, 50, 51, and 53 at the Scandinavian college of Naprapathic Manual Medicine, Stockholm, Sweden are participating during inclusion of participants, the physical tests, and the prospective data collection.

Author Contributions VL, ES, MA and UT initiated the study. All authors conceived the study and contributed to the development of the study protocol. ES is the study guarantor and submitted to ethic committee. UT and NW drafted the manuscript which was critically revised by all co-authors. The final manuscript was approved by all authors.

Funding This study is funded by grants from the Swedish Naprapathic, and the Norwegian Naprapathic Associations, Swedish Research Council for Sport Science, Active life foundation and Sophiahemmet foundation. There is no grant numbers for none of the funders (award/grant number: N/A).

Competing interests None declared.

Patient consent for publication Not required.

Provenance and peer review Not commissioned, externally peer reviewed.

E-mail addresses:

Ulrika.tranaeus@gih.se; Nathan.weiss@shh.se; Victorlyberg@hotmail.com; martin.hagglund@liu.se; markus.walden@telia.com; urban.johnson@hh.se; martin.asker@shh.se; eva.skillgate@shh.se

For peer review only

Twitter Martin Asker @martinasker, Martin Hägglund @MHgglund, Markus Waldén @MarkusWalden, Eva Skillgate @skillgate_eva, Nathan Weiss@nathnweiss

For peer review only

REFERENCES

1. Federation Internationale de Football Association F. Women's football survey. 2014. (accessed 2020-11-03).
2. López-Valenciano A, Ruiz-Pérez I, Garcia-Gómez A, et al. Epidemiology of injuries in professional football: a systematic review and meta-analysis. *Br J Sports Med* 2020;54(12):711-18. doi: 10.1136/bjsports-2018-099577 [published Online First: 2019/06/07]
3. Bennett P, Fawcett L. Trauma injuries sustained by female footballers. *Trauma* 2006;8(2):69-76. doi: 10.1177/1460408606072682
4. Hägglund M, Waldén M, Ekstrand J. Injuries among male and female elite football players. *Scand J Med Sci Sports* 2009;19(6):819-27.
5. Faude O, Junge A, Kindermann W, et al. Injuries in female soccer players - A prospective study in the German national league. *Am J Sport Med* 2005;33(11):1694-700. doi: 10.1177/0363546505275011
6. Le Gall F, Carling C, Reilly T. Injuries in young elite female soccer players. *Am J Sport Med* 2008;36(2):276-84. doi: 10.1177/0363546507307866
7. Clausen MB, Zebis MK, Moller M, et al. High Injury Incidence in Adolescent Female Soccer. *Am J Sport Med* 2014;42(10):2487-94. doi: 10.1177/0363546514541224
8. Larruskain J, Lekue JA, Diaz N, et al. A comparison of injuries in elite male and female football players: A five-season prospective study. *Scand J Med Sci Sports* 2018;28(1):237-45. doi: 10.1111/sms.12860
9. Lohmander LS, Ostenberg A, Englund M, et al. High prevalence of knee osteoarthritis, pain, and functional limitations in female soccer players twelve years after anterior cruciate ligament injury. *Arthritis Rheum* 2004;50(10):3145-52. doi: 10.1002/art.20589
10. Prien A, Prinz B, Dvorak J, et al. Health problems in former elite female football players: Prevalence and risk factors. *Scand J Med Sci Sports* 2017;27(11):1404-10. doi: 10.1111/sms.12747
11. Roos EM. Joint injury causes knee osteoarthritis in young adults. *Curr Opin Rheumatol* 2005;17(2):195-200.
12. Wylleman P, Reints A. A lifespan perspective on the career of talented and elite athletes: Perspectives on high-intensity sports. *Scand J Med Sci Sports* 2010;20:88-94. doi: 10.1111/j.1600-0838.2010.01194.x
13. Putukian M. The psychological response to injury in student athletes: a narrative review with a focus on mental health. *Brit J Sport Med* 2016;50(3):145-48. doi: 10.1136/bjsports-2015-095586
14. Van Mechelen W, Hlobil H, Kemper HC. Incidence, severity, aetiology and prevention of sports injuries. *Sports Med* 1992;14(2):82-99.
15. Dvorak J, Junge A, Chomiak J, et al. Risk factor analysis for injuries in football players - Possibilities for a prevention program. *Am J Sport Med* 2000;28(5):S69-S74.
16. Steffen K, Engebretsen L. More data needed on injury risk among young elite athletes. *Brit J Sport Med* 2010;44(7):485-89. doi: 10.1136/bjsm.2010.073833
17. Wiese-Bjornstal DM. Psychology and socioculture affect injury risk, response, and recovery in high-intensity athletes: a consensus statement. *Scand J Med Sci Sports* 2010;20(s2):103-11. doi: doi:10.1111/j.1600-0838.2010.01195.x
18. Hagglund M, Walden M. Risk factors for acute knee injury in female youth football. *Knee Surg Sport Tr A* 2016;24(3):737-46. doi: 10.1007/s00167-015-3922-z
19. Faude O, Junge A, Kindermann W, et al. Risk factors for injuries in elite female soccer players. *Brit J Sport Med* 2006;40(9):785-90. doi: 10.1136/bjsm.2006.027540

For peer review only - http://bmjopen.bmj.com/site/about/guidelines.xhtml

20. Steffen K, Pensgaard AM, Bahr R. Self-reported psychological characteristics as risk factors for injuries in female youth football. *Scand J Med Sci Sports* 2009;19(3):442-51. doi: 10.1111/j.1600-0838.2008.00797.x
21. Alahmad TA, Kearney P, Cahalan R. Injury in elite women's soccer: a systematic review. *Phys Sportsmed* 2020:1-7. doi: 10.1080/00913847.2020.1720548 [published Online First: 2020/02/07]
22. Magnusson K, Turkiewicz A, Hughes V, et al. High genetic contribution to anterior cruciate ligament rupture: Heritability ~69%. *Br J Sports Med* 2020;55(7):385-89. doi: 10.1136/bjsports-2020-102392
23. Ostenberg A, Roos H. Injury risk factors in female European football. A prospective study of 123 players during one season. *Scand J Med Sci Sports* 2000;10(5):279-85. doi: 10.1034/j.1600-0838.2000.010005279.x
24. Watson A, Brickson S, Brooks MA, et al. Preseason aerobic fitness predicts in-season injury and illness in female youth athletes. *Orthop J Sports Med* 2017;5(9) doi: 10.1177/2325967117726976
25. Soderman K, Alfredson H, Pietila T, et al. Risk factors for leg injuries in female soccer players: a prospective investigation during one out-door season. *Knee Surg Sport Tr A* 2001;9(5):313-21. doi: 10.1007/s001670100228
26. Nilstad A, Andersen TE, Bahr R, et al. Risk factors for lower extremity injuries in elite female soccer players. *Am J Sport Med* 2014;42(4):940-48. doi: 10.1177/0363546513518741
27. Blokland D, Thijs KM, Backx FJ, et al. No effect of generalized joint hypermobility on injury risk in elite female soccer players: A prospective cohort study. *Am J Sports Med* 2017;45(2):286-93. doi: 10.1177/0363546516676051
28. Feldman DE, Shrier I, Rossignol M, et al. Risk factors for the development of neck and upper limb pain in adolescents. *Spine* 2002;27(5):523-28.
29. Johnson U, Ivarsson A. Psychological predictors of sport injuries among junior soccer players. *Scand J Med Sci Sports* 2011;21(1):129-36. doi: 10.1111/j.1600-0838.2009.01057.x
30. Ivarsson A, Johnson U, Lindwall M, et al. Psychosocial stress as a predictor of injury in elite junior soccer: A latent growth curve analysis. *J Sci Med Sport* 2014;17(4):366-70. doi: 10.1016/j.jsams.2013.10.242
31. Junge A, Rosch D, Peterson L, et al. Prevention of soccer injuries: A prospective intervention study in youth amateur players. *Am J Sport Med* 2002;30(5):652-59.
32. Pensgaard AM, Ivarsson A, Nilstad A, et al. Psychosocial stress factors, including the relationship with the coach, and their influence on acute and overuse injury risk in elite female football players. *BMJ Open Sport Exerc Med* 2018;4(1):e000317. doi: 10.1136/bmjsem-2017-000317
33. von Elm E, Altman DG, Egger M, et al. The Strengthening the Reporting of Observational Studies in Epidemiology (STROBE) Statement: Guidelines for reporting observational studies. *Int J Surg* 2014;12(12):1495-99. doi: 10.1016/j.ijsu.2014.07.013
34. Walden M, Atroshi I, Magnusson H, et al. Prevention of acute knee injuries in adolescent female football players: cluster randomised controlled trial. *Bmj-British Medical Journal* 2012;344 doi: 10.1136/bmj.e3042
35. Fuller CW, Ekstrand J, Junge A, et al. Consensus statement on injury definitions and data collection procedures in studies of football (soccer) injuries. *Brit J Sport Med* 2006;40(3):193-201. doi: 10.1136/bjsm.2005.025270
36. Hägglund M, Waldén M, Bahr R, et al. Methods for epidemiological study of injuries to professional football players: developing the UEFA model. *Brit J Sport Med* 2005;39(6):340-46.
37. Emery CA, Meeuwisse WH, Hartmann SE. Evaluation of risk factors for injury in adolescent soccer: implementation and validation of an injury surveillance system. *Am J Sport Med* 2005;33(12):1882-91.

For peer review only - http://bmjopen.bmj.com/site/about/guidelines.xhtml

38. Hardy GE, Shapiro DA, Haynes CE, et al. Validation of the general health questionnaire-12 using a sample of employees from England's health care services. *Psychol Assess* 1999;11(2):159-65. doi: 10.1037/1040-3590.11.2.159
39. Carver CS. You want to measure coping but your protocol's too long: Consider the brief COPE. *Int J Behav Med* 1997;4(1):92-100. doi: 10.1207/s15327558ijbm0401_6
40. Vallerand RJ, Blanchard C, Mageau GA, et al. Les passions de l'Ame: On obsessive and harmonious passion. *J Pers Soc Psychol* 2003;85(4):756-67. doi: 10.1037/0022-3514-85.4.756
41. Salminen S, Kouvonen A, Koskinen A, et al. Is a single item stress measure independently associated with subsequent severe injury: a prospective cohort study of 16,385 forest industry employees. *Bmc Public Health* 2014;14(1):543.
42. Clarsen B, Myklebust G, Bahr R. Development and validation of a new method for the registration of overuse injuries in sports injury epidemiology: the Oslo Sports Trauma Research Centre (OSTRC) overuse injury questionnaire. *Br J Sports Med* 2013;47(8):495-502.
43. Ekman E, Frohm A, Ek P, et al. Swedish translation and validation of a web-based questionnaire for registration of overuse problems. *Scand J Med Sci* 2015;25(1):104-9. doi: 10.1111/sms.12157
44. Von Korff M, Ormel J, Keefe FJ, et al. Grading the severity of chronic pain. *Pain* 1992;50(2):133-49.
45. Dunn KM, Campbell P, Jordan KP. Validity of the visual trajectories questionnaire for pain. *J Pain* 2017;18(12):1451-58.
46. Dennis RJ, Finch CF, Elliott BC, et al. The reliability of musculoskeletal screening tests used in cricket. *Phys Ther Sport* 2008;9(1):25-33. doi: 10.1016/j.ptsp.2007.09.004
47. Youdas JW, Bogard CL, Suman VJ. Reliability of goniometric measurements and visual estimates of ankle joint active range of motion obtained in a clinical setting. *Arch Phys Med Rehab* 1993;74(10):1113-18. doi: 10.1016/0003-9993(93)90071-h
48. Ness BM, Sudhagoni RG, Tao H, et al. The reliability of a novel heel-rise test versus goniometry to assess plantarflexion range of motion. *Int J Sports Phys Ther* 2018;13(1):19-27. doi: 10.26603/ijspt20180019
49. Konor MM, Morton S, Eckerson JM, et al. Reliability of three measures of ankle dorsiflexion range of motion. *Int J Sports Phys Ther* 2012;7(3):279.
50. Frohm A, Heijne A, Kowalski J, et al. A nine-test screening battery for athletes: a reliability study. *Scand J Med Sci* 2012;22(3):306-15. doi: 10.1111/j.1600-0838.2010.01267.x
51. Asker M, Walden M, Kallberg H, et al. A prospective cohort study identifying risk factors for shoulder injuries in adolescent elite handball players: the Karolinska Handball Study (KHAST) study protocol. *BMC Musculoskelet Disord* 2017;18 doi: 10.1186/s12891-017-1852-2
52. Johnson KD, Kim KM, Yu BK, et al. Reliability of thoracic spine rotation range-of-motion measurements in healthy adults. *J Athl Training* 2012;47(1):52-60.
53. Andre MJ, Fry AC, Heyrman MA, et al. A reliable method for assessing rotational power. *J Strength Cond Res* 2012;26(3):720-24. doi: 10.1519/JSC.0b013e318227664d
54. Palmer TG, Uhl TL. interday reliability of peak muscular power outputs on an isotonic dynamometer and assessment of active trunk control using the chop and lift tests. *J Athl Training* 2011;46(2):150-59. doi: 10.4085/1062-6050-46.2.150
55. Zois J, P Sharp A, Talukdar K, et al. The reliability of a rotational power assessment of the core. *J Athl Enhanc* 2016;5(5)
56. Arumugam A, Mani R, Raja K. Interrater reliability of the craniocervical flexion test in asymptomatic individuals- A cross-sectional study. *J Manipulative Physiol Ther* 2011;34(4):247-53. doi: 10.1016/j.jmpt.2011.04.011
57. James G, Doe T. The craniocervical flexion test: intra-tester reliability in asymptomatic subjects. *Physiother Res Int* 2010;15(3):144-49.
58. Thorborg K, Bandholm T, Holmich P. Hip- and knee-strength assessments using a hand-held dynamometer with external belt-fixation are inter-tester reliable. *Knee Surg Sport Tr A* 2013;21(3):550-55. doi: 10.1007/s00167-012-2115-2

For peer review only - http://bmjopen.bmj.com/site/about/guidelines.xhtml

59. Kelln BM, McKeon PO, Gontkof LM, et al. Hand-held dynamometry: Reliability of lower extremity muscle testing in healthy, physically active, young adults. *J Sport Rehabil* 2008;17(2):160-70. doi: 10.1123/jsr.17.2.160
60. Prather H, Harris-Hayes M, Hunt DM, et al. Reliability and agreement of hip range of motion and provocative physical examination tests in asymptomatic volunteers. *PM R* 2010;2(10):888-95. doi: 10.1016/j.pmrj.2010.05.005
61. Nussbaumer S, Leunig M, Glatthorn JF, et al. Validity and test-retest reliability of manual goniometers for measuring passive hip range of motion in femoroacetabular impingement patients. *BMC Musculoskelet Disord* 2010;11 doi: 10.1186/1471-2474-11-194
62. Sharma N, Sharma A, Sandhu JS. Functional performance testing in athletes with functional ankle instability. *Asian J Sports Med* 2011;2(4):249.
63. Caffrey E, Docherty CL, Schrader J, et al. The ability of 4 single-limb hopping tests to detect functional performance deficits in individuals with functional ankle instability. *J Orthop Sports Phys Ther* 2009;39(11):799-806. doi: 10.2519/jospt.2009.3042
64. Vaseenon T, Gao Y, Phisitkul P. Comparison of two manual tests for ankle laxity due to rupture of the lateral ankle ligaments. *Iowa Orthop J* 2012;32:9-16.
65. de Vries JS, Kerkhoffs G, Blankevoort L, et al. Clinical evaluation of a dynamic test for lateral ankle ligament laxity. *Knee Surg Sport Tr A* 2010;18(5):628-33. doi: 10.1007/s00167-009-0978-7
66. Smith TO, Clark A, Neda S, et al. The intra- and inter-observer reliability of the physical examination methods used to assess patients with patellofemoral joint instability. *Knee* 2012;19(4):404-10. doi: 10.1016/j.knee.2011.06.002
67. Moreau CE, Green BN, Johnson CD, et al. Isometric back extension endurance tests: A review of the literature. *J Manipulative Physiol Ther* 2001;24(2):110-22. doi: 10.1067/mmt.2001.112563
68. Demoulin C, Vanderthommen M, Duysens C, et al. Spinal muscle evaluation using the Sorensen test: a critical appraisal of the literature. *Joint Bone Spine* 2006;73(1):43-50. doi: 10.1016/j.jbspin.2004.08.002
69. Latimer J, Maher CG, Refshauge K, et al. The reliability and validity of the Biering-Sorensen test in asymptomatic subjects and subjects reporting current or previous nonspecific low back pain. *Spine* 1999;24(20):2085-89. doi: 10.1097/00007632-199910150-00004
70. Malone S, Owen A, Newton M, et al. The acute:chonic workload ratio in relation to injury risk in professional soccer. *J Sci Med Sport* 2017;20(6):561-65. doi: 10.1016/j.jsams.2016.10.014
71. Nilstad A, Bahr R, Andersen TE. Text messaging as a new method for injury registration in sports: A methodological study in elite female football. *Scand J Med Sci Sports* 2014;24(1):243-49. doi: 10.1111/j.1600-0838.2012.01471.x

For peer review only - http://bmjopen.bmj.com/site/about/guidelines.xhtml

This is a supplementary document describing the included tests in Karolinska football Injury Cohort, KIC. Table S1 shows the test stations, number of test leaders and randomization of the tests. The persons in the images have given their consent that the images will be used in publications related to this study.

Calf heel raises

Ankle plantarflexion (PF) muscle endurance is investigated using unilateral barefoot weight bearing calf heel raises[46]. Firstly, the player's maximal weight being PF range of motion (ROM) is obtained by painting a reference mark on the player's heel at floor level and registering the maximal height achieved during one calf heel raise with a metric ruler.

The player is thereafter instructed to perform repeated maximum unilateral heel raises until failure, guided by a metronome to standardize the pace (1 second concentric-, 1 second eccentric contraction). The player is allowed to have light contact with her fingers against a wall. A repetition is considered approved on the basis whether knee extension is maintained, and the reference mark on the player's heel levels with the registered maximal PF ROM height on the ruler. The test leader registers the total number of approved repetitions and discontinues the test when the player fails to reach the marked maximal height. The same procedure is then conducted on the opposite foot. The order of execution is randomized prior to the test.

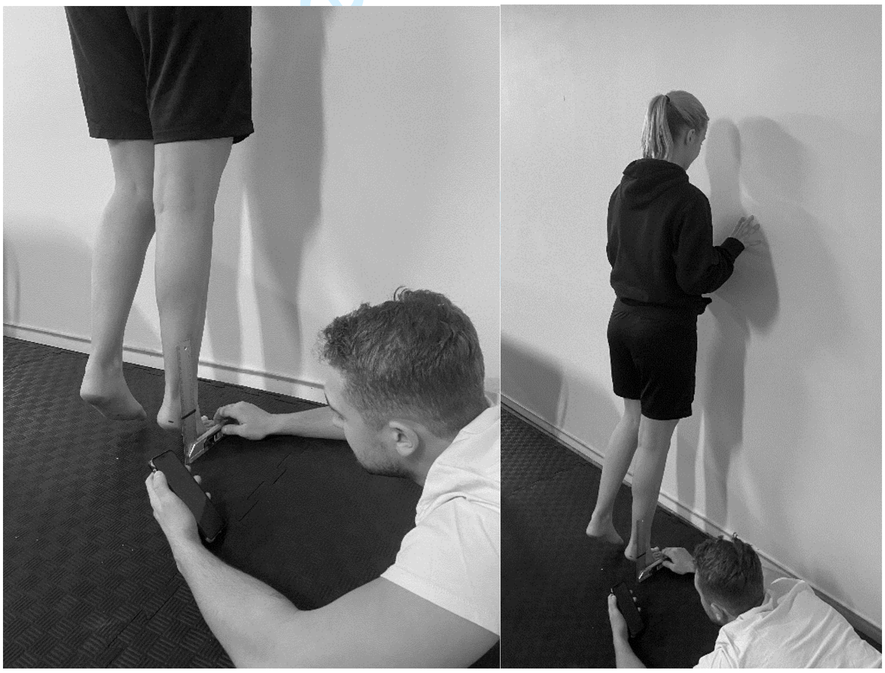

Figure S1. Calf heel raises.

Active plantarflexion mobility

Active PF ROM is measured with a clear plastic goniometer positioned at the lateral malleolus, utilizing fibula and fifth metatarsal as reference marks[47 48]. The player is positioned in supine on an treatment table, with feet off the edge of the table. The player is instructed to perform a sequence of six maximal active PF cycles starting from a neutral dorsiflexion (DF) position, whilst maintaining extended knees throughout the movement. The test leader measures and registers the maximal PF ROM in the final three cycles.

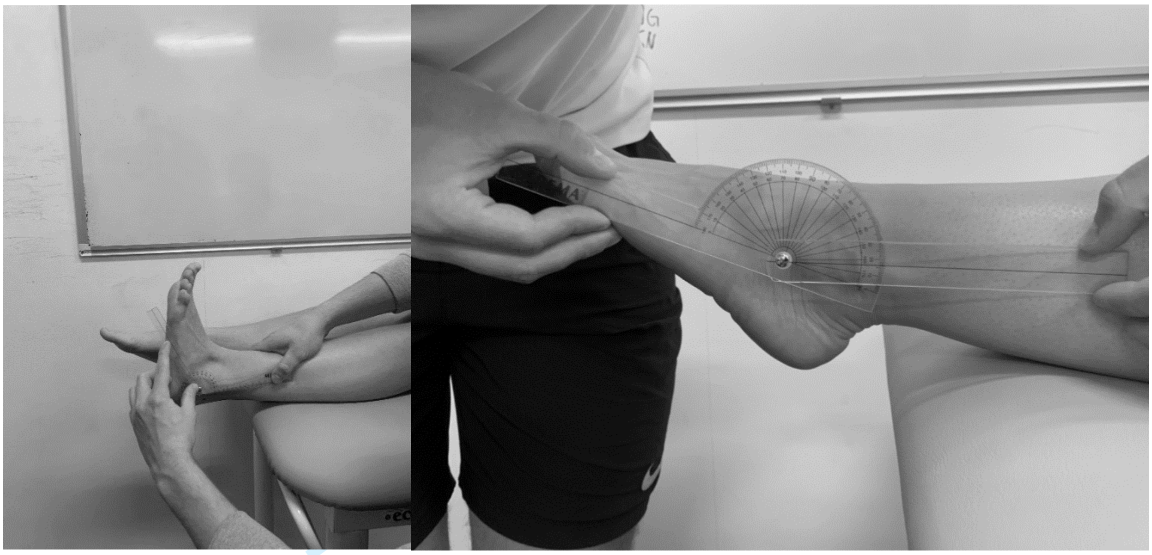

Figure S2. Active plantarflexion mobility execution. a) starting position in neutral dorsiflexion, b) end position in maximal active plantarflexion.

Weight bearing ankle dorsiflexion mobility

Weight bearing ankle DF ROM is measured in a standing lunge position with the player's foot placed upon a metric ruler 10 cm away from a wall to the player's greater toe[46 49]. The player is instructed to lunge forward, directing the knee in line with her second toe, until contact with the wall is achieved; without allowing the heel to lift off the ground, which is continuously monitored through the availability to maintain a piece of paper against the foundation. Throughout the test, the player is allowed to provide balance by light contact with her fingers against the wall.

Firstly, three consecutive warm-up trials are performed from the 10 cm mark to familiarize the player with the test. Thereafter, the test leader measures the following three trials. In each trial, the player begins from the reference mark (10 cm) and progresses 1 cm away from the wall at a time, until unable to perform a successful repetition. If the player is unable to perform an approved repetition at the 10 cm reference mark, she is asked to progress 1 cm forward until able to complete a successful repetition. Once the player achieves knee-wall contact, the DF ROM is measured with a digital inclinometer (Clinometer, Plaincode, Stephanskirchen, Germany) and the distance from the wall to the greater toe is measured in cm in the repetition furthest away from the wall in each trial.

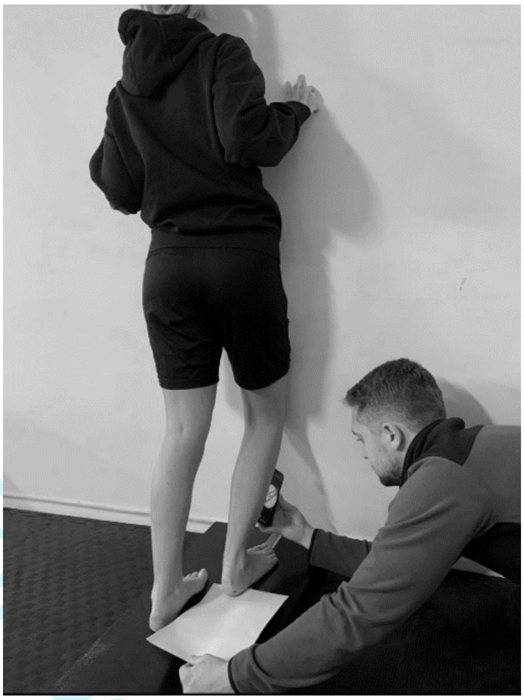

Figure S3. Weight bearing ankle dorsiflexion mobility.

Trunk mobility

Mobility in trunk rotation are measured in a cross-legged seated position, and in a lunge position with the player on a gym mat, graded with 5 degrees increments, from zero to one hundred and eighty degrees[50-52].

In the seated test (modified seated rotation test), the player is positioned at the center of the gym mat, in a cross-legged position with a wooden stick resting on the shoulders whilst keeping her arms crossed. If the player is unable to achieve the cross-legged sitting position, she is allowed to sit comfortable in an ordinary sitting position, which is noted by the test leader. Once in the starting position, the player is instructed to keep an upright posture and maximally rotate alternating between right and left for three times, whilst the test leader measures the rotational degrees in the end range.

The same procedure is thereafter repeated in a lunge position with the wooden stick resting on the player's shoulders. The player is positioned in a lunge position with her posterior knee at the center of the gym mat, and with her feet aligned on the zero-degree mark. Three consecutive maximal rotations are carried out alternating between right and left and is conducted in a lunge position for both the dominant, and non-dominant limb.

For peer review only

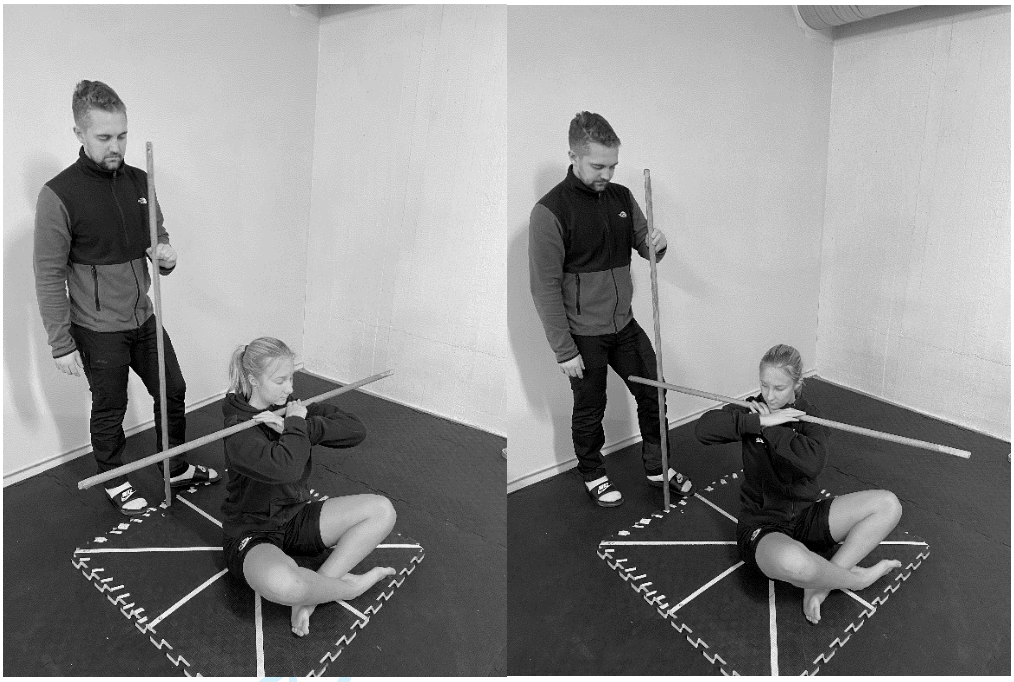

Figure S4. Modified seated rotation test. a) starting position, b) end position (right).

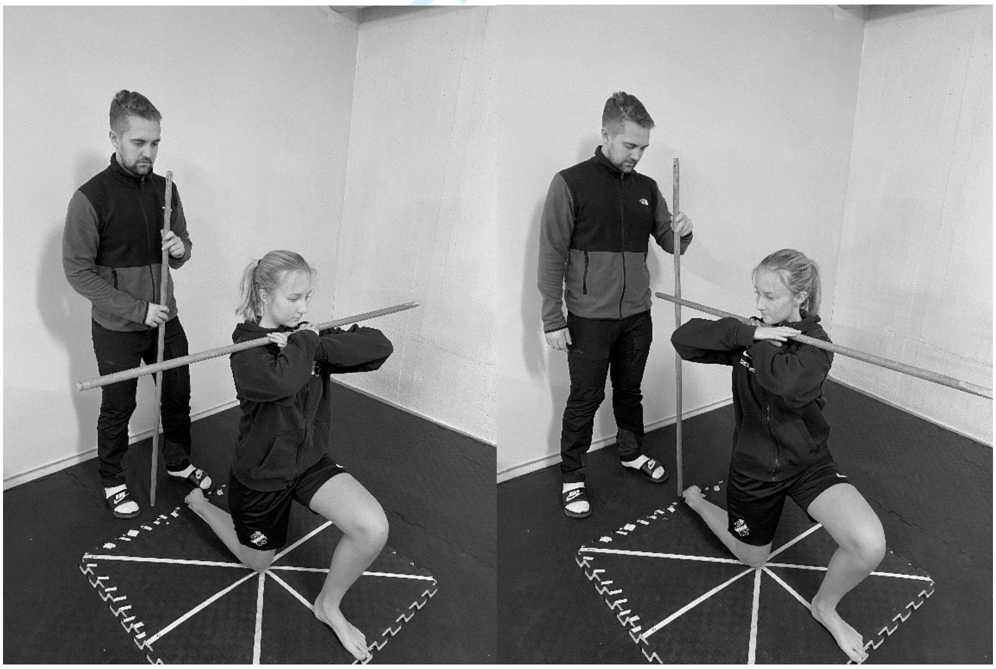

Figure S5. lunge rotation test. a) starting position left leg, b) end position (right).

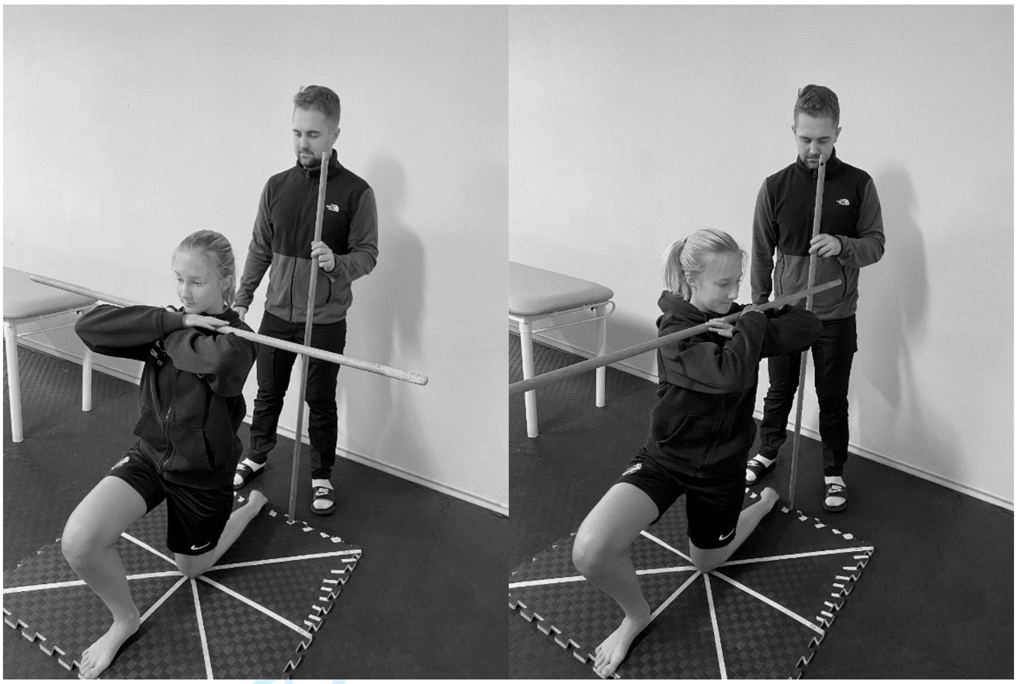

Figure S6. lunge rotation test. a) starting position right leg, b) end position (left).

Trunk strength

Isometric trunk rotational strength is measured in a modified standing wood chopper test utilizing a force gauge to evaluate force output (RS Pro Digital Force Gauge, RS Components Ltd., Corby, UK) [53-55].

In a standing position with extended arms, the player holds a handle in shoulder height, which is attached to the force gauge. The test leader positions the player in a 30-degree trunk rotation in the horizontal plane towards the anchor point (see figure S7).

The player is thereafter instructed to maximally generate force through her trunk and isometrically rotate in the opposite direction for five seconds whilst maintaining straight arms. Three consecutive repetitions are conducted for both right and left, and the maximal force output generated is used in the analyses. The order of execution is randomized prior to performing the test.

For peer review only

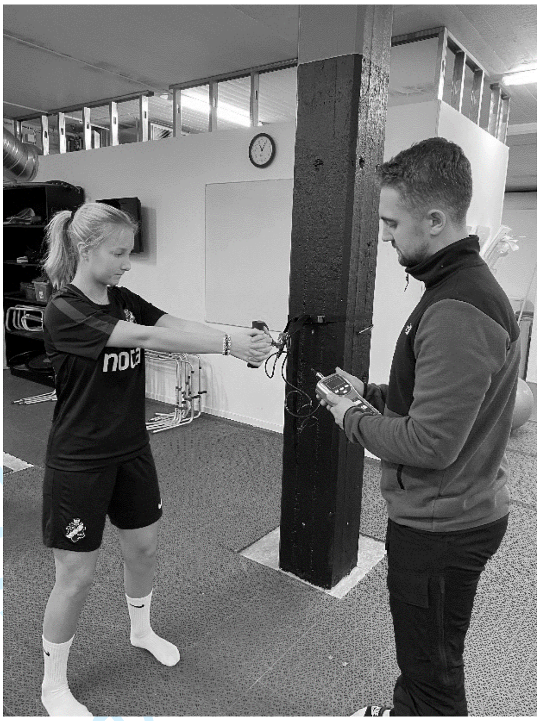

Figure S7. Modified standing wood chopper test (isometric rotation to the right).

Deep neck flexor endurance

Deep neck flexor muscle endurance is assessed through a modified version of the Cranio-cervical flexion test (CCFT) with a pressure sensor (Stabilizer Pressure Bio-Feedback, Chattanooga Group inc, Hixon, TN) [51 56 57].

Prior to executing the test, the player is instructed in how to perform a correct cranio-cervical flexion motion in standing and supine position through a gentle 'head nodding' cue. The player is positioned in supine position on a treatment table with her hands placed upon her abdomen or at the side of the body and with her feet on the table, with flexed hips and knees. With the player's head and neck in a neutral position, the pressure stabilizer is positioned sub-occipitally, and inflated to a baseline pressure of 20 mmHg. Firstly, a pre-test is conducted and later an endurance test.

During the pre-test, the player is instructed to perform a gentle cranio-cervical flexion to increase the pressure starting from a baseline of 20 mmHg with 2 mmHg increments to a maximum of 30 mmHg. 3x3 second contractions are carried out at each target pressure (TP) with a three second rest in between each contraction whilst the test leader monitors for potential compensational strategies: excessive use of global neck musculature, chin jerking, cervical spine retraction, jaw clenching, breath holding and a pressure loss of $\geq$ 2 mmHg. A stopwatch time the contractions and visual feedback of pressure level is provided by the test leader who holds the manometer dial so that both the player and the test leader can read it throughout the procedure.

The endurance test is conducted if the player completes each of the five TP (22, 24, 26, 28 and 30 mmHg) without exhibiting any of the compensational strategies and/or experiencing pain during the pre-test. During the endurance test, the same setup and procedure as in the

For peer review only

pre-test is carried out. The player is now instructed to hold each contraction at the TP for 3x10 seconds with a ten second rest in between contractions. The highest completed TP with a full set of 3x10 seconds contractions is registered by the test leader and later used for analysis.

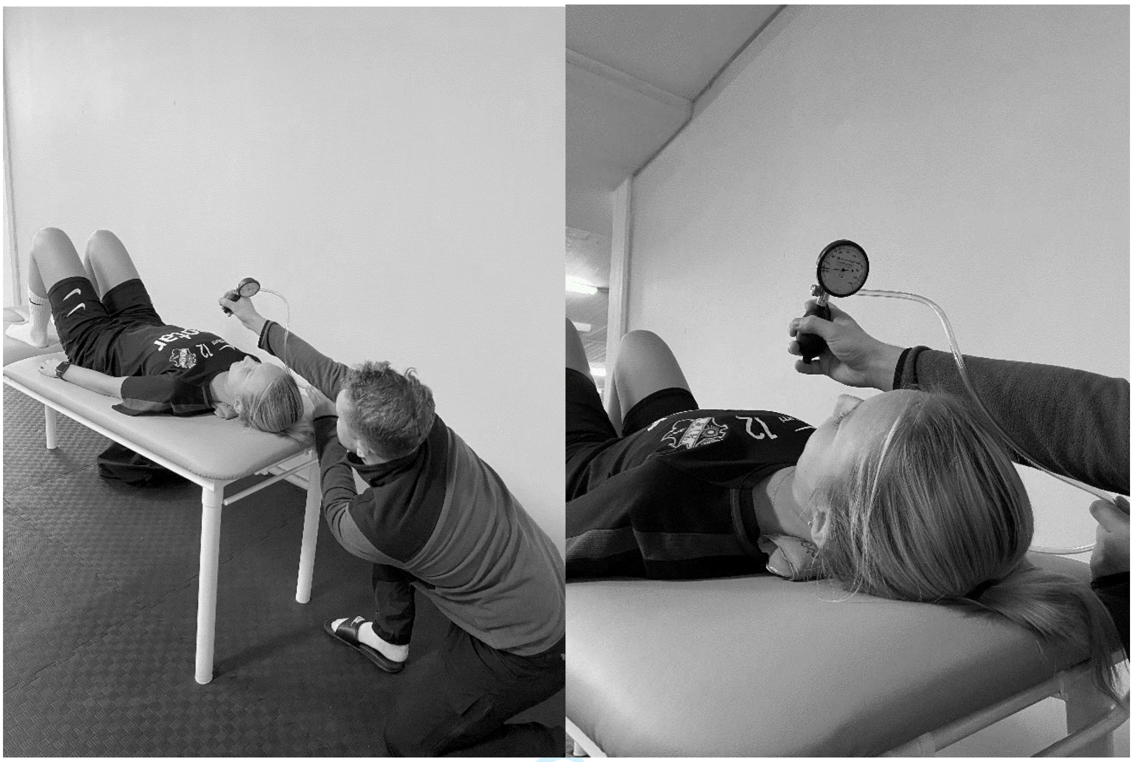

Figure S8. Modified cranio-cervical flexion test.

Hip- and knee strength

Isometric hip flexion, extension, adduction, and abduction strength as well as eccentric hip abduction and adduction and isometric knee extension strength are measured with a hand-held dynamometer (HHD) (MicroFet2, Hoggan Health Industries inc. West Jordan, UT, USA)[58 59].

Prior to executing the strength tests, two submaximal isometric contractions in each direction are performed to familiarize the player with the procedures. Three isometric contractions with gradually increasing force output for five seconds, and three maximal eccentric contractions for three seconds are performed in the isometric and eccentric tests, respectively, with a 10 second rest in between each contraction. The maximal force output for each position is registered by the test leader and later used for analysis. The order of execution and starting side is randomized prior to performing the tests at the particular test station (see table S1).

Isometric hip flexion strength

The player is positioned in a seated position at the edge of an treatment table, with 90-degrees of hip- and knee flexion. The HHD is positioned two centimeters proximal to the patella, and are externally fixated with a belt, which is secured under the leg of the treatment table, limiting hip flexion movement. The player is instructed to perform three isometric contractions on each leg.

For peer review only

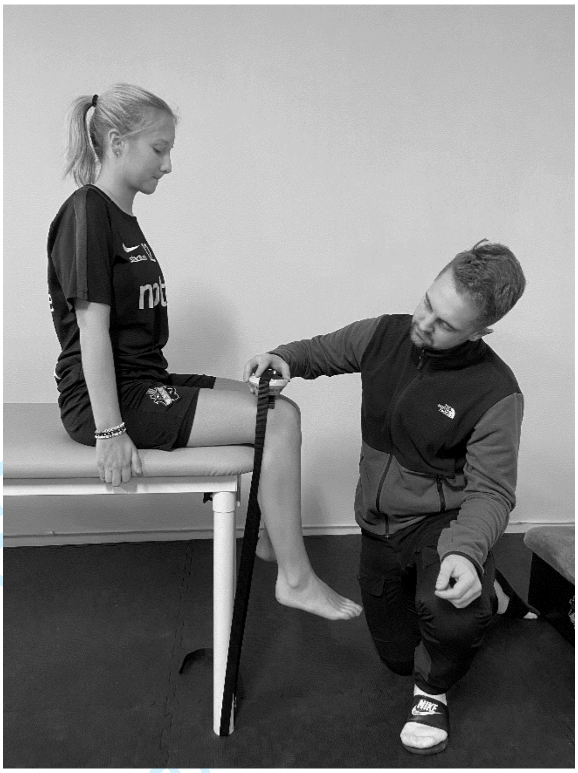

Figure S9. Isometric hip flexion strength (right hip).

Isometric knee extension strength

Seated in the same position as during the isometric hip flexion strength test, with a slightly extended knee joint, the HHD is positioned two centimeters proximal to the malleoli on the anterior aspects of the player's tibia and are externally fixated with a belt. The player is instructed to perform three isometric contractions on each leg respectively.

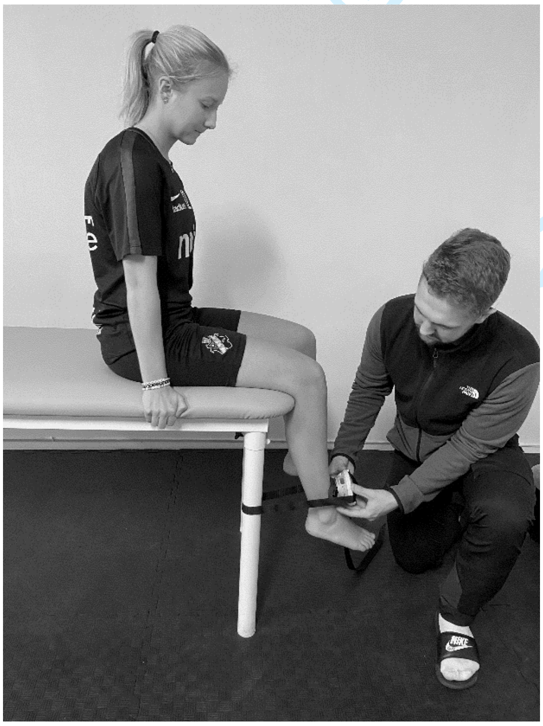

Figure S10. Isometric knee extension strength (right leg).

Isometric hip extension strength
With the player positioned in a prone position on a treatment table and with her feet off the edge of the table, the test leader externally fixates the HHD two centimeters proximal to the malleoli with a belt. Furthermore, the player is instructed to perform three maximal isometric contractions on each leg respectively

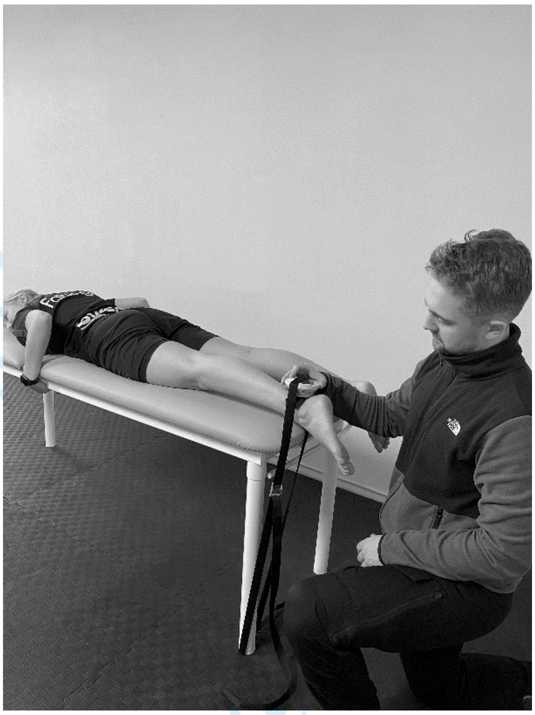

Figure S11. Isometric hip extension strength (left hip).

Isometric hip abduction strength
The player is positioned in a supine position on an treatment table, with the tested leg extended, and the non-tested leg flexed. The test leader positions and fixates the HHD two centimeters proximal to the lateral malleolus with a belt, which limits hip abduction movement. Thereafter, the player is instructed to perform three maximal isometric hip abductions, whilst the test leader measures the force output for both the left, and right side.

For peer review only

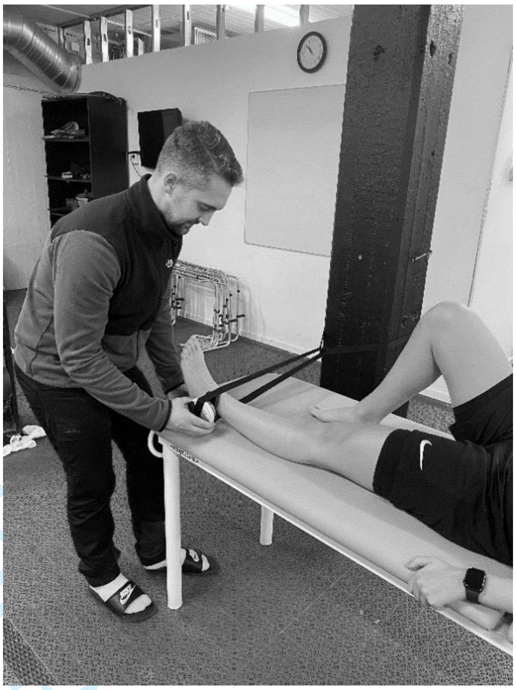

Figure S12. Isometric hip abduction strength (left hip).

Isometric hip adduction

Lying in the same position as during the isometric hip abduction the test leader places and fixates the HHD two centimeters proximal to the medial malleolus with a belt. Consequently, the player executes three maximal isometric hip adductions on the left and right side, whilst the test leader registers the force output.

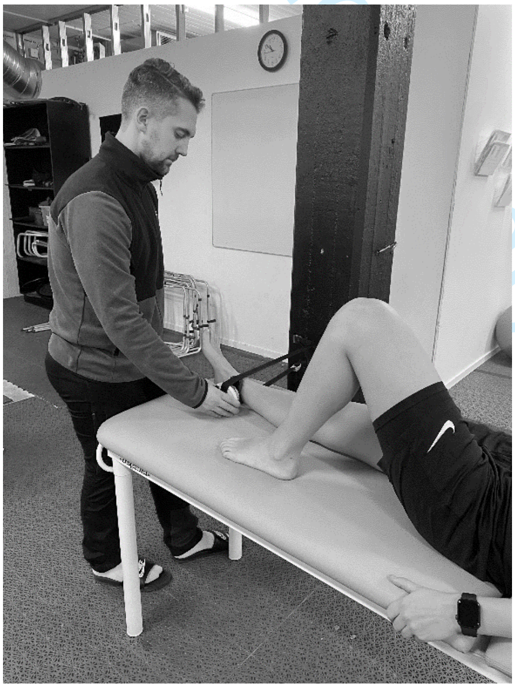

Figure S13. Isometric hip adduction strength (right hip).

For peer review only

Eccentric hip abduction strength

The player is in a side-lying position on an treatment table with the test leg extended, and the opposite leg flexed to 90 degrees in the knee- and hip joint, whilst a neutral hip position is maintained. The player is subsequently instructed to place the test leg in approximately 40 degrees of hip abduction, and the test leader places a HHD one centimeter proximally to the lateral malleolus. The test leader initiates the test by saying “push”, and when the player has built up a maximal isometric contraction, the test leader begins to apply a downward directed force with the HHD whilst the player resists eccentrically for five seconds. Three repetitions are carried out on both the right and leg left, and the maximal force output is later used for analysis.

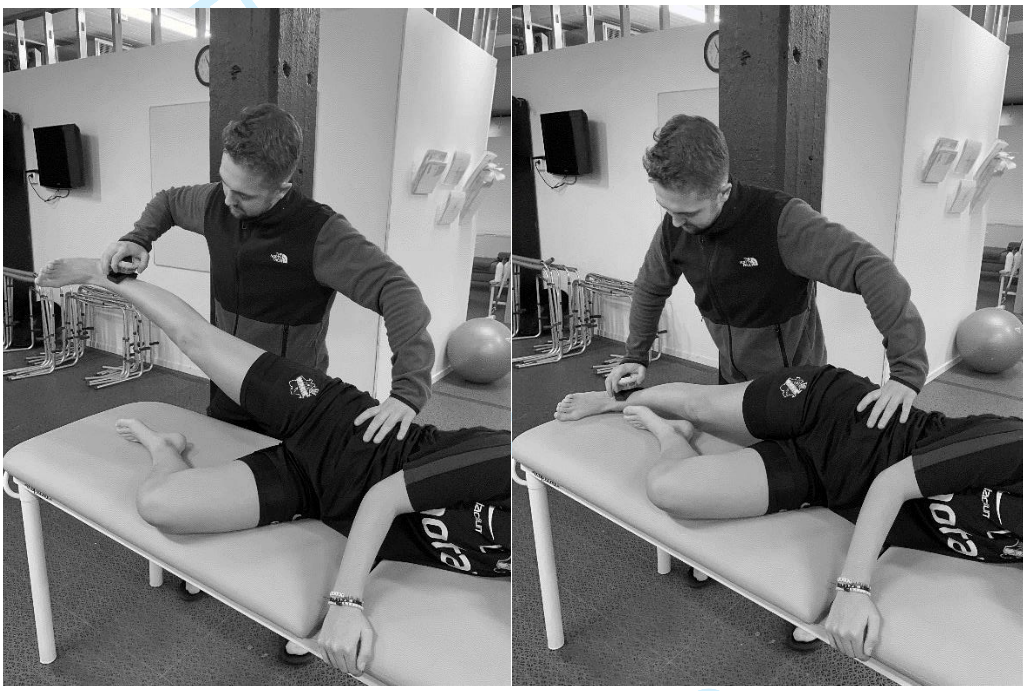

Figure S14. Eccentric hip abduction strength. a) starting position (right hip), b) end position (right hip).

Eccentric hip adduction strength

The player is positioned in the same manner as in the eccentric hip abduction strength test, with the tested leg extended, and the non-tested leg flexed in the hip- and knee joint. Thereafter, the player is instructed to place the test leg in a maximal adduction position, whereupon the test leader positions a HHD one centimeter proximally to the medial malleolus. The test is initiated when the test leaders says “push”, whereupon a downward directed force is applied with the HHD whilst the player resists eccentrically for five seconds. Three consecutive trials are conducted on both sides, and the test leader registers the force output.

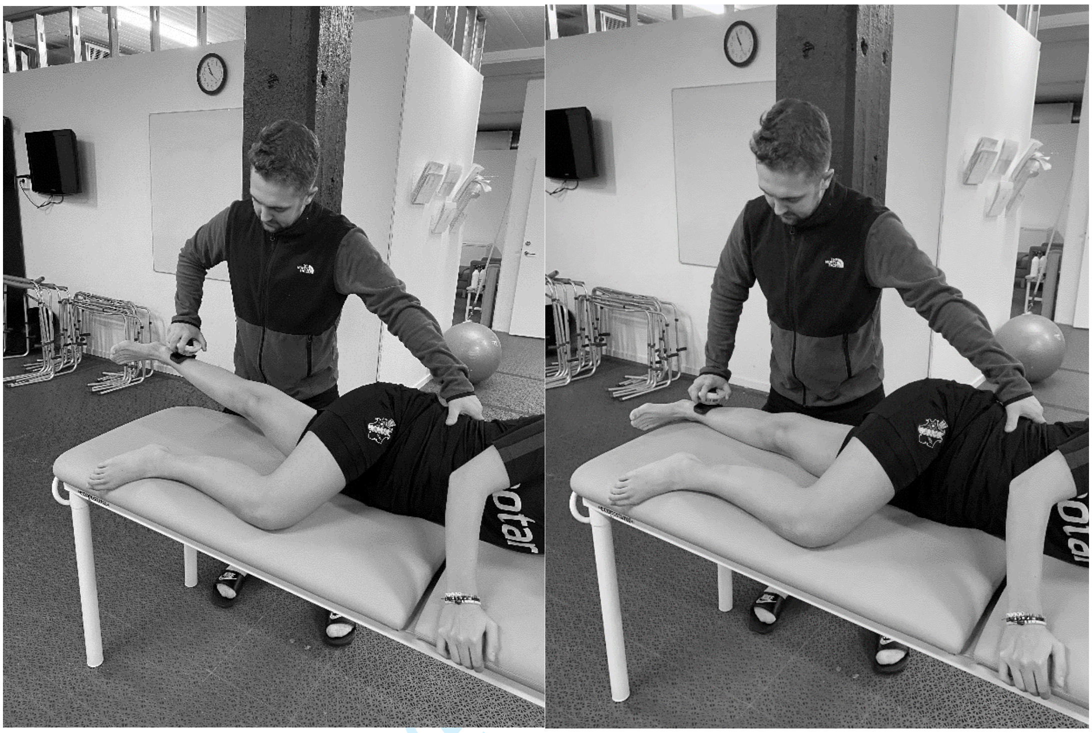

Figure S15. Eccentric hip abduction strength. a) starting position (left hip), b) end position (left hip).

Hip mobility

Measures of passive hip ROM in flexion, extension, abduction, internal- and external rotation are obtained using a universal clear plastic goniometer[60 61]. Three consecutive measurements for each position are performed for both the dominant and the non-dominant leg and the mean value for each position is later used for analysis. If the same value is obtained during the first and second measurement for a particular movement, a third one is not performed. The order of execution (side and movement) is randomized prior to performing the measures.

Passive hip flexion ROM

The player is positioned in supine position on a treatment table. With the player's leg held in a 90-degree knee flexion, test leader 1 moves the player's leg into a passive hip flexion until a firm end feel is achieved, and a posterior pelvic tilt occurs. Once the end feel is achieved, test leader 2 places the center of the goniometer at the greater trochanter and aligns one of the goniometer's arms with the player's femur, and the other one horizontally with the treatment table to read the goniometer. Three consecutive measures are conducted on each hip.

For peer review only

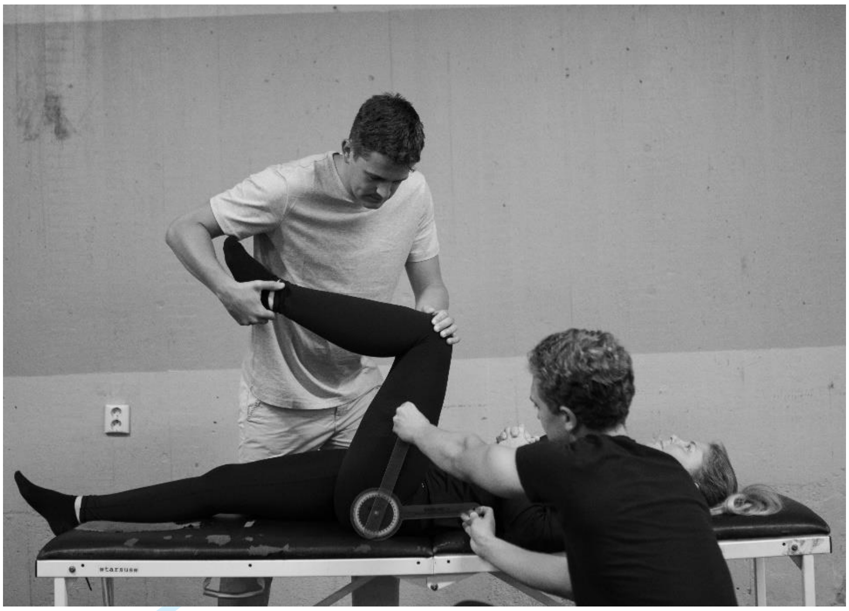

Figure S16. Passive hip flexion ROM (left hip).

Passive hip abduction ROM

The player is in a supine position on an treatment table with extended legs. While palpating the player's ipsilateral anterior superior iliac spine, test leader 1 holds the player's leg by the ankle and moves the leg into passive hip abduction until a firm end feel is achieved, and motion is felt at the pelvis. Thereafter, test leader 2 positions the goniometer at the player's hip, aligning the lever arms with the player's anterior superior iliac spine and femur, and reads the degrees of abduction. The test is repeated three times on each hip.

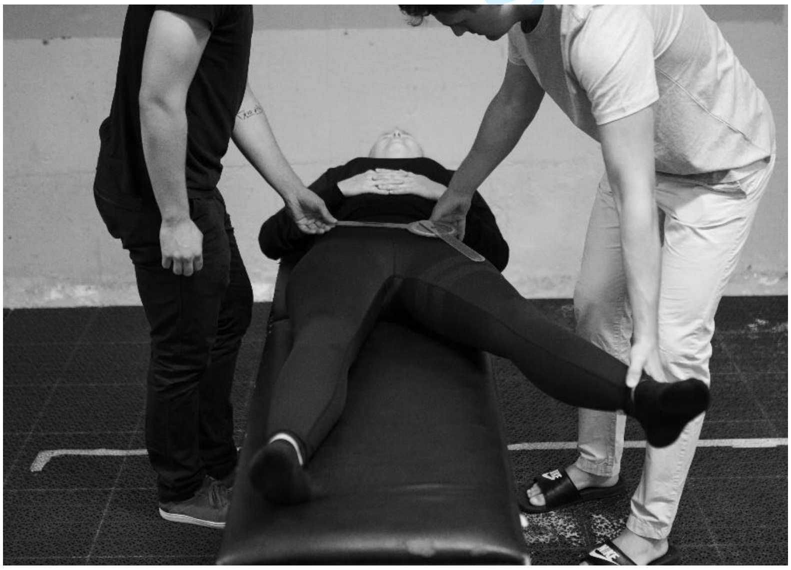

Figure S17. Passive hip abduction ROM (left hip).

Passive hip extension ROM

In prone position with extended legs, test leader 1 fixates the player's pelvis by placing a hand at the ipsilateral posterior superior iliac spine. Thereafter, while holding the player's leg at the knee, test leader 1 moves the player's leg into passive hip extension, until an end feel is achieved, indicated by an anterior tilt of the pelvis. Test leader 2 measures the degrees of passive hip flexion with the goniometer's center positioned at the greater trochanter, and the lever arms in line with the player's femur and the treatment table horizontally. Three measures are performed on each leg.

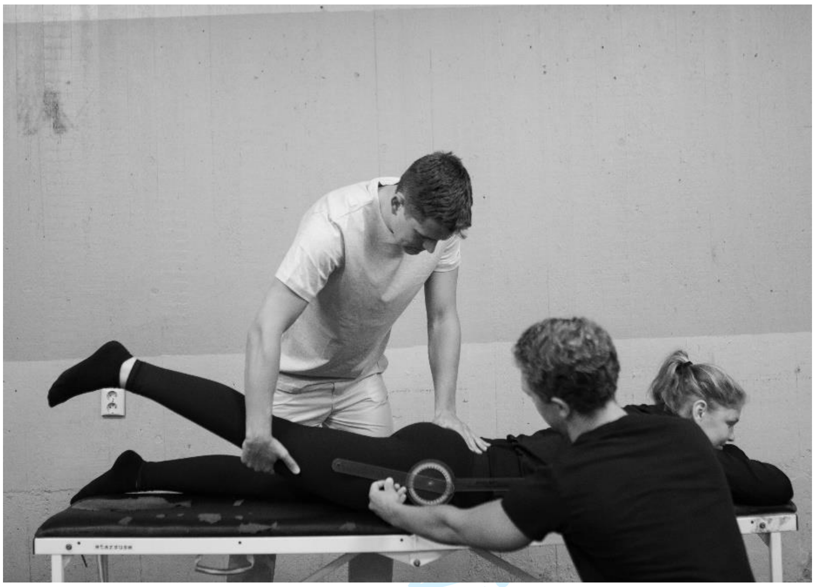

Figure S18. Passive hip extension ROM (right hip).

Passive hip internal- and external rotation ROM

In prone position, the player's leg is flexed to 90 degrees in the knee joint. Consequently, test leader 1 fixates the pelvis by placing his/her hand on the player's posterior superior iliac spine and performs a passive internal and external hip rotation, respectively, until an end feel is felt, indicated by an anterior pelvic tilt. Test leader 2 measures the degree of rotational mobility with a goniometer positioned at the knee, with the levers aligned with the player's tibia and with the treatment table horizontally. Three consecutive measures are conducted on each leg.

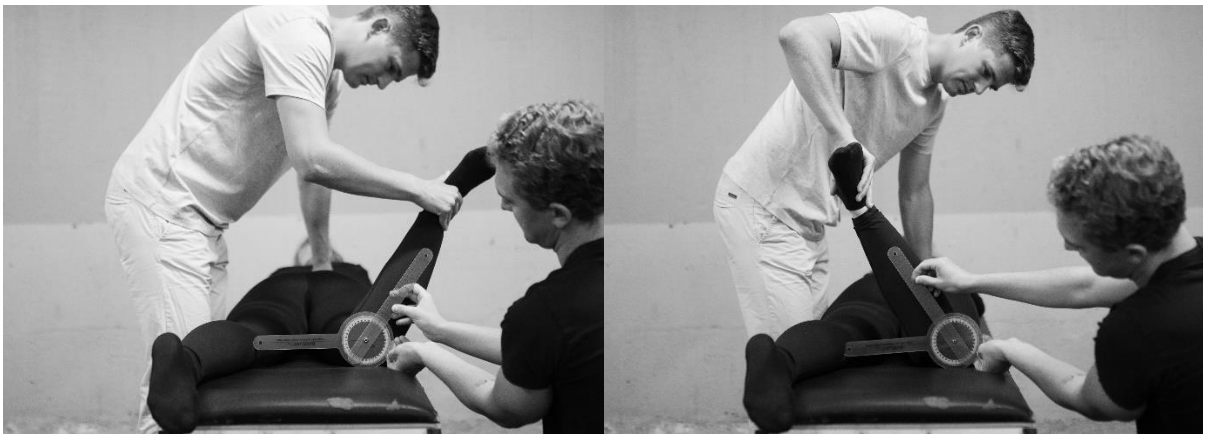

Figure S19. Passive hip rotational ROM (right hip). a) internal rotation, b) external rotation.

For peer review only

Jump performance tests

To assess the player's unilateral jump performance, the One-leg Long Box Jump Test (OLLBJ) and square hop test are performed[62 63]. A 40x40 cm square is marked on the foundation and later utilized as a reference mark in both tests. During the jump tests, players wear indoor sporting shoes.

One-leg long box jump test (OLLBJ)

Firstly, the starting position, i.e. the distance player's jump from to the 40x40 cm square is calculated by dividing the player's height in cm with 1.6 (height/1.6 = distance to the square). The player is instructed to stand on one leg at the starting position, and to perform a one-legged jump aiming inside the boundaries of the 40x40 cm square, and to maintain balance after landing. A trial is considered approved on the basis that the player land inside the 40x40 cm square, and adequately maintains balance after landing. The player performs three warm up trials on each leg, to familiarize with the procedure, and later five consecutive test trials. The test leader registers the total number of approved trials on each leg (0 to 5).

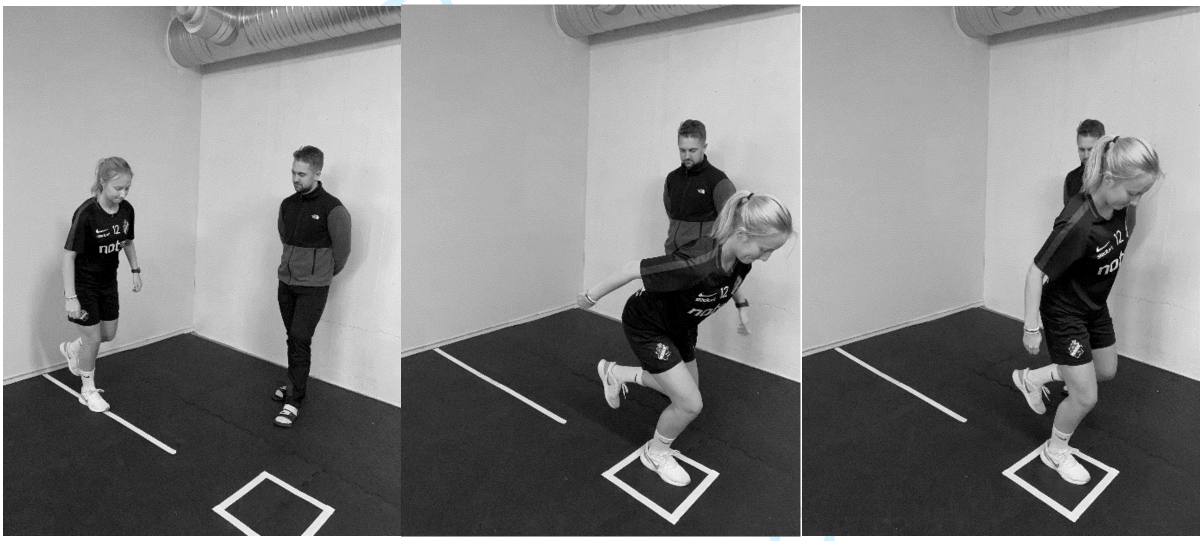

Figure S20. One-leg long box jump test (right leg). a) starting position, b) landing, c) balance maintained.

Square hop test

During the square hop test, the player is instructed to hop on one leg in and out of the 40x40 cm square as many times as possible for 15 seconds in a clockwise direction, timed with a stopwatch, whilst the test leader registers the number of approved hops. A hop is classified as approved on the basis whether the player begins a hop in the starting position (outside the square) and then executes the short hop task inside the square and then in the correct direction outside the square. Prior to the test, the player performs two warm up trials on each foot.

For peer review only

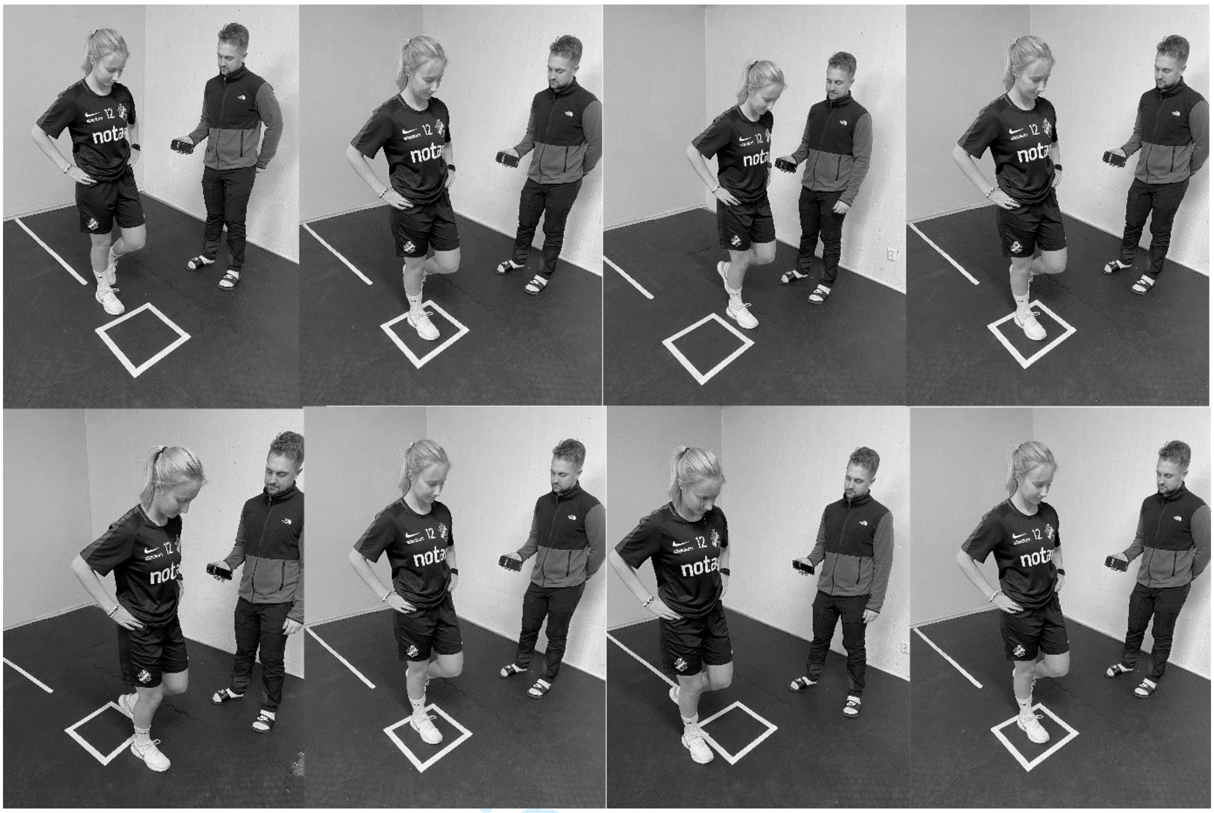

Figure S21. A series of square hop tests illustrated on the player's right leg.

Ankle- & knee stability

Modified anterior drawer test (ankle)

To assess talocrural stability or pain, a modified anterior drawer test is performed[64 65]. With the player in supine position with the test limb in knee flexion and the on the treatment table, the test leader applies an anteriorly directed force to the player's talus and a concurrent posteriorly directed force to the calcaneus. The test is conducted once on both the dominant and non-dominant foot and are considered positive if the player experiences any pain or discomfort during the procedure.

For peer review only

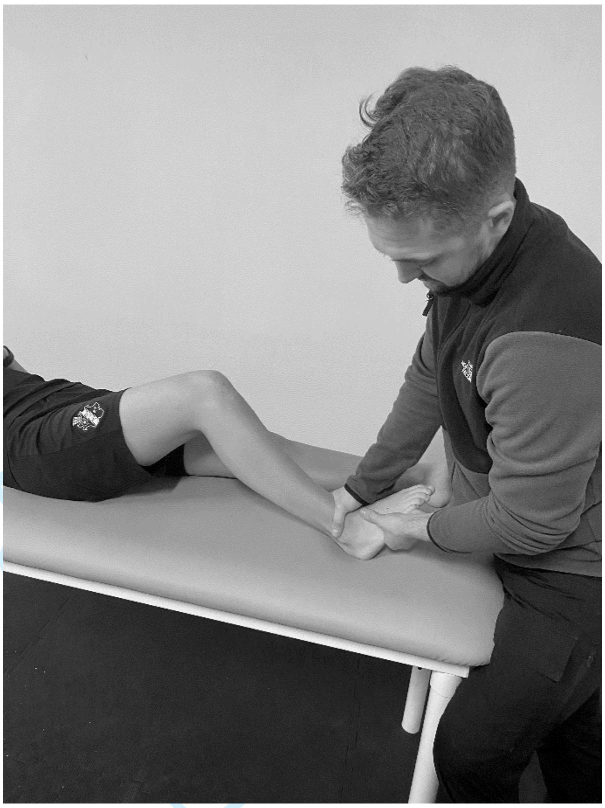

Figure S22. Modified anterior drawer test (right ankle).

Modified Fairbank's apprehension test (patellofemoral)

A modified version of Fairbank's apprehension test is conducted to evaluate stability or pain in the patellofemoral joint[66]. In supine position with extended legs, the test leader applies a laterally and subsequently medially directed force to the patella. The test is considered positive if the player experiences any pain or discomfort during the test, and/or an involuntary contraction of the quadriceps musculature. The test is carried out once on the player's dominant and non-dominant limb.

For peer review only

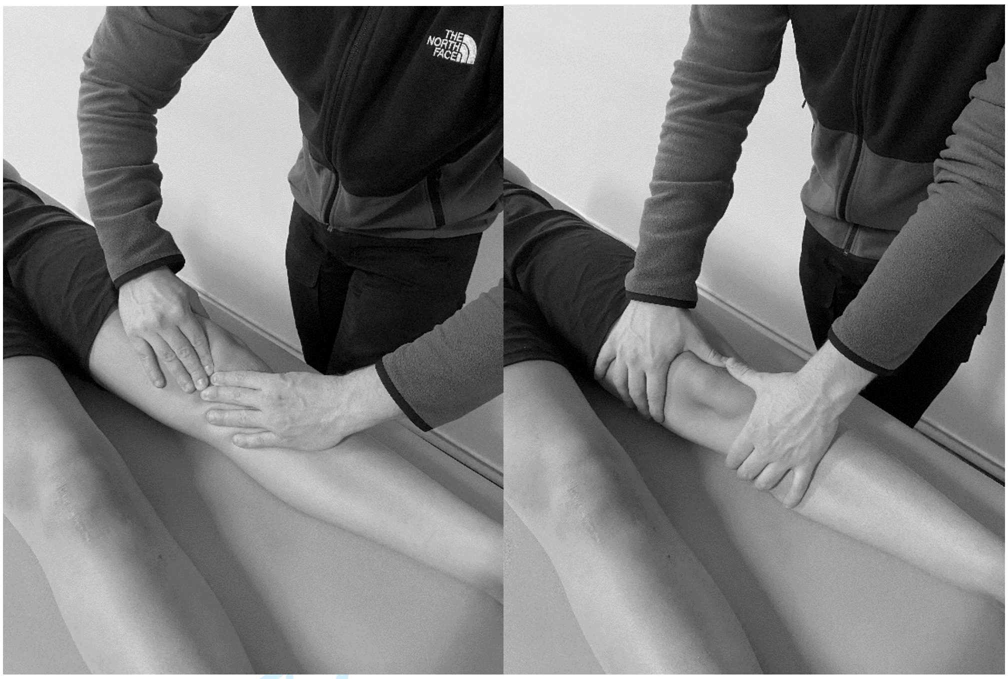

Figure S23. Modified Fairbank's apprehension test (left patellofemoral joint). a) lateral translation, b) medial translation.

Isometric back extensor endurance

Isometric back extensor endurance is assessed by a modified Sorensen test[67-69]. In prone position, the player's anterior superior iliac spine is positioned at the edge of the treatment table. The player´s lower body is supported to the treatment table with three straps positioned over the player's ankles, knees, and pelvis. Whilst the test leader fastens the player's lower body to the treatment table with the three straps, the player uses a box/stool for support.

The player is thereafter instructed to keep her arms folded across the chest and isometrically maintain the upper body in a horizontal position until failure whilst the test leader register the time elapsed. A digital inclinometer (Clinometer, Plaincode, Stephanskirchen, Germany) is placed upon a metric ruler at the level of the 5th vertebra of the thoracical spine to monitor sagittal plane movement. If the player's upper body deviate greater than 10 degrees in the sagittal plane on more than two occasions and/or experience pain during the procedure, the test is stopped. Prior to the test, the player completes a shorter warmup trial of 5 seconds to orient the desired sagittal plane target angle.

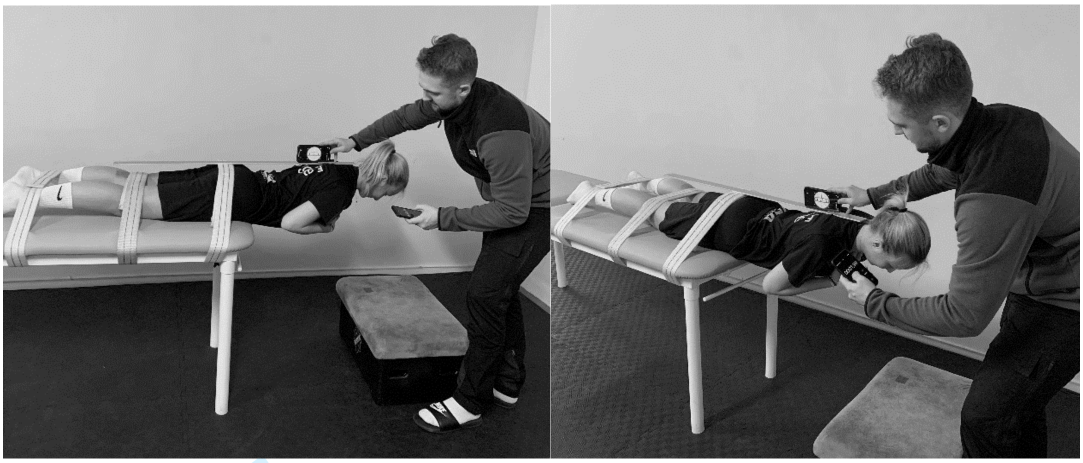

Figure S24. Modified Sorensen test.

Table S1. Test stations, number of test leaders and randomization of the physical test protocol.

Test	Test station	Number of test leaders	Randomized order of execution
Calf heel raises	1	1	Yes
Active plantarflexion mobility	2	1	No
Weight bearing ankle dorsiflexion mobility	2	1	No
Ankle- & Knee stability	2	1	No
Hip mobility	3	2	Yes
Isometric Knee extension, hip flexion & extension strength	4	2	Yes
Trunk mobility	5	1	No
Trunk strength	5	1	Yes
Isometric and eccentric hip abduction and adduction	6	2	Yes
Deep neck flexor endurance	7	1	n/a
Functional performance tests	8	1	Yes
Isometric back extensor endurance	9	1	n/a

n/a-not applicable

For peer review only

BMJ Open

Study protocol for a prospective cohort study identifying risk factors for sport injury in adolescent female football players: the Karolinska football Injury Cohort (KIC)

Journal:	*BMJ Open*
Manuscript ID	bmjopen-2021-055063.R1
Article Type:	Protocol
Date Submitted by the Author:	30-Nov-2021
Complete List of Authors:	Tranaeus, Ulrika; Karolinska Institutet; Swedish School of Sport and Health Sciences<br>Weiss, Nathan; Karolinska Institutet, Institute of Environmental Medicine; Sophiahemmet University, Department of health promotion science<br>Lyberg, Victor; Sophiahemmet University, Department of health promotion science<br>Hagglund, Martin; Linköping University, Department of Health, Medicine and Caring Sciences; Linköping University, Sport Without Injury ProgrammE (SWIPE)<br>Waldén, Markus; Linköping University, Sport Without Injury ProgrammE (SWIPE); Hässleholm-Kristianstad Hospitals, Department of Orthopaedics<br>Johnson, Urban; Halmstad University<br>Asker, Martin; Karolinska Institutet; Sophiahemmet University, Department of health promotion science<br>Skillgate, Eva; Karolinska Institutet; Sophiahemmet University, Department of health promotion science
**Primary Subject Heading**:	Sports and exercise medicine
Secondary Subject Heading:	Epidemiology
Keywords:	Orthopaedic sports trauma < ORTHOPAEDIC & TRAUMA SURGERY, SPORTS MEDICINE, EPIDEMIOLOGY

SCHOLARONE™ Manuscripts

Study protocol for a prospective cohort study identifying risk factors for sport injury in adolescent female football players: the Karolinska football Injury Cohort (KIC)

Ulrika Tranaeus,[1,2] (ORCID 0000-0002-2102-6352)

Nathan Weiss,[1,3] (ORCID 0000-0002-1814-020X)

Victor Lyberg,[3]

Martin Hägglund,[4,5,6] (ORCID 0000-0002-6883-1471)

Markus Walden,[5,6,7,8] (ORCID 0000-0002-6790-4042)

Urban Johnson,[9] (ORCID 0000-0003-0990-4842)

Martin Asker,[1,3,10] (ORCID 0000-0002-5387-3572)

Eva Skillgate[1,3] (ORCID 0000-0003-2096-1530)

[1] Institute of Environmental Medicine, Unit of Intervention and Implementation Research for Worker Health, Karolinska Institutet, Stockholm, Sweden

[2] The Swedish School of Sport and Health Sciences, Stockholm, Sweden

[3] Department of health promotion science, Musculoskeletal & Sports Injury Epidemiology Center, Sophiahemmet University, Stockholm, Sweden

[4] Unit of Physiotherapy, Department of Health, Medicine and Caring Sciences, Linköping University, Sweden

[5] Sport Without Injury ProgrammE (SWIPE), Department of Health, Medicine and Caring Sciences, Linköping University, Linköping, Sweden

[6] Football Research Group, Linköping, Sweden

[7] Unit of Community Medicine, Department of Health, Medicine and Caring Sciences, Linköping University, Sweden

[8] Department of Orthopaedics, Hässleholm-Kristianstad Hospitals, Hässleholm, Sweden

For peer review only - http://bmjopen.bmj.com/site/about/guidelines.xhtml

[9] Halmstad University, Halmstad Sweden

[10] Naprapathögskolan - Scandinavian College of Naprapathic Manual Medicine, Stockholm, Sweden

Corresponding author: Ulrika Tranaeus, Karolinska Institutet, IMM, SE 171 77, Stockholm, Sweden, +46 8 120 537 23, Ulrika.tranaeus@gih.se ulrika.tranaeus@ki.se

Word count: 3634

For peer review only

For peer review only - http://bmjopen.bmj.com/site/about/guidelines.xhtml

ABSTRACT

Introduction Football is a popular sport among young females worldwide, but studies concerning injuries in female players are scarce compared with male players. The aim of this study is to identify risk factors for injury in adolescent female football players.

Methods and analysis The Karolinska football Injury Cohort (KIC) is an ongoing longitudinal study that will include approximately 400 female football academy players 12-19 years old in Sweden. A detailed questionnaire regarding demographics, health status, lifestyle, stress, socioeconomic factors, psychosocial factors, and various football-related factors are completed at baseline and after one year. Clinical tests measuring strength, mobility, neuromuscular control of the lower extremity, trunk, and neck are carried out at baseline. Players are followed prospectively with weekly e-mails regarding exposure to football and other physical activity, health issues (such as stress, recovery, etc.), pain, performance, and injuries via the Oslo Sports Trauma Research Center Overuse Injury Questionnaire (OSTRC-O). Players who report a substantial injury in the OSTRC-O, i.e., not being able to participate in football activities, have reduced their training volume performance to a moderate or major degree, are contacted for full injury documentation. In addition to player data, academy coaches also complete a baseline questionnaire regarding coach experience and education.

Ethics and dissemination The study was approved by the Regional Ethical Review Authority at Karolinska Institutet, Stockholm, Sweden (2016/1251-31/4). All participating players and their legal guardians give their written informed consent. The study will be reported in accordance with the Strengthening the Reporting of Observational studies in Epidemiology (STROBE). The results will be published in peer-reviewed academic journals and disseminated to the Swedish football movement through stakeholders and media.

Keywords: Acute injuries, bio-psychosocial factors, girls, gradual onset injuries, soccer, youth.

For peer review only - http://bmjopen.bmj.com/site/about/guidelines.xhtml

ARTICLE SUMMARY

Strengths and limitations of this study

- A strength is the bio-psychosocial and multi professional perspective of the risk of injuries in young female football players and factors of importance for not being injured, even though the bio-psychosocial factors are not equal included
- Strengths are also the large sample size and the robust data collection of exposures, potential confounding factors, potential effect measure modifiers and outcome.
- A potential limitation is the risk of misclassification of time varying exposures and outcomes in the weekly self-reported data collection.
- Using e-mails and SMS for weekly reports might decrease the response rates and thereby increase the risk of selection bias in the results. If the response turns out to be low, there is a risk of selection bias in the risk analyses

For peer review only - http://bmjopen.bmj.com/site/about/guidelines.xhtml

INTRODUCTION

Four million females worldwide are registered football players, of which 2.5 million are under 17 years old according to Fédération Internationale de Football Association (FIFA)[1]. Studies regarding injuries in female football players are fewer compared to the number of studies in male football players[2-4]. In brief, these studies show that common injuries in female football players are joint and ligament injuries to the knee and ankle joints as well as muscle and tendon injuries of the thigh. In addition, there is a particular concern for concussions and anterior cruciate ligament (ACL) injuries in female players[3 5-8].

Female football players have more absence days from football due to injuries compared to male players[8], and long-term consequences of injuries might be considerable for young football players[9]. For players with a history of injury, the risk of osteoarthritis in lower extremity joints are high and greater than in the general population[10 11]. Injuries may also lead to premature career ending[12], and mental health problems[13]. Identifying risk factors for injury is, therefore, an important step towards reduction of injury risk[14]. To identify possible risk factors, well-designed prospective cohort studies are needed[15 16]. Specifically, the suggested risk factors in this setting can be classified as bio-psychosocial factors (see Wiese-Bjornstal for bio-psychosocial view on a sport injury risk profile)[17]. Biological risk factors for injury in female players are previous injury[7 18-20], a hamstring/quadriceps ratio of less than 55 %, increased body mass index (BMI), as well as results of plyometric tests e.g., poor performance in drop jump landing test is associated with increased risk of ankle injury[21]. Other biological risk factors associated with an increased risk of injury during the season are young age[6 18], physical complaints at the beginning of the season[18], familial disposition such as a parent/sibling[18], or a twin[22] with knee injury, and lower level of preseason aerobic fitness[23 24]. Findings regarding joint hypermobility as a risk factor in female players are inconclusive in older studies[23 25], although no association was shown in more recently

For peer review only - http://bmjopen.bmj.com/site/about/guidelines.xhtml

published studies[26 27]. Risk factors for back pain in adolescents include rapid growth rate, and tight muscle imbalance[28], but risk factors for football related back/neck injuries in young females are not known. Psychological risk factors reported includes somatic trait anxiety, mistrust, ineffective coping[29], life event stress[30], and perceived mastery climate[20]. Social factors that influenced the risk for injury in female athletes are coaches' and player's education regarding injury prevention strategies[31], stress from teammates and coaches[20 29 32], and for back pain in adolescents; smoking[28]. In football, an identified situational specific risk factor is the playing positions defender and strikers[19].

In summary, most knowledge about risk factors for injuries in adolescent female football players consists of isolated factors, and lack of using multidisciplinary and bio-psychosocial perspectives. Hence, the overall aim of the Karolinska football Injury Cohort study (KIC) is to identify risk factors for injuries in adolescent female football players from a bio-psychosocial perspective. Specific aims are to determine the incidence of injuries in young female football players and to identify modifiable risk factors for such injuries. Finally, our aims include to describe changes in muscle strength and range of motion over a year, trajectories of pain, and to identify important factors for not being injured over a year.

For peer review only - http://bmjopen.bmj.com/site/about/guidelines.xhtml

METHODS AND ANALYSIS

This is a prospective observational cohort study designed in agreement with Strengthening the Reporting of Observational studies in Epidemiology (STROBE) guidelines[33].

Study setting and participants

Football clubs with adolescent female academy players aged 12 to 19 years, participating in Swedish divisions 1-2 for girls in the largest regions, are eligible to participate in the study. Most players will be recruited in Stockholm. The district of Stockholm consists of 140 teams and approximate 2520 female players, 13-19 years old. Clubs which meet the inclusion criteria are contacted and invited to participate and are given oral and written information. Clubs which choose to take part in the study are provided with a more detailed oral and written information in the presence of players, legal guardians, and coaches.

A cohort of approximately 400 adolescent academy players will be recruited. An internal pilot study of 63 football players has been conducted to test the infra-structure and the implementation of the study, with satisfactory results (unpublished data).

Baseline measurements

Questionnaires

The baseline questionnaire covers potential risk factors for the aetiology of sport injuries as well as information about players' general health status. Players are surveyed in various areas, including *health*: health problems (e.g. illness), medication, age at menarche, amenorrhea, *lifestyle*: sleep patterns, eating habits, food supplements, tobacco as smoking or Swedish snus (snuff) and alcohol,and *socioeconomic* factors: guardians' education. *Included football-related factors* are: training and match play exposure, playing position, dominant limb, years of experience, other sports participation, injury preventive strategies (e.g. the Swedish injury prevention warmup programme Knee Control)[34], and type of turf at the home facilities

For peer review only - http://bmjopen.bmj.com/site/about/guidelines.xhtml

(artificial or natural grass) according to guidelines for football studies[35-37]. *Psychosocial factors* are surveyed using: the modified General Health Questionnaire-12 (GHQ-12) consisting of 12 items regarding self-reported general psychological health using a four-point Likert scale[38], coping assessed by a 28 item self-report questionnaire that measure effective and ineffective strategies to cope with stressful events using a four-point Likert scale (Brief COPE)[39], player's passion to sport measured in harmonious and obsessive passions using a 14 item questionnaire with a seven-point Likert scale (Passion scale)[40], education in sport psychology, regularly seeing a sport psychologist/mental coach and perceived stress (single item question)[41]. *Previous injury history*: injuries occurring within the previous six months prior to inclusion are captured using a modified Swedish version of the validated psychometric instrument Oslo Sports Trauma Research Center Overuse Injury Questionnaire (OSTRC-O)[42 43]. *Back and neck pain* is covering the frequency, intensity, disability of low back pain (LBP) and upper back pain/neck pain (UBNP) and corresponding longitudinal trajectories the preceding 6-months using modified versions of The Chronic Pain Questionnaire (CPQ)[44] and Visual Trajectories Questionnaire- Pain (VTQ-P)[45], respectively.

In addition, coaches in the included teams are surveyed regarding their education, years of experience, the use of warmup and stretching regime and implementation of injury prevention programmes.

Physical test protocol

The physical test protocol includes several tests that are considered valid, reliable, and field friendly; performed in approximately 60 minutes/player. The protocol comprises measurements of strength, mobility and control of lower extremity, trunk and neck and also include anthropometric measurements (height, weight and leg length). The protocol is briefly outlined below, however further details including visual presentations is available in the

For peer review only - http://bmjopen.bmj.com/site/about/guidelines.xhtml

electronic supplementary file (Supplement 1).

All test procedures are conducted in indoors facilities during weekends. The physical tests are divided into nine test stations with 1-2 test leaders each (Supplement 1). Hitherto, 52 clinically experienced test leaders have been involved in data collection. They were trained by MA, VL, NW and the previous test leader in charge of the station to ensure consistent execution and reliability. Information and instructions given to the players regarding the tests are standardised, and test leaders refrain from coaching or encouraging the players in any way during the procedures.

A maximum of nine players are tested per session (i.e. one at each station) and are informed to train and compete as usual prior to testing. Players are informed to refrain from certain tests that evoke pain, provoke ongoing injuries or other health-related issues. Prior to performing the physical tests, players complete a standardised seven-minute warm-up programme comprising four minutes of jogging, 10 x 1 body weight squats, 10 x 1 body weight squat jumps, and 10 x 1 unilateral body weight lunges. Following the warm-up session, players are randomly assigned to a starting test station and subsequently follow a predefined order.

Calf heel raises

Ankle plantarflexion (PF) muscle endurance is investigated using unilateral weight bearing calf heel raises[46]. The player is instructed to perform maximum unilateral barefoot heel raises continuously to failure, guided by a metronome to standardise the pace (1 second concentric-, 1 second eccentric contraction). The test leader registers the number of accomplished repetitions and discontinues when the player fails to reach the marked target height. The same procedure is then conducted on the opposite foot.

Active plantarflexion mobility

Active PF range of motion (ROM) is measured with a universal goniometer in supine position

For peer review only - http://bmjopen.bmj.com/site/about/guidelines.xhtml

utilizing fibula and fifth metatarsal as reference marks[47 48]. The player is instructed to maintain extended knees throughout the movement, and to perform a sequence of six maximal active PF cycles from a neutral dorsiflexion (DF) position, of which the final three trials are registered.

Weight bearing ankle dorsiflexion mobility

Weight bearing ankle DF ROM is measured in a lunge position with the player's foot placed upon a metric ruler 10 cm away from a wall[46 49]. The player is instructed to lunge forward, until contact with the wall is achieved without allowing the heel to lift off the ground. Three warm-up trials are performed from the 10 cm mark to familiarize the player with the test. Thereafter, the test leader measures the following three trials. From the 10 cm reference mark, the player progresses 1 cm away at a time from the wall until unable to perform a successful repetition. If unable to perform a successful repetition at the 10 cm reference mark, she is asked to progress 1 cm forward until able to complete a successful repetition. The maximal DF ROM is measured with a digital inclinometer (Clinometer, Plaincode, Stephanskirchen, Germany) and distance from the wall to the greater toe is measured in cm.

Trunk mobility

Trunk rotation mobility is measured in a modified seated rotation test, and a in a lunge position on a gym mat graded with 5 degrees increments[50-52]. The player is instructed to maximally rotate alternating between right and left, in a cross-legged position and subsequently in a lunge position on the dominant, and non-dominant limb whilst the test leader measures the rotational degrees in the end range. Three repetitions are performed in each direction during the three separate positions, and the mean value for each position is later used for analysis.

Trunk strength

For peer review only

For peer review only - http://bmjopen.bmj.com/site/about/guidelines.xhtml

Isometric trunk rotational strength is measured in a modified standing wood chopper test utilising a force gauge to evaluate force output (RS Pro Digital Force Gauge, RS Components Ltd., Corby, UK)[53-55]. In this modified test, the player holds a handle attached to the force gauge in shoulder height in a standing position. The player is instructed to generate force through her trunk and rotate for five seconds whilst maintaining straight arms. Three consecutive repetitions are conducted in each direction and the maximal force output is later used for analysis.

Deep neck flexor endurance

Deep neck flexor muscle endurance is assessed through a modified version of the Cranio-cervical flexion test (CCFT) with a pressure sensor (Stabilizer Pressure Bio-Feedback, Chattanooga Group Inc, Hixon, TN)[51 56 57]. The test consists of a pre-test and an endurance test. In the pre-test the player is positioned in a supine position on an examination table and are instructed to slightly push the neck against the pressure sensor to increase the pressure and then maintain the pressure for 3 × 3 seconds, with a 3 second rest in between each contraction, at a specific target pressure (TP), starting at 20 mmHg. If the player can perform this task, she is instructed to increase the pressure to 24 mmHg and keep the pressure for another 3 × 3 seconds. This is repeated with a 2-mmHg increase until the player reaches 30 mmHg. If the player can perform the pre-test the endurance test is subsequently performed. During the endurance test, the same setup and procedure as in the pre-test is carried out. However, the player is instructed to hold each contraction at the TP for 3 x 10 with a 10 second rest in between contractions. The highest completed TP with a full set of 3 x 10 seconds contractions is later used for analysis.

Hip and knee strength

Isometric hip flexion, extension, adduction and abduction strength as well as eccentric hip abduction and adduction strength are measured with a hand-held dynamometer (HHD)

For peer review only - http://bmjopen.bmj.com/site/about/guidelines.xhtml

(MicroFet2, Hoggan Health Industries inc. West Jordan, UT, USA)[58 59]. Furthermore, isometric knee extension strength is measured with a HHD and the player in a seated position with the knee joint in 90-degrees of flexion. Prior to executing the strength tests, two submaximal isometric contractions in each direction are performed to familiarize the player with the procedures. Three isometric contractions with gradually increasing power output for five seconds, and three maximally eccentric contractions for three seconds are performed in the isometric and eccentric tests, respectively, with a 10 second rest in between contractions. The maximal power output for each position is later used for analysis.

Hip mobility

Measures of passive hip ROM in flexion and abduction in prone position and extension, internal- and external rotation in supine position is obtained using a universal goniometer[60 61]. Three consecutive measurements for each position are performed for both the dominant and the non-dominant leg, and the mean value for each position is later used for analysis.

Functional performance tests

To assess the player's unilateral jump performance, the One-leg Long Box Jump Test (OLLBJ) and square hop test are performed[62 63]. A 40 x 40 cm square is marked on the foundation and later utilised as a reference mark in both tests.

In the OLLBJ, the starting position are calculated by dividing the player's height (cm) with 1.6 (height / 1.6). Thereafter, the player is instructed to stand on one leg on the starting position and then jump on one leg directed inside the boundaries of the square and maintain balance after landing. Three warm up trials and five consecutive test trials are performed on each leg. The total number of approved trials are registered by the test leader.

During the square hop test, previously described in detail[62 63], the player is instructed to jump on one leg in and out of the square as many times as possible for 15 seconds in a clockwise

For peer review only

For peer review only - http://bmjopen.bmj.com/site/about/guidelines.xhtml

direction, timed with a stopwatch whilst the test leader registers the number of approved jumps. The player performs two warm up trials on each foot prior to executing the test.

Ankle and knee stability

To assess stability of player's talocrural joints, a modified anterior drawer test is employed[64 65]. Furthermore, a modified version of Fairbank's apprehension test is utilised to evaluate the player's stability in the patellofemoral joint[66]. The tests are conducted on both the dominant and non-dominant foot and knee and are considered positive if the player experience any pain or discomfort during the examination, and/or an involuntary contraction of the quadriceps musculature occur during the Fairbank's apprehension test.

Isometric back extensor endurance

Isometric back extensor endurance is assessed by the modified Sorensen test[67-69]. In this previously described modified test[67 68], the player's lower body is supported byan examination table in prone position with three straps and the anterior-superior iliac spine is aligned with the edge of the table. The player is instructed to keep her arms folded across the chest throughout the procedure and isometric maintaining the upper body in a horizontal position until failure. The test leader registers the time elapsed until failure. A digital inclinometer (Clinometer, Plaincode, Stephanskirchen, Germany) is placed upon a metric ruler at the level of th5 in the thoracic spine to monitor sagittal plane movement. Prior to the assessment, the player completes a shorter warmup trial to orient the desired sagittal plane target angle.

Follow-up measurement and outcome

Follow-up measurements are collected prospectively during one year from the baseline. In the weekly online questionnaire, the players are asked to answer questions regarding new and ongoing injuries, LBP and UBNP intensity, social support, perceived stress, recovery, and to be able to consider workload, number of training and match play hours/week[70]. To assess

For peer review only - http://bmjopen.bmj.com/site/about/guidelines.xhtml

whether players sustain football related injuries throughout the follow-up period, the Swedish version of OSTRC-O is employed and included in the weekly online questionnaire[42 43 71]. Two study specific adaptions were made to the OSTRC-O. Firstly,a question regarding absence/reduced participation in training/match due to reasons not related to injuries was added. Secondly, the option to specify injuries in different anatomical localisations in the lower- and upper extremity, back, neck, head and abdomen was included.

Football related injuries reported with the OSTRC-O in the weekly online questionnaire leading to moderate or severe reductions in participation/and or sports performance or complete inability to participate in sport are classified as a substantial injury in this study[42]. Players reporting new substantial injuries are contacted via telephone by a clinically experienced research assistant to answer a standardised interview with questions concerning the injury such as: injury mechanism, localisation, type, time-loss, re-injury, diagnosis, and medical care. Injuries are divided into acute and gradual onset. An acute injury is defined as a result from a specific, identifiable event, whereas injuries with gradual onset are defined as an injury without a single, identifiable event responsible for the injury[35]. Players receive an automated link to the online questionnaire sent by email each Sunday, with a reminder email the next day to players not responding. Furthermore, if no response is received, a text message reminder with the link is sent on Tuesdays. Finally, every other week representatives of the study visit participating football clubs to collect unanswered surveys for the previous two-week period.

After 52 weeks of participation, a questionnaire with equivalent content as the baseline questionnaire (excluding OSTRC-O with 2- and 3-6-month recall) are distributed to the players to evaluate possible changes from the baseline characteristics. The first 106 included players also underwent a secondary physical test protocol after 52 weeks of follow-up. In the one-year follow-up questionnaire, different aspects of UBP and LBP, respectively, in the

For peer review only - http://bmjopen.bmj.com/site/about/guidelines.xhtml

preceding six months are measured. “Have you had UBP/Have you had LBP” (Yes/No)? If yes, has the pain hindered your daily activities (No, Yes to some extent or Yes to a high degree)? If Yes, the “Visual Trajectories Questionnaire – Pain” is used to capture the longitudinal state of a player's pain experience of UBP and LBP and are retrospectively reported for the preceding six-month period[45]. See Table 1 for an overview of the measurements during the different phases of the study.

Table 1. Summary of the included measurements during the different phases of the study.

**Phase**	**Measurements**	**Tests/tools**
Baseline: players (consecutive during inclusion; 2016-ongoing)	Demographic information, general health status (history of pain, illness, medication, plagues, menstrual cycle, back and neck pain), lifestyle (sleep patterns, resilience, food supplements, use of tobacco or alcohol), stress, socioeconomic factors (guardians’ education), football related factors (position, years of experience, injury preventive strategies.	KIC Baseline players, The Chronic Pain Questionnaire (CPQ)[44], Visual Trajectories Questionnaire-Pain (VTQ-P)[45],
	Anthropometric measurements (height, weight, leg length), and measurement of strength, mobility and control of lower extremity, trunk, and neck.	KIC test protocol
	History of injury and complaints	Modified OSTRC-O[42 43]
	Passion	Passion scale[40]
	General Health	GHQ-12[38]
	Coping strategies	Brief COPE[39]
Baseline: coaches (consecutive during	Education, years of experience, the use of warmup and stretching regime and implementation of injury prevention programs.	KIC Baseline coaches

For peer review only - http://bmjopen.bmj.com/site/about/guidelines.xhtml

inclusion; 2016-ongoing)		
Weekly follow-up: players (September 2016-ongoing)	Exposure to football training and match play	KIC weekly report
	Exposure to other physical activity. Health (e.g., stress, recovery) and social support.	
	Report on pain, injury performance complaints.	Modified OSTRC-O[42 43]
In case of a substantial injury event	Report on injury/complaint (type of injury, localisation, inciting event)	KIC medical report
One-year follow-up: players (consecutive after 52 weeks participation: 2017-ongoing)	Football related factors (position, injury preventive strategies). Health status (pain in back or neck) lifestyle (sleep patterns, resilience, food supplements, use of tobacco or alcohol, physical activity), stress, coping and passion for sport.	KIC One-year questionnaire
One-year follow-up (consecutive after 52 weeks participation in the first 106 included players)	Anthropometric measurements (height, weight, leg length), and measurement of strength, mobility and control of lower extremity, trunk, and neck.	KIC test protocol

Sample size

The statistical power for the analyses will depend on the exact research question, the number of exposed players, and whether the exposure is continuous or categorised. The sample size in

For peer review only - http://bmjopen.bmj.com/site/about/guidelines.xhtml

the KIC project is based on the definition “a substantial injury” as proposed by Clarsen et al.,[42], and back injuries in adolescent female players in a previously published study[7]. Based on a relative risk of 1.9 for a substantial injury in the back/neck, when 88 of the players are exposed, and with a power of 0.80, a significance level 5 % and with potential 10% drop out and a follow-up time of one year to identify risk factors, 420 players will be included.

Statistical methods

The data in the KIC study will be used to answer several different research questions and therefore, different analyses methods and statistics will be used. Kaplan-Meier estimates will be used to describe incidence. Cox regression analyses or discrete time survival analyses will be used to measure the associations between exposure and outcome, and to adjust for confounding. Only players without substantial injuries the two preceding months (reported in the baseline questionnaire) will be considered in the risk analyses, and stratified analyses to examine effect measure modification will be performed when relevant. The development of injuries is likely complex. This justifies why we measure an extensive number of factors so that we can consider confounders, intermediators, and effect measure modifier in these analyses. When identifying trajectories of time, and various factors Generalized Estimating Equations will be used for these analyses to consider the covariance between repeated measurements.

Time plan

Players will be recruited from 2017 and followed weekly for one year regarding injuries/complaints. Players will consecutively be invited and included from the year they turn 13 years old and play in a participating club. The inclusion of participants will continue until we reach over 400 players.

Data statement

For peer review only - http://bmjopen.bmj.com/site/about/guidelines.xhtml

The dataset and statistical codes will be available on reasonable request when the data collection is completed.

Patient and public involvement

No patient involved

For peer review only

For peer review only - http://bmjopen.bmj.com/site/about/guidelines.xhtml

ETHICS AND DISSEMINATION

The study was approved by the Regional Ethical Review Authority at Karolinska Institutet, Stockholm, Sweden (2016/1251-31/4). All participating players and their legal guardians receive written and oral information regarding the study and give their written informed consent when entering the study. Players under the age of 15 are required to have written informed consent from their legal guardians. The study will be performed in accordance with the recommendations guiding research involving human subjects adopted by the 18th World Medical Association General Assembly, Helsinki, Finland, June 1964, amended at the 64th World Medical Association General Assembly, Fortaleza, Brazil, October 2013. The study will be reported in accordance with the Strengthening the Reporting of Observational studies in Epidemiology (STROBE)[33]. The results will be presented in scientific conferences and published in peer-reviewed academic journals as well as being disseminated to the Swedish football movement through stakeholders and media.

For peer review only

For peer review only - http://bmjopen.bmj.com/site/about/guidelines.xhtml

FOOTNOTES

Acknowledgements Martin Samuelsson, Lena Holm, and Henrik Källberg have been involved in planning of study design, Joakim Bogren, Victor Ramirez Kristiansen, contributed to the first version of the draft. Students from the classes DN 47, 48, 49, 50, 51, and 53 at the Scandinavian college of Naprapathic Manual Medicine, Stockholm, Sweden are participating during inclusion of participants, the physical tests, and the prospective data collection.

Author Contributions VL, ES, MA and UT initiated the study. All authors, UT, NW, VL, MH, MW, UJ, MA, and ES conceived the study and contributed to the development of the study protocol. ES is the study guarantor. UT and NW bi-drafted the manuscript which was critically revised in steps by all co-authors. The final manuscript was approved by all authors.

Funding This study is funded by grants from Swedish Research Council for Sport Science, (grant number: P2019-0045, P2020-0100). The Swedish Naprapathic, and the Norwegian Naprapathic Associations, Active life foundation and Sophiahemmet foundation. There are no grant numbers for the latter funders (award/grant number: N/A).

Competing interests None declared.

Patient consent for publication Not required.

Provenance and peer review Not commissioned, externally peer reviewed.

E-mail addresses:

Ulrika.tranaeus@gih.se ; Nathan.weiss@shh.se; Victorlyberg@hotmail.com; martin.hagglund@liu.se; markus.walden@telia.com; urban.johnson@hh.se; martin.asker@shh.se; eva.skillgate@shh.se

For peer review only

Twitter Martin Asker @martinasker, Martin Hägglund @MHgglund, Markus Waldén @MarkusWalden, Eva Skillgate @skillgate_eva, Nathan Weiss @nathnweiss, Ulrika Tranaeus @UlrikaTranaeus

For peer review only

REFERENCES

1. Federation Internationale de Football Association F. Women's football survey. 2014. (accessed 2020-11-03).
2. López-Valenciano A, Ruiz-Pérez I, Garcia-Gómez A, et al. Epidemiology of injuries in professional football: a systematic review and meta-analysis. *Br J Sports Med* 2020;54(12):711-18. doi: 10.1136/bjsports-2018-099577 [published Online First: 2019/06/07]
3. Bennett P, Fawcett L. Trauma injuries sustained by female footballers. *Trauma* 2006;8(2):69-76. doi: 10.1177/1460408606072682
4. Hägglund M, Waldén M, Ekstrand J. Injuries among male and female elite football players. *Scand J Med Sci Sports* 2009;19(6):819-27.
5. Faude O, Junge A, Kindermann W, et al. Injuries in female soccer players - A prospective study in the German national league. *Am J Sport Med* 2005;33(11):1694-700. doi: 10.1177/0363546505275011
6. Le Gall F, Carling C, Reilly T. Injuries in young elite female soccer players. *Am J Sport Med* 2008;36(2):276-84. doi: 10.1177/0363546507307866
7. Clausen MB, Zebis MK, Moller M, et al. High Injury Incidence in Adolescent Female Soccer. *Am J Sport Med* 2014;42(10):2487-94. doi: 10.1177/0363546514541224
8. Larruskain J, Lekue JA, Diaz N, et al. A comparison of injuries in elite male and female football players: A five-season prospective study. *Scand J Med Sci Sports* 2018;28(1):237-45. doi: 10.1111/sms.12860
9. Lohmander LS, Ostenberg A, Englund M, et al. High prevalence of knee osteoarthritis, pain, and functional limitations in female soccer players twelve years after anterior cruciate ligament injury. *Arthritis Rheum* 2004;50(10):3145-52. doi: 10.1002/art.20589
10. Prien A, Prinz B, Dvorak J, et al. Health problems in former elite female football players: Prevalence and risk factors. *Scand J Med Sci Sports* 2017;27(11):1404-10. doi: 10.1111/sms.12747
11. Roos EM. Joint injury causes knee osteoarthritis in young adults. *Curr Opin Rheumatol* 2005;17(2):195-200.
12. Wylleman P, Reints A. A lifespan perspective on the career of talented and elite athletes: Perspectives on high-intensity sports. *Scand J Med Sci Sports* 2010;20:88-94. doi: 10.1111/j.1600-0838.2010.01194.x
13. Putukian M. The psychological response to injury in student athletes: a narrative review with a focus on mental health. *Brit J Sport Med* 2016;50(3):145-48. doi: 10.1136/bjsports-2015-095586
14. Van Mechelen W, Hlobil H, Kemper HC. Incidence, severity, aetiology and prevention of sports injuries. *Sports Med* 1992;14(2):82-99.
15. Dvorak J, Junge A, Chomiak J, et al. Risk factor analysis for injuries in football players - Possibilities for a prevention program. *Am J Sport Med* 2000;28(5):S69-S74.
16. Steffen K, Engebretsen L. More data needed on injury risk among young elite athletes. *Brit J Sport Med* 2010;44(7):485-89. doi: 10.1136/bjsm.2010.073833
17. Wiese-Bjornstal DM. Psychology and socioculture affect injury risk, response, and recovery in high-intensity athletes: a consensus statement. *Scand J Med Sci Sports* 2010;20(s2):103-11. doi: doi:10.1111/j.1600-0838.2010.01195.x
18. Hagglund M, Walden M. Risk factors for acute knee injury in female youth football. *Knee Surg Sport Tr A* 2016;24(3):737-46. doi: 10.1007/s00167-015-3922-z
19. Faude O, Junge A, Kindermann W, et al. Risk factors for injuries in elite female soccer players. *Brit J Sport Med* 2006;40(9):785-90. doi: 10.1136/bjsm.2006.027540
20. Steffen K, Pensgaard AM, Bahr R. Self-reported psychological characteristics as risk factors for injuries in female youth football. *Scand J Med Sci Sports* 2009;19(3):442-51. doi: 10.1111/j.1600-0838.2008.00797.x

For peer review only - http://bmjopen.bmj.com/site/about/guidelines.xhtml

21. Alahmad TA, Kearney P, Cahalan R. Injury in elite women's soccer: a systematic review. *Phys Sportsmed* 2020:1-7. doi: 10.1080/00913847.2020.1720548 [published Online First: 2020/02/07]
22. Magnusson K, Turkiewicz A, Hughes V, et al. High genetic contribution to anterior cruciate ligament rupture: Heritability ~69%. *Br J Sports Med* 2020;55(7):385-89. doi: 10.1136/bjsports-2020-102392
23. Ostenberg A, Roos H. Injury risk factors in female European football. A prospective study of 123 players during one season. *Scand J Med Sci Sports* 2000;10(5):279-85. doi: 10.1034/j.1600-0838.2000.010005279.x
24. Watson A, Brickson S, Brooks MA, et al. Preseason aerobic fitness predicts in-season injury and illness in female youth athletes. *Orthop J Sports Med* 2017;5(9) doi: 10.1177/2325967117726976
25. Soderman K, Alfredson H, Pietila T, et al. Risk factors for leg injuries in female soccer players: a prospective investigation during one out-door season. *Knee Surg Sport Tr A* 2001;9(5):313-21. doi: 10.1007/s001670100228
26. Nilstad A, Andersen TE, Bahr R, et al. Risk factors for lower extremity injuries in elite female soccer players. *Am J Sport Med* 2014;42(4):940-48. doi: 10.1177/0363546513518741
27. Blokland D, Thijs KM, Backx FJ, et al. No effect of generalized joint hypermobility on injury risk in elite female soccer players: A prospective cohort study. *Am J Sports Med* 2017;45(2):286-93. doi: 10.1177/0363546516676051
28. Feldman DE, Shrier I, Rossignol M, et al. Risk factors for the development of neck and upper limb pain in adolescents. *Spine* 2002;27(5):523-28.
29. Johnson U, Ivarsson A. Psychological predictors of sport injuries among junior soccer players. *Scand J Med Sci Sports* 2011;21(1):129-36. doi: 10.1111/j.1600-0838.2009.01057.x
30. Ivarsson A, Johnson U, Lindwall M, et al. Psychosocial stress as a predictor of injury in elite junior soccer: A latent growth curve analysis. *J Sci Med Sport* 2014;17(4):366-70. doi: 10.1016/j.jsams.2013.10.242
31. Junge A, Rosch D, Peterson L, et al. Prevention of soccer injuries: A prospective intervention study in youth amateur players. *Am J Sport Med* 2002;30(5):652-59.
32. Pensgaard AM, Ivarsson A, Nilstad A, et al. Psychosocial stress factors, including the relationship with the coach, and their influence on acute and overuse injury risk in elite female football players. *BMJ Open Sport Exerc Med* 2018;4(1):e000317. doi: 10.1136/bmjsem-2017-000317
33. von Elm E, Altman DG, Egger M, et al. The Strengthening the Reporting of Observational Studies in Epidemiology (STROBE) Statement: Guidelines for reporting observational studies. *Int J Surg* 2014;12(12):1495-99. doi: 10.1016/j.ijsu.2014.07.013
34. Walden M, Atroshi I, Magnusson H, et al. Prevention of acute knee injuries in adolescent female football players: cluster randomised controlled trial. *Bmj-British Medical Journal* 2012;344 doi: 10.1136/bmj.e3042
35. Fuller CW, Ekstrand J, Junge A, et al. Consensus statement on injury definitions and data collection procedures in studies of football (soccer) injuries. *Brit J Sport Med* 2006;40(3):193-201. doi: 10.1136/bjsm.2005.025270
36. Hägglund M, Waldén M, Bahr R, et al. Methods for epidemiological study of injuries to professional football players: developing the UEFA model. *Brit J Sport Med* 2005;39(6):340-46.
37. Emery CA, Meeuwisse WH, Hartmann SE. Evaluation of risk factors for injury in adolescent soccer: implementation and validation of an injury surveillance system. *Am J Sport Med* 2005;33(12):1882-91.
38. Hardy GE, Shapiro DA, Haynes CE, et al. Validation of the general health questionnaire-12 using a sample of employees from England's health care services. *Psychol Assess* 1999;11(2):159-65. doi: 10.1037/1040-3590.11.2.159
39. Carver CS. You want to measure coping but your protocol's too long: Consider the brief COPE. *Int J Behav Med* 1997;4(1):92-100. doi: 10.1207/s15327558ijbm0401_6

For peer review only - http://bmjopen.bmj.com/site/about/guidelines.xhtml

40. Vallerand RJ, Blanchard C, Mageau GA, et al. Les passions de l'Ame: On obsessive and harmonious passion. *J Pers Soc Psychol* 2003;85(4):756-67. doi: 10.1037/0022-3514-85.4.756
41. Salminen S, Kouvonen A, Koskinen A, et al. Is a single item stress measure independently associated with subsequent severe injury: a prospective cohort study of 16,385 forest industry employees. *Bmc Public Health* 2014;14(1):543.
42. Clarsen B, Myklebust G, Bahr R. Development and validation of a new method for the registration of overuse injuries in sports injury epidemiology: the Oslo Sports Trauma Research Centre (OSTRC) overuse injury questionnaire. *Br J Sports Med* 2013;47(8):495-502.
43. Ekman E, Frohm A, Ek P, et al. Swedish translation and validation of a web-based questionnaire for registration of overuse problems. *Scand J Med Sci* 2015;25(1):104-9. doi: 10.1111/sms.12157
44. Von Korff M, Ormel J, Keefe FJ, et al. Grading the severity of chronic pain. *Pain* 1992;50(2):133-49.
45. Dunn KM, Campbell P, Jordan KP. Validity of the visual trajectories questionnaire for pain. *J Pain* 2017;18(12):1451-58.
46. Dennis RJ, Finch CF, Elliott BC, et al. The reliability of musculoskeletal screening tests used in cricket. *Phys Ther Sport* 2008;9(1):25-33. doi: 10.1016/j.ptsp.2007.09.004
47. Youdas JW, Bogard CL, Suman VJ. Reliability of goniometric measurements and visual estimates of ankle joint active range of motion obtained in a clinical setting. *Arch Phys Med Rehab* 1993;74(10):1113-18. doi: 10.1016/0003-9993(93)90071-h
48. Ness BM, Sudhagoni RG, Tao H, et al. The reliability of a novel heel-rise test versus goniometry to assess plantarflexion range of motion. *Int J Sports Phys Ther* 2018;13(1):19-27. doi: 10.26603/ijspt20180019
49. Konor MM, Morton S, Eckerson JM, et al. Reliability of three measures of ankle dorsiflexion range of motion. *Int J Sports Phys Ther* 2012;7(3):279.
50. Frohm A, Heijne A, Kowalski J, et al. A nine-test screening battery for athletes: a reliability study. *Scand J Med Sci* 2012;22(3):306-15. doi: 10.1111/j.1600-0838.2010.01267.x
51. Asker M, Walden M, Kallberg H, et al. A prospective cohort study identifying risk factors for shoulder injuries in adolescent elite handball players: the Karolinska Handball Study (KHAST) study protocol. *BMC Musculoskelet Disord* 2017;18 doi: 10.1186/s12891-017-1852-2
52. Johnson KD, Kim KM, Yu BK, et al. Reliability of thoracic spine rotation range-of-motion measurements in healthy adults. *J Athl Training* 2012;47(1):52-60.
53. Andre MJ, Fry AC, Heyrman MA, et al. A reliable method for assessing rotational power. *J Strength Cond Res* 2012;26(3):720-24. doi: 10.1519/JSC.0b013e318227664d
54. Palmer TG, Uhl TL. interday reliability of peak muscular power outputs on an isotonic dynamometer and assessment of active trunk control using the chop and lift tests. *J Athl Training* 2011;46(2):150-59. doi: 10.4085/1062-6050-46.2.150
55. Zois J, P Sharp A, Talukdar K, et al. The reliability of a rotational power assessment of the core. *J Athl Enhanc* 2016;5(5)
56. Arumugam A, Mani R, Raja K. Interrater reliability of the craniocervical flexion test in asymptomatic individuals- A cross-sectional study. *J Manipulative Physiol Ther* 2011;34(4):247-53. doi: 10.1016/j.jmpt.2011.04.011
57. James G, Doe T. The craniocervical flexion test: intra-tester reliability in asymptomatic subjects. *Physiother Res Int* 2010;15(3):144-49.
58. Thorborg K, Bandholm T, Holmich P. Hip- and knee-strength assessments using a hand-held dynamometer with external belt-fixation are inter-tester reliable. *Knee Surg Sport Tr A* 2013;21(3):550-55. doi: 10.1007/s00167-012-2115-2
59. Kelln BM, McKeon PO, Gontkof LM, et al. Hand-held dynamometry: Reliability of lower extremity muscle testing in healthy, physically active, young adults. *J Sport Rehabil* 2008;17(2):160-70. doi: 10.1123/jsr.17.2.160

For peer review only - http://bmjopen.bmj.com/site/about/guidelines.xhtml

60. Prather H, Harris-Hayes M, Hunt DM, et al. Reliability and agreement of hip range of motion and provocative physical examination tests in asymptomatic volunteers. *PM R* 2010;2(10):888-95. doi: 10.1016/j.pmrj.2010.05.005
61. Nussbaumer S, Leunig M, Glatthorn JF, et al. Validity and test-retest reliability of manual goniometers for measuring passive hip range of motion in femoroacetabular impingement patients. *BMC Musculoskelet Disord* 2010;11 doi: 10.1186/1471-2474-11-194
62. Sharma N, Sharma A, Sandhu JS. Functional performance testing in athletes with functional ankle instability. *Asian J Sports Med* 2011;2(4):249.
63. Caffrey E, Docherty CL, Schrader J, et al. The ability of 4 single-limb hopping tests to detect functional performance deficits in individuals with functional ankle instability. *J Orthop Sports Phys Ther* 2009;39(11):799-806. doi: 10.2519/jospt.2009.3042
64. Vaseenon T, Gao Y, Phisitkul P. Comparison of two manual tests for ankle laxity due to rupture of the lateral ankle ligaments. *Iowa Orthop J* 2012;32:9-16.
65. de Vries JS, Kerkhoffs G, Blankevoort L, et al. Clinical evaluation of a dynamic test for lateral ankle ligament laxity. *Knee Surg Sport Tr A* 2010;18(5):628-33. doi: 10.1007/s00167-009-0978-7
66. Smith TO, Clark A, Neda S, et al. The intra- and inter-observer reliability of the physical examination methods used to assess patients with patellofemoral joint instability. *Knee* 2012;19(4):404-10. doi: 10.1016/j.knee.2011.06.002
67. Moreau CE, Green BN, Johnson CD, et al. Isometric back extension endurance tests: A review of the literature. *J Manipulative Physiol Ther* 2001;24(2):110-22. doi: 10.1067/mmt.2001.112563
68. Demoulin C, Vanderthommen M, Duysens C, et al. Spinal muscle evaluation using the Sorensen test: a critical appraisal of the literature. *Joint Bone Spine* 2006;73(1):43-50. doi: 10.1016/j.jbspin.2004.08.002
69. Latimer J, Maher CG, Refshauge K, et al. The reliability and validity of the Biering-Sorensen test in asymptomatic subjects and subjects reporting current or previous nonspecific low back pain. *Spine* 1999;24(20):2085-89. doi: 10.1097/00007632-199910150-00004
70. Malone S, Owen A, Newton M, et al. The acute:chonic workload ratio in relation to injury risk in professional soccer. *J Sci Med Sport* 2017;20(6):561-65. doi: 10.1016/j.jsams.2016.10.014
71. Nilstad A, Bahr R, Andersen TE. Text messaging as a new method for injury registration in sports: A methodological study in elite female football. *Scand J Med Sci Sports* 2014;24(1):243-49. doi: 10.1111/j.1600-0838.2012.01471.x

For peer review only

This is a supplementary document describing the included tests in Karolinska football Injury Cohort, KIC. Table S1 shows the test stations, number of test leaders and randomization of the tests. The persons in the images have given their consent that the images will be used in publications related to this study.

Calf heel raises

Ankle plantarflexion (PF) muscle endurance is investigated using unilateral barefoot weight bearing calf heel raises[46]. Firstly, the player's maximal weight being PF range of motion (ROM) is obtained by painting a reference mark on the player's heel at floor level and registering the maximal height achieved during one calf heel raise with a metric ruler.

The player is thereafter instructed to perform repeated maximum unilateral heel raises until failure, guided by a metronome to standardize the pace (1 second concentric-, 1 second eccentric contraction). The player is allowed to have light contact with her fingers against a wall. A repetition is considered approved on the basis whether knee extension is maintained, and the reference mark on the player's heel levels with the registered maximal PF ROM height on the ruler. The test leader registers the total number of approved repetitions and discontinues the test when the player fails to reach the marked maximal height. The same procedure is then conducted on the opposite foot. The order of execution is randomized prior to the test.

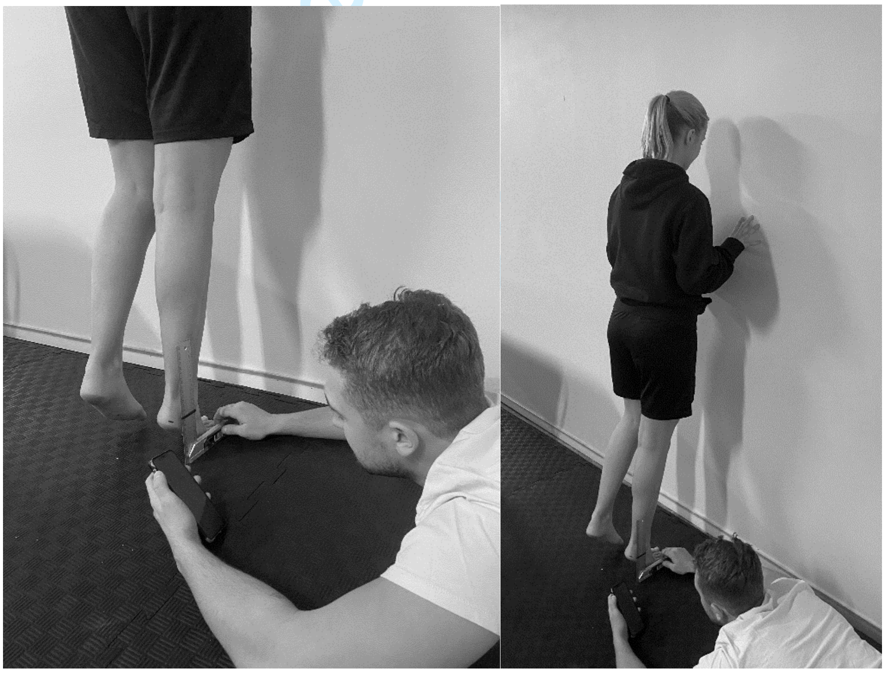

Figure S1. Calf heel raises.

Active plantarflexion mobility

Active PF ROM is measured with a clear plastic goniometer positioned at the lateral malleolus, utilizing fibula and fifth metatarsal as reference marks[47 48]. The player is positioned in supine on an treatment table, with feet off the edge of the table. The player is instructed to perform a sequence of six maximal active PF cycles starting from a neutral dorsiflexion (DF) position, whilst maintaining extended knees throughout the movement. The test leader measures and registers the maximal PF ROM in the final three cycles.

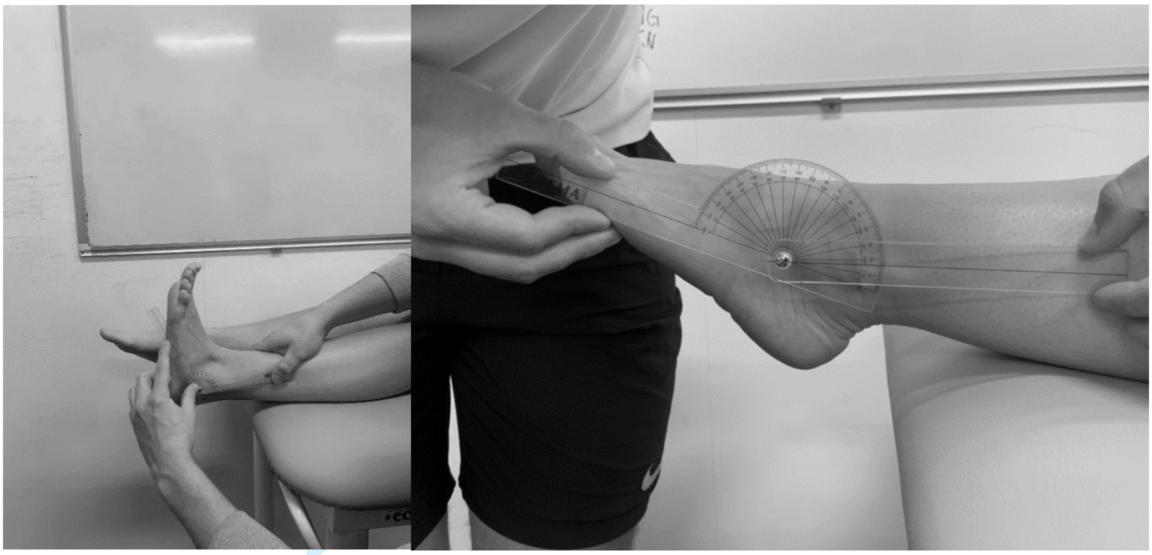

Figure S2. Active plantarflexion mobility execution. a) starting position in neutral dorsiflexion, b) end position in maximal active plantarflexion.

Weight bearing ankle dorsiflexion mobility

Weight bearing ankle DF ROM is measured in a standing lunge position with the player's foot placed upon a metric ruler 10 cm away from a wall to the player's greater toe[46 49]. The player is instructed to lunge forward, directing the knee in line with her second toe, until contact with the wall is achieved; without allowing the heel to lift off the ground, which is continuously monitored through the availability to maintain a piece of paper against the foundation. Throughout the test, the player is allowed to provide balance by light contact with her fingers against the wall.

Firstly, three consecutive warm-up trials are performed from the 10 cm mark to familiarize the player with the test. Thereafter, the test leader measures the following three trials. In each trial, the player begins from the reference mark (10 cm) and progresses 1 cm away from the wall at a time, until unable to perform a successful repetition. If the player is unable to perform an approved repetition at the 10 cm reference mark, she is asked to progress 1 cm forward until able to complete a successful repetition. Once the player achieves knee-wall contact, the DF ROM is measured with a digital inclinometer (Clinometer, Plaincode, Stephanskirchen, Germany) and the distance from the wall to the greater toe is measured in cm in the repetition furthest away from the wall in each trial.

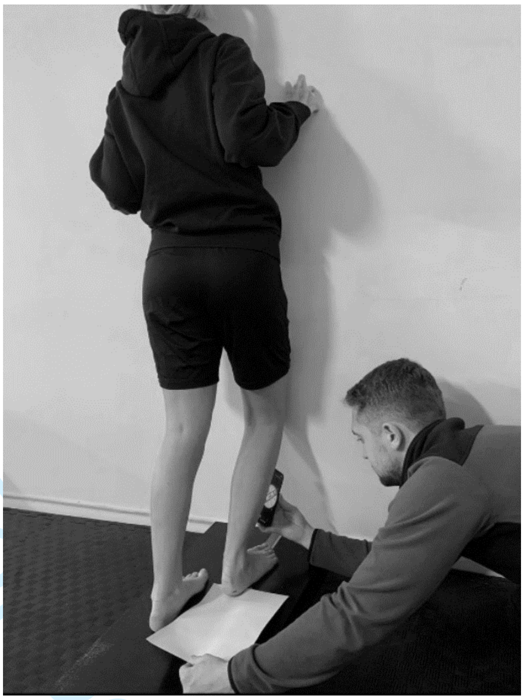

Figure S3. Weight bearing ankle dorsiflexion mobility.

Trunk mobility

Mobility in trunk rotation are measured in a cross-legged seated position, and in a lunge position with the player on a gym mat, graded with 5 degrees increments, from zero to one hundred and eighty degrees[50-52].

In the seated test (modified seated rotation test), the player is positioned at the center of the gym mat, in a cross-legged position with a wooden stick resting on the shoulders whilst keeping her arms crossed. If the player is unable to achieve the cross-legged sitting position, she is allowed to sit comfortable in an ordinary sitting position, which is noted by the test leader. Once in the starting position, the player is instructed to keep an upright posture and maximally rotate alternating between right and left for three times, whilst the test leader measures the rotational degrees in the end range.

The same procedure is thereafter repeated in a lunge position with the wooden stick resting on the player's shoulders. The player is positioned in a lunge position with her posterior knee at the center of the gym mat, and with her feet aligned on the zero-degree mark. Three consecutive maximal rotations are carried out alternating between right and left and is conducted in a lunge position for both the dominant, and non-dominant limb.

For peer review only

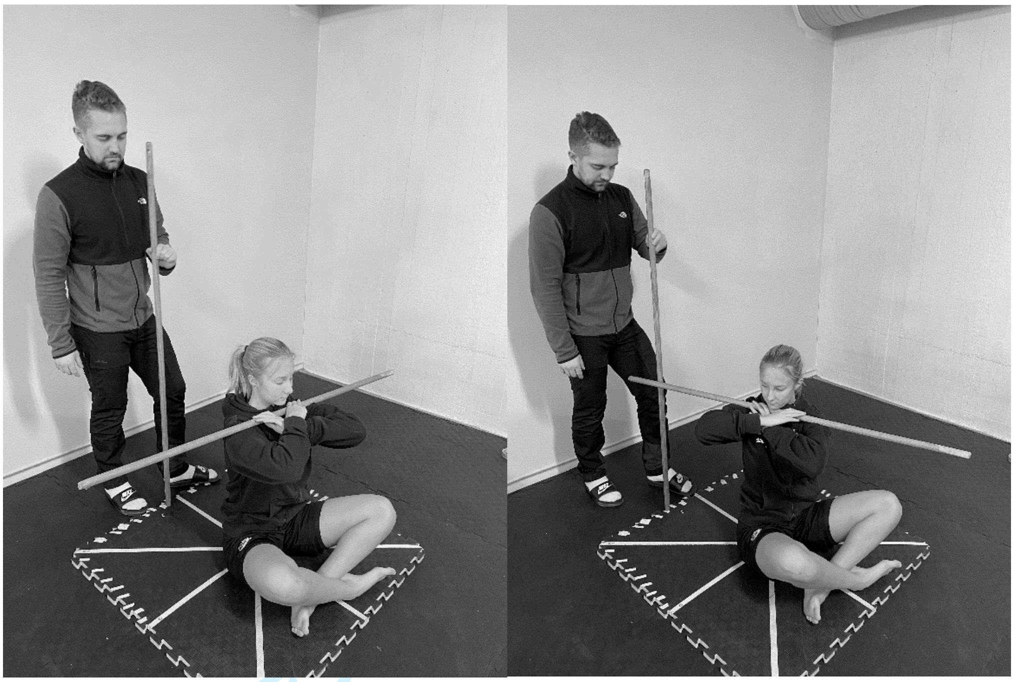

Figure S4. Modified seated rotation test. a) starting position, b) end position (right).

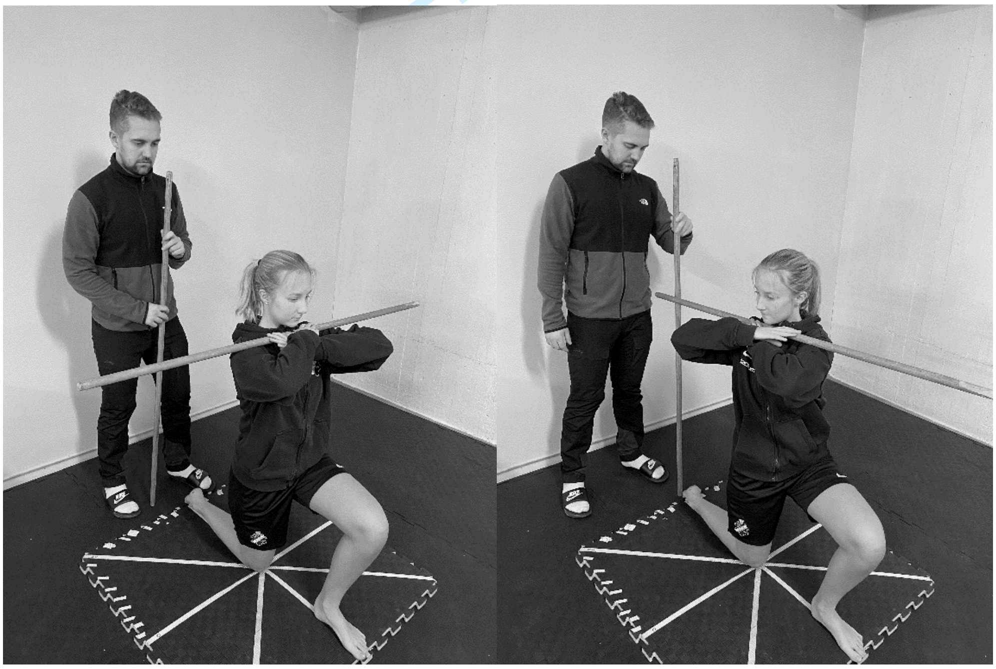

Figure S5. lunge rotation test. a) starting position left leg, b) end position (right).

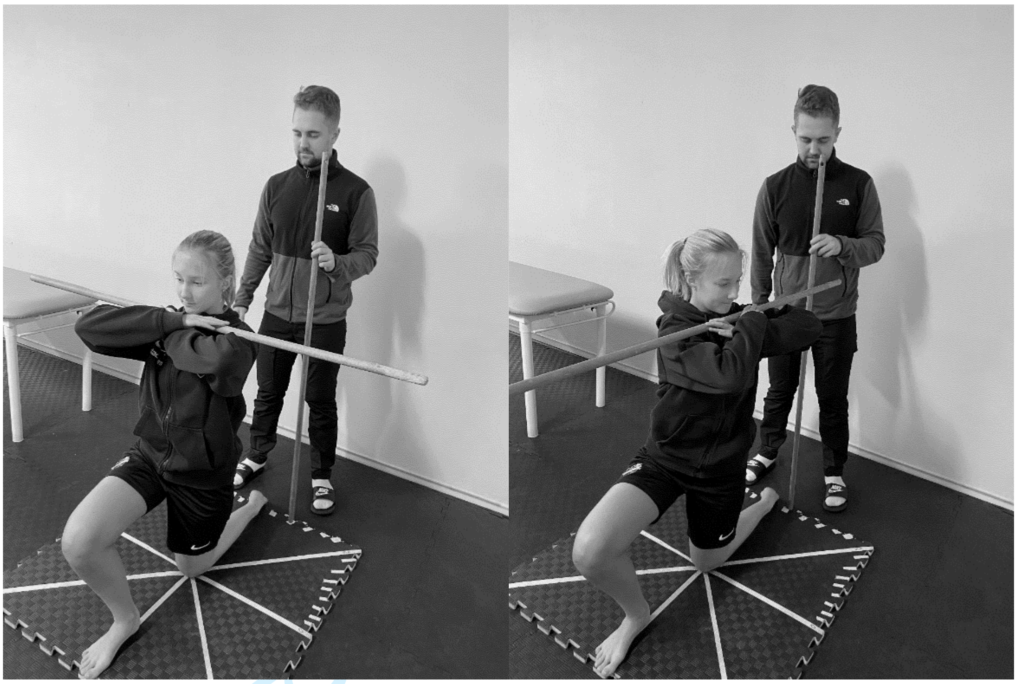

Figure S6. lunge rotation test. a) starting position right leg, b) end position (left).

Trunk strength

Isometric trunk rotational strength is measured in a modified standing wood chopper test utilizing a force gauge to evaluate force output (RS Pro Digital Force Gauge, RS Components Ltd., Corby, UK) [53-55].

In a standing position with extended arms, the player holds a handle in shoulder height, which is attached to the force gauge. The test leader positions the player in a 30-degree trunk rotation in the horizontal plane towards the anchor point (see figure S7).

The player is thereafter instructed to maximally generate force through her trunk and isometrically rotate in the opposite direction for five seconds whilst maintaining straight arms. Three consecutive repetitions are conducted for both right and left, and the maximal force output generated is used in the analyses. The order of execution is randomized prior to performing the test.

For peer review only - http://bmjopen.bmj.com/site/about/guidelines.xhtml

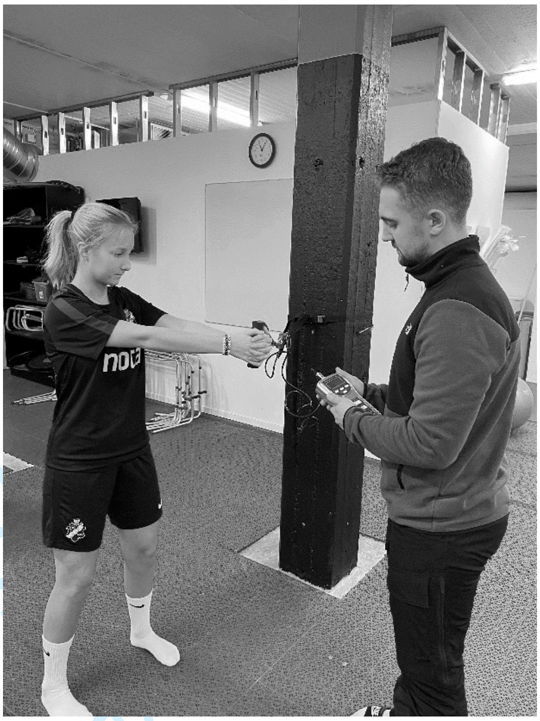

Figure S7. Modified standing wood chopper test (isometric rotation to the right).

Deep neck flexor endurance

Deep neck flexor muscle endurance is assessed through a modified version of the Cranio-cervical flexion test (CCFT) with a pressure sensor (Stabilizer Pressure Bio-Feedback, Chattanooga Group inc, Hixon, TN) [51 56 57].

Prior to executing the test, the player is instructed in how to perform a correct cranio-cervical flexion motion in standing and supine position through a gentle 'head nodding' cue. The player is positioned in supine position on a treatment table with her hands placed upon her abdomen or at the side of the body and with her feet on the table, with flexed hips and knees. With the player's head and neck in a neutral position, the pressure stabilizer is positioned sub-occipitally, and inflated to a baseline pressure of 20 mmHg. Firstly, a pre-test is conducted and later an endurance test.

During the pre-test, the player is instructed to perform a gentle cranio-cervical flexion to increase the pressure starting from a baseline of 20 mmHg with 2 mmHg increments to a maximum of 30 mmHg. 3x3 second contractions are carried out at each target pressure (TP) with a three second rest in between each contraction whilst the test leader monitors for potential compensational strategies: excessive use of global neck musculature, chin jerking, cervical spine retraction, jaw clenching, breath holding and a pressure loss of ≥ 2 mmHg. A stopwatch time the contractions and visual feedback of pressure level is provided by the test leader who holds the manometer dial so that both the player and the test leader can read it throughout the procedure.

The endurance test is conducted if the player completes each of the five TP (22, 24, 26, 28 and 30 mmHg) without exhibiting any of the compensational strategies and/or experiencing pain during the pre-test. During the endurance test, the same setup and procedure as in the

For peer review only

pre-test is carried out. The player is now instructed to hold each contraction at the TP for 3x10 seconds with a ten second rest in between contractions. The highest completed TP with a full set of 3x10 seconds contractions is registered by the test leader and later used for analysis.

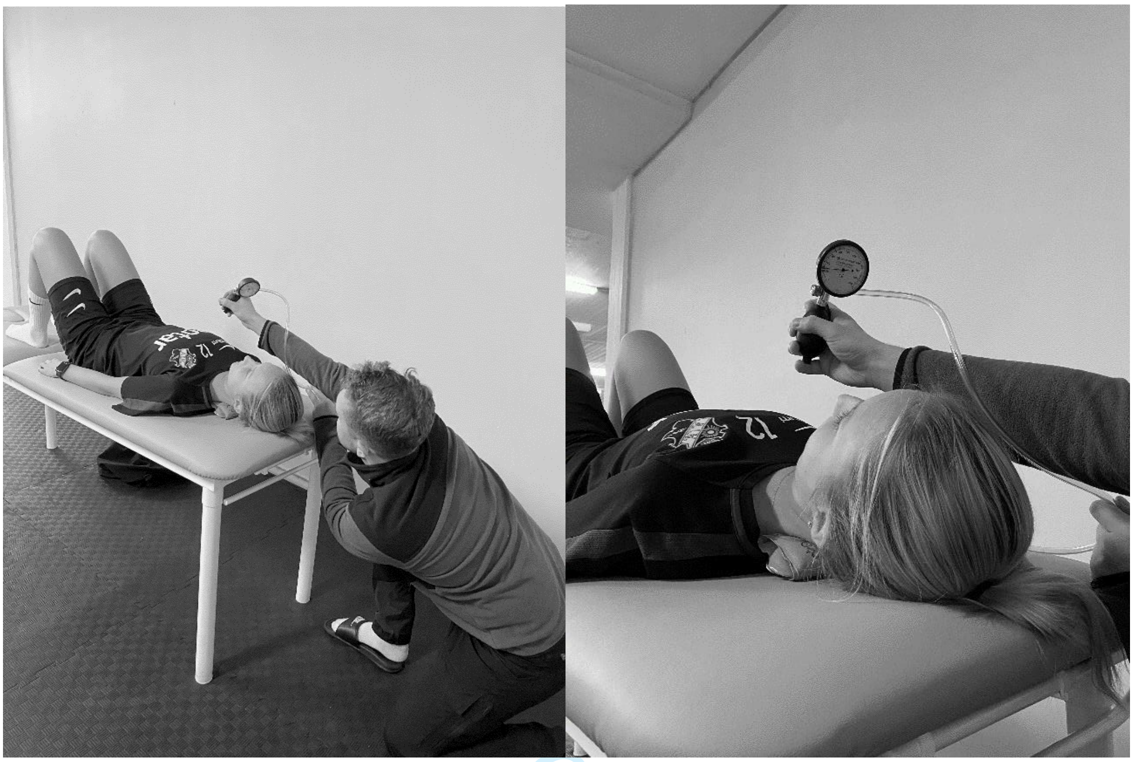

Figure S8. Modified cranio-cervical flexion test.

Hip- and knee strength

Isometric hip flexion, extension, adduction, and abduction strength as well as eccentric hip abduction and adduction and isometric knee extension strength are measured with a hand-held dynamometer (HHD) (MicroFet2, Hoggan Health Industries inc. West Jordan, UT, USA)[58 59].

Prior to executing the strength tests, two submaximal isometric contractions in each direction are performed to familiarize the player with the procedures. Three isometric contractions with gradually increasing force output for five seconds, and three maximal eccentric contractions for three seconds are performed in the isometric and eccentric tests, respectively, with a 10 second rest in between each contraction. The maximal force output for each position is registered by the test leader and later used for analysis. The order of execution and starting side is randomized prior to performing the tests at the particular test station (see table S1).

Isometric hip flexion strength

The player is positioned in a seated position at the edge of an treatment table, with 90-degrees of hip- and knee flexion. The HHD is positioned two centimeters proximal to the patella, and are externally fixated with a belt, which is secured under the leg of the treatment table, limiting hip flexion movement. The player is instructed to perform three isometric contractions on each leg.

For peer review only

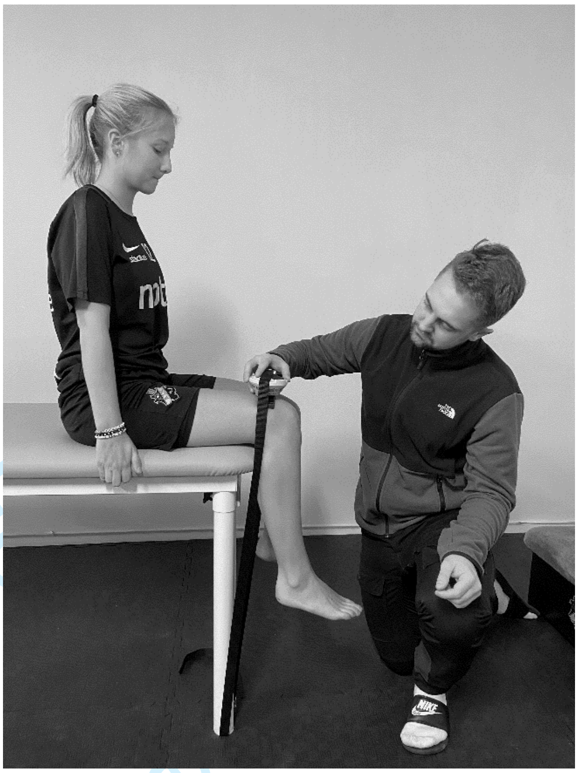

Figure S9. Isometric hip flexion strength (right hip).

Isometric knee extension strength

Seated in the same position as during the isometric hip flexion strength test, with a slightly extended knee joint, the HHD is positioned two centimeters proximal to the malleoli on the anterior aspects of the player's tibia and are externally fixated with a belt. The player is instructed to perform three isometric contractions on each leg respectively.

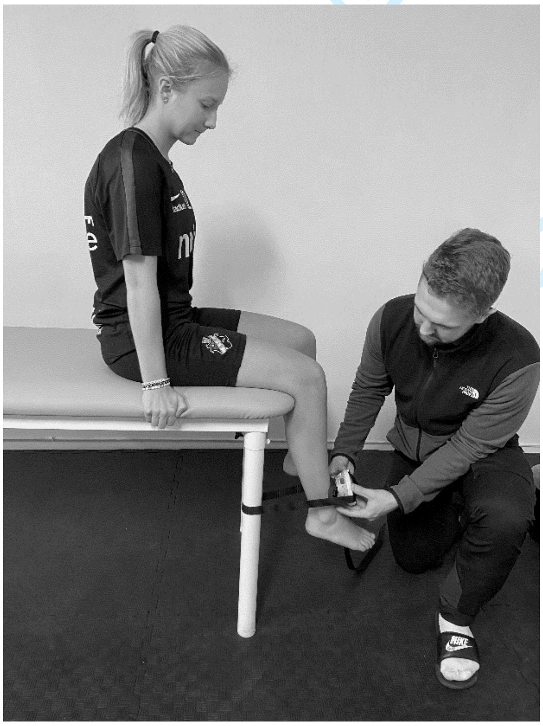

Figure S10. Isometric knee extension strength (right leg).

Isometric hip extension strength

With the player positioned in a prone position on a treatment table and with her feet off the edge of the table, the test leader externally fixates the HHD two centimeters proximal to the malleoli with a belt. Furthermore, the player is instructed to perform three maximal isometric contractions on each leg respectively

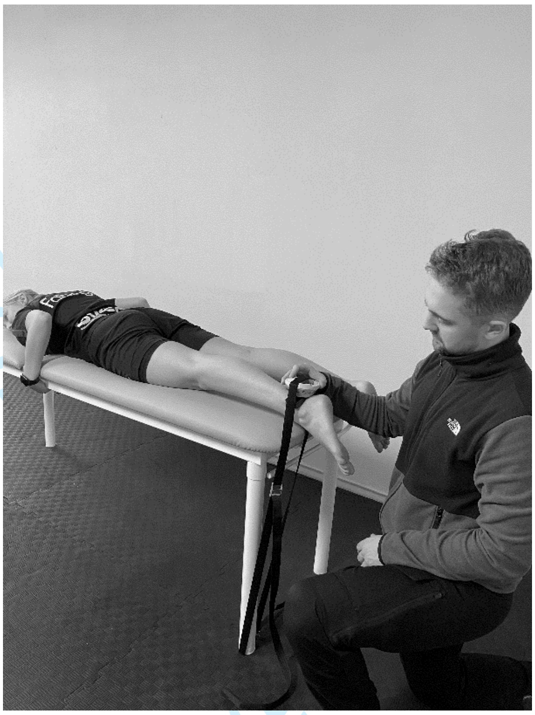

Figure S11. Isometric hip extension strength (left hip).

Isometric hip abduction strength

The player is positioned in a supine position on an treatment table, with the tested leg extended, and the non-tested leg flexed. The test leader positions and fixates the HHD two centimeters proximal to the lateral malleolus with a belt, which limits hip abduction movement. Thereafter, the player is instructed to perform three maximal isometric hip abductions, whilst the test leader measures the force output for both the left, and right side.

For peer review only

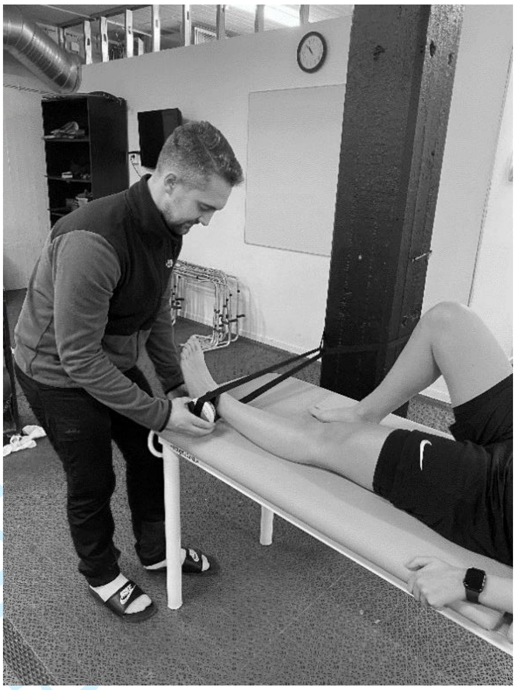

Figure S12. Isometric hip abduction strength (left hip).

Isometric hip adduction
Lying in the same position as during the isometric hip abduction the test leader places and fixates the HHD two centimeters proximal to the medial malleolus with a belt. Consequently, the player executes three maximal isometric hip adductions on the left and right side, whilst the test leader registers the force output.

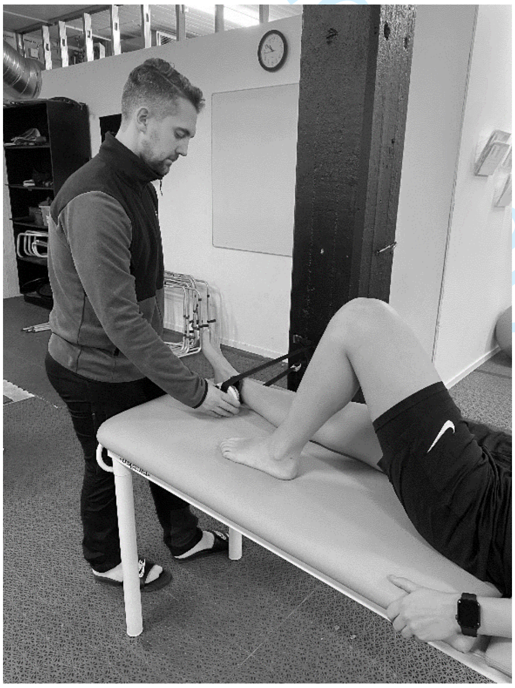

Figure S13. Isometric hip adduction strength (right hip).

For peer review only

Eccentric hip abduction strength

The player is in a side-lying position on an treatment table with the test leg extended, and the opposite leg flexed to 90 degrees in the knee- and hip joint, whilst a neutral hip position is maintained. The player is subsequently instructed to place the test leg in approximately 40 degrees of hip abduction, and the test leader places a HHD one centimeter proximally to the lateral malleolus. The test leader initiates the test by saying "push", and when the player has built up a maximal isometric contraction, the test leader begins to apply a downward directed force with the HHD whilst the player resists eccentrically for five seconds. Three repetitions are carried out on both the right and leg left, and the maximal force output is later used for analysis.

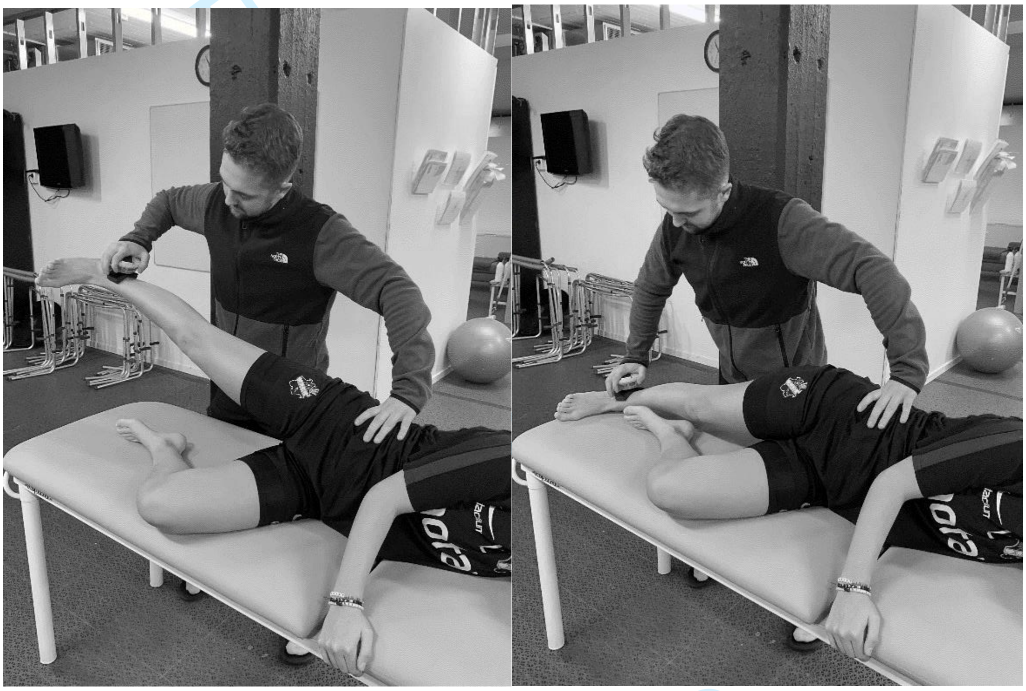

Figure S14. Eccentric hip abduction strength. a) starting position (right hip), b) end position (right hip).

Eccentric hip adduction strength

The player is positioned in the same manner as in the eccentric hip abduction strength test, with the tested leg extended, and the non-tested leg flexed in the hip- and knee joint. Thereafter, the player is instructed to place the test leg in a maximal adduction position, whereupon the test leader positions a HHD one centimeter proximally to the medial malleolus. The test is initiated when the test leaders says "push", whereupon a downward directed force is applied with the HHD whilst the player resists eccentrically for five seconds. Three consecutive trials are conducted on both sides, and the test leader registers the force output.

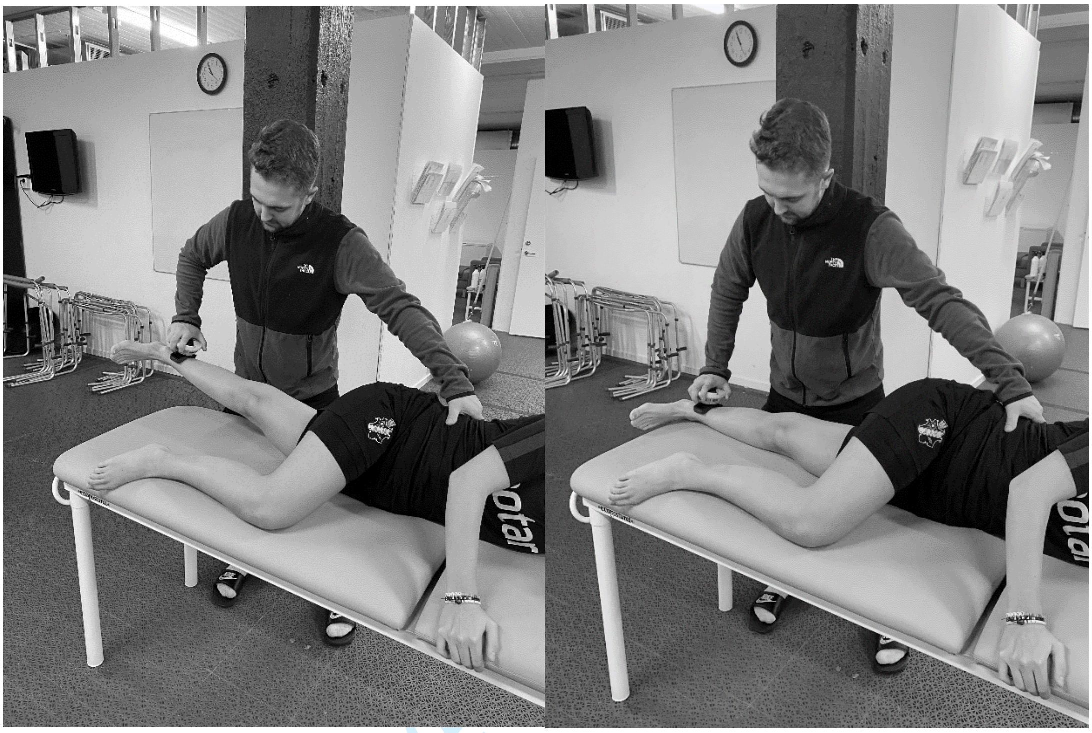

Figure S15. Eccentric hip abduction strength. a) starting position (left hip), b) end position (left hip).

Hip mobility

Measures of passive hip ROM in flexion, extension, abduction, internal- and external rotation are obtained using a universal clear plastic goniometer[60 61]. Three consecutive measurements for each position are performed for both the dominant and the non-dominant leg and the mean value for each position is later used for analysis. If the same value is obtained during the first and second measurement for a particular movement, a third one is not performed. The order of execution (side and movement) is randomized prior to performing the measures.

Passive hip flexion ROM

The player is positioned in supine position on a treatment table. With the player's leg held in a 90-degree knee flexion, test leader 1 moves the player's leg into a passive hip flexion until a firm end feel is achieved, and a posterior pelvic tilt occurs. Once the end feel is achieved, test leader 2 places the center of the goniometer at the greater trochanter and aligns one of the goniometer's arms with the player's femur, and the other one horizontally with the treatment table to read the goniometer. Three consecutive measures are conducted on each hip.

For peer review only

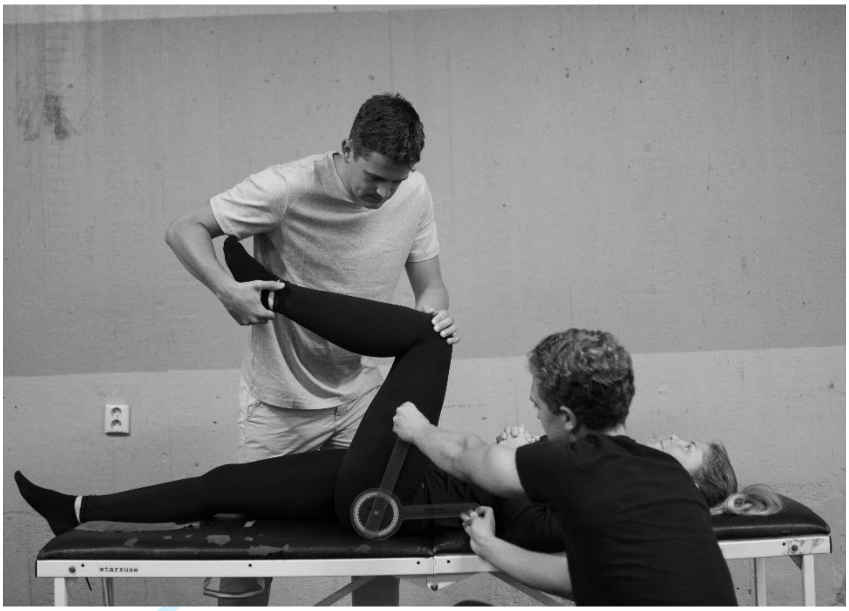

Figure S16. Passive hip flexion ROM (left hip).

Passive hip abduction ROM

The player is in a supine position on an treatment table with extended legs. While palpating the player's ipsilateral anterior superior iliac spine, test leader 1 holds the player's leg by the ankle and moves the leg into passive hip abduction until a firm end feel is achieved, and motion is felt at the pelvis. Thereafter, test leader 2 positions the goniometer at the player's hip, aligning the lever arms with the player's anterior superior iliac spine and femur, and reads the degrees of abduction. The test is repeated three times on each hip.

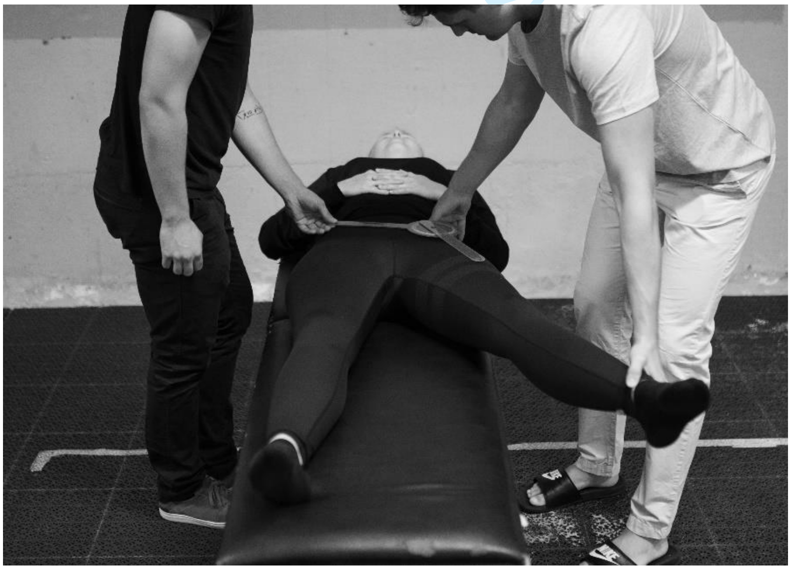

Figure S17. Passive hip abduction ROM (left hip).

Passive hip extension ROM

In prone position with extended legs, test leader 1 fixates the player's pelvis by placing a hand at the ipsilateral posterior superior iliac spine. Thereafter, while holding the player's leg at the knee, test leader 1 moves the player's leg into passive hip extension, until an end feel is achieved, indicated by an anterior tilt of the pelvis. Test leader 2 measures the degrees of passive hip flexion with the goniometer's center positioned at the greater trochanter, and the lever arms in line with the player's femur and the treatment table horizontally. Three measures are performed on each leg.

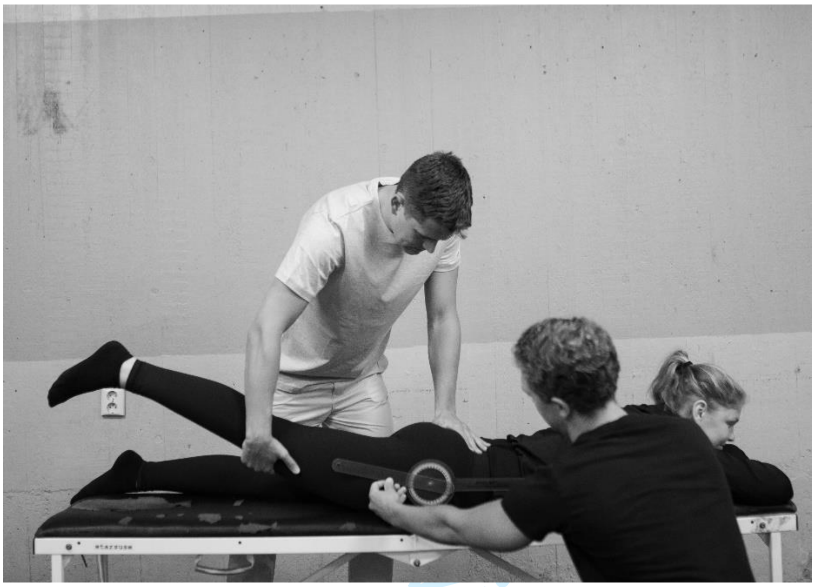

Figure S18. Passive hip extension ROM (right hip).

Passive hip internal- and external rotation ROM

In prone position, the player's leg is flexed to 90 degrees in the knee joint. Consequently, test leader 1 fixates the pelvis by placing his/her hand on the player's posterior superior iliac spine and performs a passive internal and external hip rotation, respectively, until an end feel is felt, indicated by an anterior pelvic tilt. Test leader 2 measures the degree of rotational mobility with a goniometer positioned at the knee, with the levers aligned with the player's tibia and with the treatment table horizontally. Three consecutive measures are conducted on each leg.

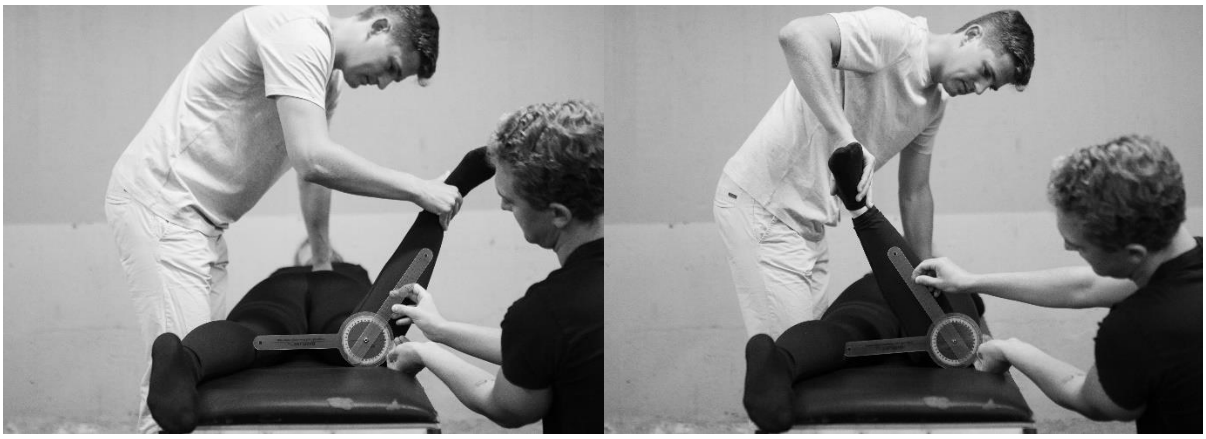

Figure S19. Passive hip rotational ROM (right hip). a) internal rotation, b) external rotation.

For peer review only - http://bmjopen.bmj.com/site/about/guidelines.xhtml

Jump performance tests

To assess the player's unilateral jump performance, the One-leg Long Box Jump Test (OLLBJ) and square hop test are performed[62 63]. A 40x40 cm square is marked on the foundation and later utilized as a reference mark in both tests. During the jump tests, players wear indoor sporting shoes.

One-leg long box jump test (OLLBJ)

Firstly, the starting position, i.e. the distance player's jump from to the 40x40 cm square is calculated by dividing the player's height in cm with 1.6 (height/1.6 = distance to the square). The player is instructed to stand on one leg at the starting position, and to perform a one-legged jump aiming inside the boundaries of the 40x40 cm square, and to maintain balance after landing. A trial is considered approved on the basis that the player land inside the 40x40 cm square, and adequately maintains balance after landing. The player performs three warm up trials on each leg, to familiarize with the procedure, and later five consecutive test trials. The test leader registers the total number of approved trials on each leg (0 to 5).

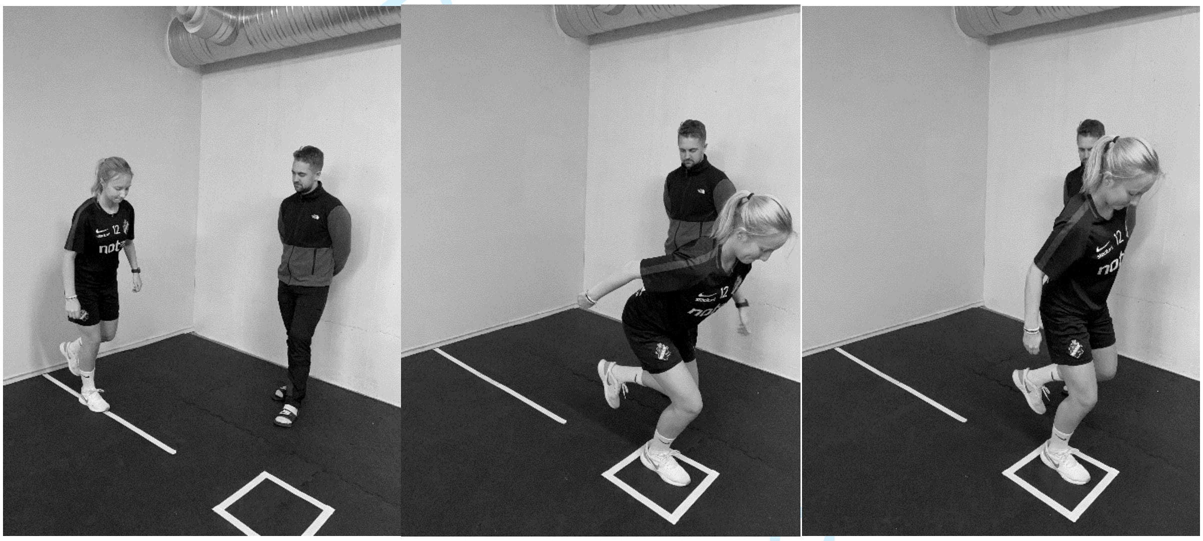

Figure S20. One-leg long box jump test (right leg). a) starting position, b) landing, c) balance maintained.

Square hop test

During the square hop test, the player is instructed to hop on one leg in and out of the 40x40 cm square as many times as possible for 15 seconds in a clockwise direction, timed with a stopwatch, whilst the test leader registers the number of approved hops. A hop is classified as approved on the basis whether the player begins a hop in the starting position (outside the square) and then executes the short hop task inside the square and then in the correct direction outside the square. Prior to the test, the player performs two warm up trials on each foot.

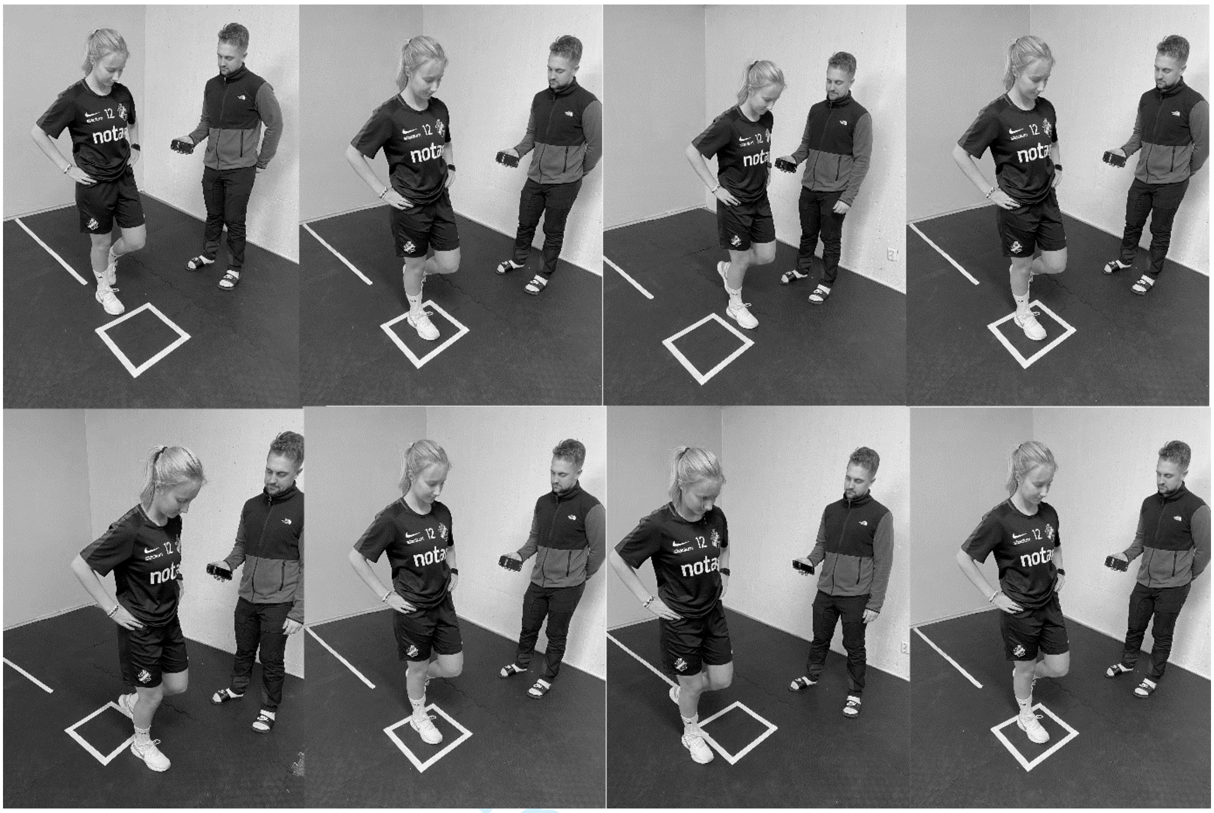

Figure S21. A series of square hop tests illustrated on the player's right leg.

Ankle- & knee stability

Modified anterior drawer test (ankle)

To assess talocrural stability or pain, a modified anterior drawer test is performed[64 65]. With the player in supine position with the test limb in knee flexion and the on the treatment table, the test leader applies an anteriorly directed force to the player's talus and a concurrent posteriorly directed force to the calcaneus. The test is conducted once on both the dominant and non-dominant foot and are considered positive if the player experiences any pain or discomfort during the procedure.

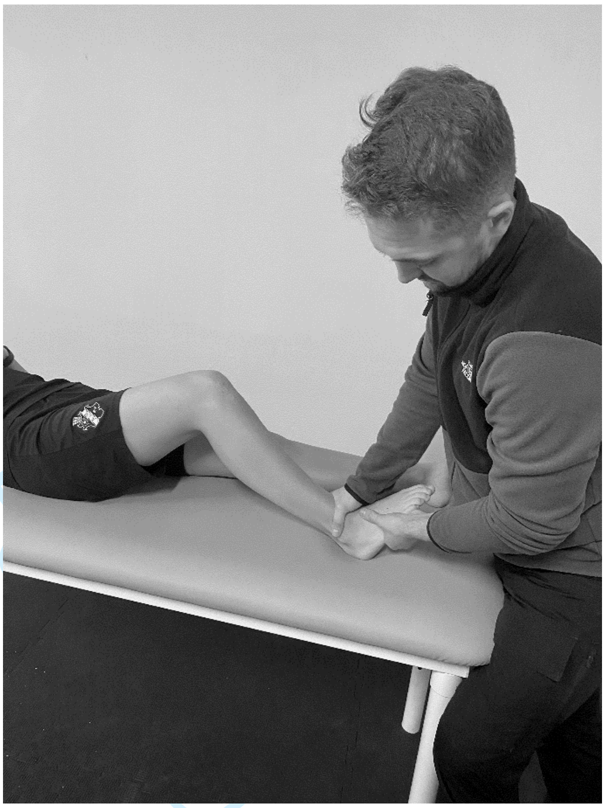

Figure S22. Modified anterior drawer test (right ankle).

Modified Fairbank's apprehension test (patellofemoral)

A modified version of Fairbank's apprehension test is conducted to evaluate stability or pain in the patellofemoral joint[66]. In supine position with extended legs, the test leader applies a laterally and subsequently medially directed force to the patella. The test is considered positive if the player experiences any pain or discomfort during the test, and/or an involuntary contraction of the quadriceps musculature. The test is carried out once on the player's dominant and non-dominant limb.

For peer review only

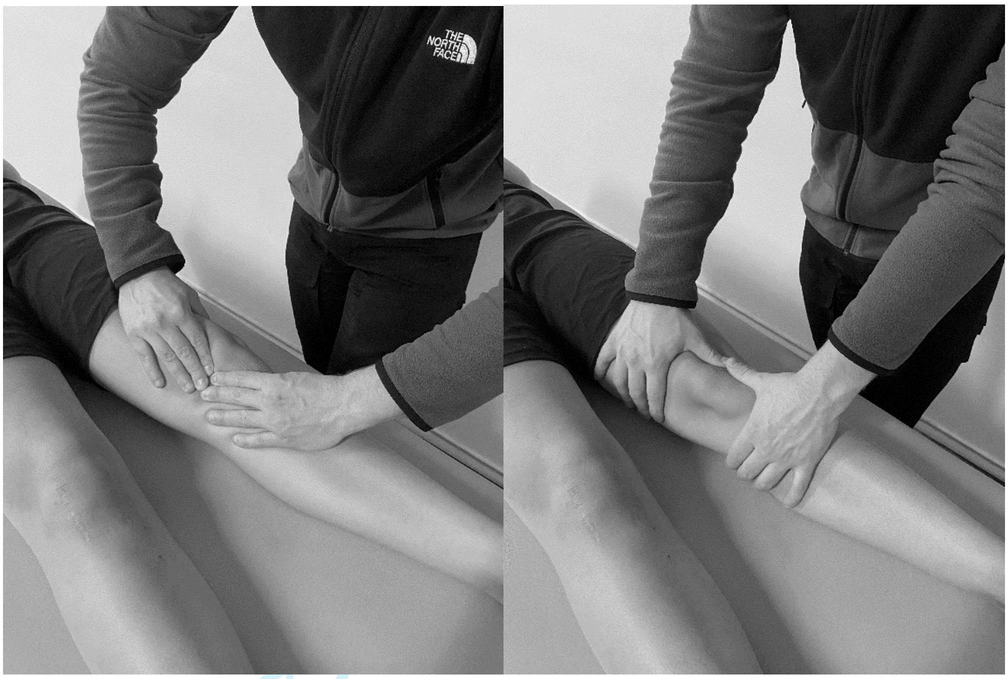

Figure S23. Modified Fairbank's apprehension test (left patellofemoral joint).
a) lateral translation, b) medial translation.

Isometric back extensor endurance

Isometric back extensor endurance is assessed by a modified Sorensen test[67-69]. In prone position, the player's anterior superior iliac spine is positioned at the edge of the treatment table. The player´s lower body is supported to the treatment table with three straps positioned over the player's ankles, knees, and pelvis. Whilst the test leader fastens the player's lower body to the treatment table with the three straps, the player uses a box/stool for support.

The player is thereafter instructed to keep her arms folded across the chest and isometrically maintain the upper body in a horizontal position until failure whilst the test leader register the time elapsed. A digital inclinometer (Clinometer, Plaincode, Stephanskirchen, Germany) is placed upon a metric ruler at the level of the 5th vertebra of the thoracical spine to monitor sagittal plane movement. If the player's upper body deviate greater than 10 degrees in the sagittal plane on more than two occasions and/or experience pain during the procedure, the test is stopped. Prior to the test, the player completes a shorter warmup trial of 5 seconds to orient the desired sagittal plane target angle.

For peer review only

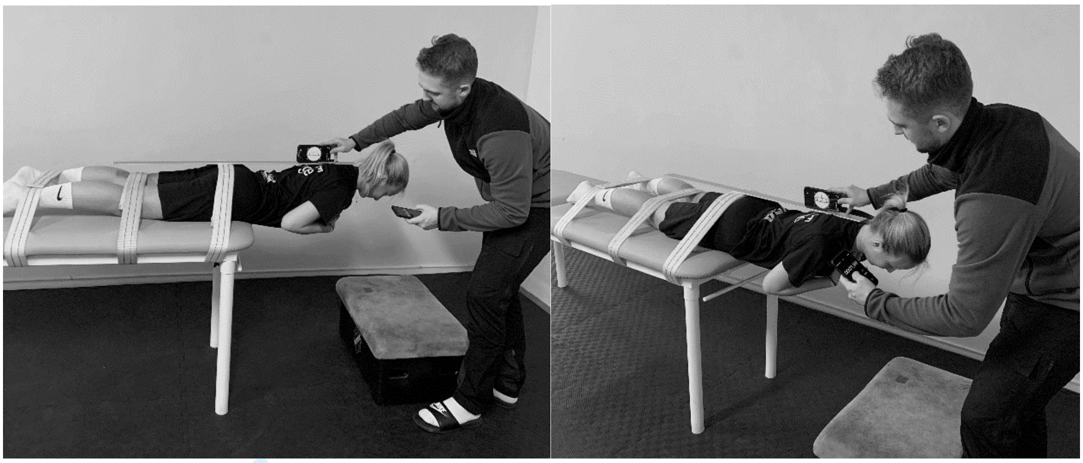

Figure S24. Modified Sorensen test.

Table S1. Test stations, number of test leaders and randomization of the physical test protocol.

Test	Test station	Number of test leaders	Randomized order of execution
Calf heel raises	1	1	Yes
Active plantarflexion mobility	2	1	No
Weight bearing ankle dorsiflexion mobility	2	1	No
Ankle- & Knee stability	2	1	No
Hip mobility	3	2	Yes
Isometric Knee extension, hip flexion & extension strength	4	2	Yes
Trunk mobility	5	1	No
Trunk strength	5	1	Yes
Isometric and eccentric hip abduction and adduction	6	2	Yes
Deep neck flexor endurance	7	1	n/a
Functional performance tests	8	1	Yes
Isometric back extensor endurance	9	1	n/a

n/a-not applicable

For peer review only
